# Psychological flexibility and cognitive-affective processes in young adults’ daily lives

**DOI:** 10.1038/s41598-024-58598-3

**Published:** 2024-04-08

**Authors:** Marlon Westhoff, Saida Heshmati, Björn Siepe, Christoph Vogelbacher, Joseph Ciarrochi, Steven C. Hayes, Stefan G. Hofmann

**Affiliations:** 1 Department of Psychology, Philipps-University of Marburg, Schulstraße 12, 35032 Marburg, Germany; 2 Department of Psychology, Claremont Graduate University, Claremont, CA USA; 3 Department of Psychology, Philipps-University of Marburg, Marburg, Germany; 4 Institute for Positive Psychology and Education, Australian Catholic University, Sydney, Australia; 5 Department of Psychology, University of Nevada Reno, Reno, NV USA

**Keywords:** Computational science, Psychology, Quality of life

## Abstract

Psychological flexibility plays a crucial role in how young adults adapt to their evolving cognitive and emotional landscapes. Our study investigated a core aspect of psychological flexibility in young adults: adaptive variability and maladaptive rigidity in the capacity for behavior change. We examined the interplay of these elements with cognitive-affective processes within a dynamic network, uncovering their manifestation in everyday life. Through an Ecological Momentary Assessment design, we collected intensive longitudinal data over 3 weeks from 114 young adults ages 19 to 32. Using a dynamic network approach, we assessed the temporal dynamics and individual variability in flexibility in relation to cognitive-affective processes in this sample. Rigidity exhibited the strongest directed association with other variables in the temporal network as well as highest strength centrality, demonstrating particularly strong associations to other variables in the contemporaneous network. In conclusion, the results of this study suggest that rigidity in young adults is associated with negative affect and cognitions at the same time point and the immediate future.

## Introduction

Young adulthood is a pivotal stage marked by significant developmental changes, including the establishment of autonomy, transitioning into new social roles, forming intimate partnerships^1^, and evolving identity and life goals^2^. This period is also characterized by cognitive and emotional maturation^3^, presenting numerous challenges that can impact the daily psychological processes of young adults aged 18–29 years^4,5^. The dynamic nature of this phase provides a unique lens to examine how individuals adapt to these challenges, particularly through the lens of psychological flexibility—a key set of processes that facilitate adaptation to varying contexts^6–11^.

Psychological flexibility is a multidimensional concept that involves the ability to respond adaptively to internal experiences and external situations in a manner consistent with one’s values, adjusting cognitive, affective, and behavioral approaches as needed^8^. It enables young adults to constructively engage with challenging emotions and thoughts through emotional openness and cognitive flexibility^9,12,13^, influencing other psychological processes and contributing to their functional expression. Conversely, psychological inflexibility, characterized by an excessive attachment to cognitions and avoidance of distressing experiences^13^ or over-attachment to supposedly positive ones can be detrimental^14^. Given the contextual nature of psychological flexibility, where internal and external environments are in a constant state of flux^8–10^, its role is especially salient in young adulthood—a time inherently defined by change and the need for adaptation^4,15,16^.

Given the fluid and often tumultuous nature of young adulthood, marked by significant mental health challenges^4,5^, the role of cognitive-affective processes in psychological flexibility warrants closer examination. Cognitive-affective processes refer to the cognitive and emotional aspects of experience and behavior. They include different thinking patterns, such as cognitive fusion, ruminative thinking, or dysfunctional thinking, which can impact how life events are perceived and given meaning. They also include the way individuals feel about their situation and deal with their emotions, for example through the development of acceptance and regulation skills. Therefore, cognitive-affective processes are fundamental to how individuals interpret information based on automatic thoughts or core assumptions^17^, manage and regulate their emotional responses^18,19^, and engage in decision-making that aligns with their values, for example through acceptance or cognitive defusion^20,21^. They are instrumental in shaping an individual’s capacity to navigate stress and adapt to novel situations^22^. The extent to which young adults can effectively confront and adapt to the developmental hurdles they face is significantly influenced by these cognitive-affective processes, which may impact their mental health^23–26^. To assess psychological processes, physiological measures such as heart rate data or brain correlates of psychological flexibility, e.g., associated with shift in attention or perspective taking^27^, are becoming increasingly important. These additional measures will provide complementary insights into an individual’s ability to respond flexibly to internal and external stimuli.

Previous research on psychological flexibility has notable limitations that need to be addressed. Firstly, prior studies have mostly focused on inflexibility^28,29^, which has overshadowed the broader, more nuanced aspects of psychological flexibility that extend beyond the mitigation of clinical distress^30–32^. An evolutionary perspective^33,34^ may be useful for the simultaneous examination of both positive and negative manifestations of psychological flexibility, and for the development of assessment tools with that needed breadth^23^. This involves focusing on the dynamics of variation^29,35^ and rigidity as key mechanisms that influence behavioral adaptability to contextual shifts^36,37^. Variation, characterized by healthy variability and contextual awareness, is essential for adaptive behavior^9,10,38^. In contrast, rigidity, defined as difficulty in shifting from one set way of responding to another in response to contextual events^30,39^, emerges as a central characteristic of mental distress^40–42^. Therefore, flexibility and rigidity are not merely two ends on a continuum, because both rigid and flexible responses may be adaptive and co-occur at different levels in a given time frame.

The reliance on static correlational analyses and cross-sectional designs^7,43,44^ has limited the exploration of within-person changes and the temporal antecedents of maladaptive rigidity and adaptive variability. In order to uncover their potential causal pathways and intricate associations with cognitive-affective processes, research must pivot to longitudinal studies that track the temporal dynamics and individual variability of these core features of psychological flexibility. This in turn requires efficient and concise measurement of the most fundamental aspect of variability/rigidity: an individual’s capacity to change behavior. Such an approach is difficult to achieve with conventional validated questionnaires designed for cross-sectional designs that may stumble in an attempt to cover more features of psychological flexibility as a multidimensional concept.

Whereas some studies highlight the mediating role of psychological flexibility^28,45,46^, there has been an overreliance on self-report measures (e.g., Acceptance and Action Questionnaire^47^) that may not fully capture an individual’s actual ability to modify behavior in real-world contexts (for a brief discussion, see Ciarrochi et al.^48^). Measures of this kind are also ill-suited to longitudinal studies incorporating temporally dense and idiographic assessment, which appears to be required for a more comprehensive exploration of psychological flexibility^9,49,50^.

This study’s objective is to investigate the interplay between cognition, affect, and the capacity to change behavior in young adults during their developmental phase of young adulthood. Specifically, we aimed to answer the question: How is a core component of psychological flexibility – rigidity and variation as distinct components of the capacity to change behavior – associated with both positive and negative cognitive-affective processes in young adults’ daily lives? To achieve this, we utilized intensive longitudinal data (ILD) collected through Ecological Momentary Assessment (EMA)^51^ and employed a dynamic network analytic approach^52^.

Using a dynamic network approach allows us to simultaneously analyze the associations (i.e., edges) between variables (i.e., nodes) reflecting psychological flexibility and cognitive-affective processes over time. By simultaneously considering the interplay between multiple variables, we gain deeper insights into how variables influence each other over time, providing a unique advantage in capturing the complexity of mental health^52,53^. By modeling reciprocal associations between rigidity/flexibility and cognitive-affective processes, rather than just unidirectional relationships, we can capture potential interactions between these processes. In addition to illustrating how the capacity for behavior change and cognitive-affective variables interact within a network, the dynamic network approach also helps identify which variables are comparatively most central. Nodes with high centrality have a comparatively stronger association with the other nodes and therefore may be of greater relevance^54^. The use of ILD may reveal temporal dependencies between variables^55^, which enhances causal inference about the relationships between psychological flexibility and both adaptive and maladaptive cognitive-affective processes^56^. The dynamic network approach addresses the inherent dynamics of variation and rigidity as key mechanisms of adaptability to contextual change, making it advantageous over static correlational analyses. This allows us to study the temporal changes and dynamic interplay of cognitive-affective processes. In addition, it considers individual variability by estimating individual networks instead of relying on aggregated group patterns. This approach enhances generalizability of our findings, as it is based on the aggregation of individual-specific data^57,58^.

Leveraging the dynamic network approach, we hypothesize that positive and negative processes are interconnected and mutually reinforcing, reflecting their interdependence. Consistent with prior research^23,59^, we anticipate that in a complex network, rigidity will have comparatively stronger contemporaneous and temporal associations with negative, rather than positive, cognitive-affective processes. At the same time, we assume that variation will have stronger contemporaneous and temporal associations with positive cognitive-affective processes. Furthermore, building on previous findings^60–62^, we hypothesize that rigidity will be a particularly central node compared to variation and will have a correspondingly stronger association with the other processes under investigation^47,61^.

## Method

### Procedure

We collected data with EMA^51^ as part of a larger study. This study consisted of three phases: a baseline assessment, the EMA phase, and a post-assessment. For the present analyses, only data from the EMA phase were used. The EMA phase took place between September 14th 2022 and July 27th, 2023. Each individual EMA assessment spanned 21 days. Participants received prompts on their smartphones five times per day, following a semi-random schedule. Prompts were scheduled within participants’ self-reported waking hours and were delivered randomly with a normal distribution (*SD* = 0.63 h) around the scheduled time, with intervals of 3 h throughout the day and at least 30 min apart. Each assessment lasted around 3.5 min, and participants were required to respond to the prompts within 120 min, after which they expired. Prompts were conducted using the *Ethica Data* platform, available on both iOS and Android, which participants installed on their smartphones. Participants were directed to the *Qualtrics* platform via *Ethica* to respond to relevant questions.

### Participants

One hundred and twenty-five participants enrolled in the study. They were recruited through an online portal as well as flyers and posters displayed on the university campus. Eligible participants were required to be students, be over 18 years of age, and either be native German speakers or possess native-level fluency in German. Participants received reimbursement based on their response rate, with higher payment per prompt for higher response rates. The highest possible remuneration was 225 €. Eleven participants from the initial 125 were excluded from the dataset due to improper responses to survey items used for validation purposes or had too low a response rate (defined below), resulting in an analysis involving data from 114 participants. The overall mean compliance rate was 92.88% (*SD* = 8.13; range 41.90–100). The study was approved by the Ethics Committee of the Department of Psychology of the Philipps-University of Marburg (Reference: 2022-22v). All participants provided informed consent after receiving complete information about the study.

Among the 114 participants included, 80 identified as female, while 34 identified as male; none identified as neither male nor female. The mean age was 23.77 (*SD* = 2.52). The compliance rate among included participants was 93.54% (*SD* = 5.67; range 71.43–100). The majority of participants reported being in a relationship (63%), while fewer were single (36%) or married (1%). Regarding living status, the vast majority (68%) resided in shared apartments, and 68% were bachelor students.

### Measures

#### Time-invariant variables

Participants provided self-reported information regarding their gender, age, relationship status, living situation, and educational attainment. Descriptive statistics of the time-invariant variables can be found in Table 1.

**Table 1:** Demographics and Characteristics.

	*N* (%)
Age (Mean, *SD*)	23.77 (2.52)
Gender	
Male	34 (29.82)
Female	80 (70.18)
Relationship status	
Single	41 (35.96)
Relationship	72 (63.16)
Married	1 (0.88)
Living situation	
With friends	78 (68.42)
With partner	16 (14.04)
Alone	9 (7.89)
With parents	7 (6.14)
Other	4 (3.51)
Educational attainment	
Bachelor students	79 (69.30)
Master’s students	35 (30.70)

SD, standard deviation; N*,* number of participants.

#### Time-variant variables: item selection procedure and response scale

The item selection procedure employed in this study was devised to address pertinent concerns related to dynamic psychological processes of change. The reported items are constituents of a comprehensive EMA questionnaire battery, which, in turn, forms a segment of a more extensive study comprising baseline and post-surveys. All measures used in the study are provided in the supplementary materials. For the present publication only a subset of selected items specifically relevant to our research questions are selected and listed. Complete data is accessible in the supplementary materials.

To answer relevant scientific questions, items assessing psychological flexibility, cognition, and affect were selected from the entire questionnaire battery. Items were assessed using the *Process-Based Assessment Tool* (PBAT)^23^. The PBAT is an item-pool designed for intensive longitudinal assessment, targeting biopsychosocial processes of change as conceptualized within the “extended evolutionary meta-model” of process-based therapy^33^. It consists of 18 items that assess cognition, affect, attention, social connection, motivation, overt behavior, physical health, retention, and flexibility/variation. Relevant to our research, the most generic component of psychological flexibility – adaptive variation and maladaptive rigidity in the capacity to change behavior – is represented by two items: “I am feeling stuck and unable to change my ineffective behavior” (rigidity) and “I am able to change my behavior, so that it helps my life” (variation). Affect was assessed with items “I am not finding an appropriate outlet for my emotions” (negative) and “I am able to experience a range of emotions appropriate to the moment” (positive). Cognition was measured using statements “My thinking is getting in the way of things that are important to me” (negative) and “I am using my thinking in ways that help me live better” (positive). Participants were asked to indicate their level of agreement with each statement based on their current feelings and behaviors on a scale from 0 to 100.

Thus in this study we focused on and measured the “bottom line” feature of psychological flexibility^63^ that is particularly consistent with the contextualistic and evolutionary approach that underlies the concept^64^ – can an individual display healthy forms of variation and avoid maladaptive rigidity in what they actually do. The items of the PBAT are designed to provide an efficient and concise EMA-style measure of this feature – as well as cognitive and affective domains. The PBAT represents a useful complement to more comprehensive, validated questionnaires that are more likely to be used in a cross-sectional context. The PBAT is the most specific and comprehensive measure available to assess psychological flexibility in an EMA setting.

### Data analysis

Data preprocessing was performed using Python version 3.11.3^65^, time-series analyses were conducted using the statistical software R version 4.1.2^66^. The data and syntax used for the analyses are available in the supplementary materials.

#### Data preprocessing

To ensure data quality, we conducted several preprocessing steps. First, we ensured that only participants with a response rate ≥ 70% (i.e., at least 73 completed measurements) were included in the analyses. Additionally, care was taken to only include those prompts with a relative speed index (RSI) 63 value ≤ 2, which is a measure of completion speed and serves as an indicator of conscientious working. The RSI is computed by dividing the completion time of a prompt by the median completion time of the participant. RSI values exceeding 1 indicate faster completion, while values below 1 denote slower completion. Prompts with TIME_RSI values above 2 indicate very quick responses and should be viewed critically. Furthermore, attentiveness was assessed using a verification item (“Please just click tend to agree here”) asked at the post-assessment, along with questions regarding honest answering. Four participants were excluded from the analysis who indicated at post-assessment that they had not answered conscientiously, while two participants were excluded for providing false responses to the verification item. Additionally, two participants were excluded due to a poor compliance rate, leaving 114 participants for all further analyses. Participants were required to answer the questions and interact with the slider to progress to the next question and complete the prompt. Consequently, missing values only occurred when an entire prompt was missing by a participant due to not responding. The analysis technique explained below assumes stationarity. Therefore, the individual time series were detrended by fitting fixed-effects linear regression models to each variable, regressing out linear trends based on day number and categorical effects on the measurement per day, using an alpha of 0.05.

**Figure 1:**
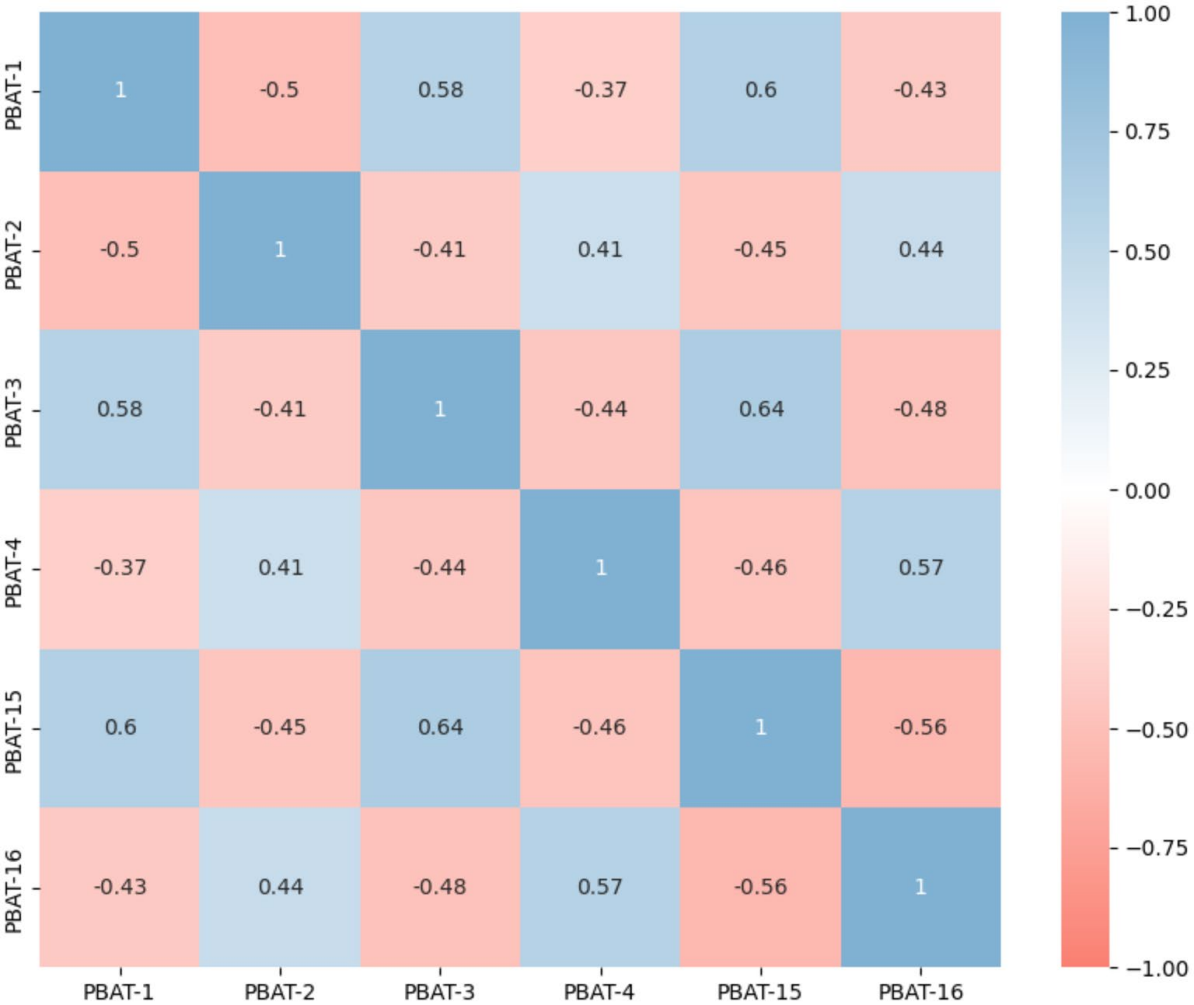
Correlation Matrix for the Items Under Investigation. *Note* PBAT1 = negative affect, PBAT2 = positive affect, PBAT3 = negative cognition, PBAT4 = positive cognition, PBAT15 = rigidity, PBAT16 = variation; Figure illustrates the strength of correlations between variables. Color intensity represents strength of correlation, from dark red (strong negative) to dark blue (strong positive).

To avoid topological overlap of items that were selected based on theoretical premises, especially in light of positive and negative worded items assessing the same construct, we evaluated the presence of multicollinearity and the possibility of node redundancy by calculating zero-order correlations between the six variables of interest (see Fig. 1). Correlations among items exhibit a range from moderately to large^67^, thus reinforcing the hypothesis of interconnected psychological processes^50^. However, none of the bivariate correlations exceeded standard cut-offs thresholds for multicollinearity, signifying the absence of item redundancy (r ≥ 0.80)^68^.

#### Dynamic network analysis

The main objective of this study was to construct dynamic network models to investigate the dynamic associations between flexibility in the capacity to change behavior and cognitive-affective processes as young adults lived their daily lives. Networks are visually represented through graphs comprising nodes (variables) and edges (statistical relationships). Each of the processes was considered a node in the network.

For the estimation of dynamic network models, we used multilevel vector autoregression (VAR) with the R package *mlVAR*^69^. In a two-step procedure, *mlVAR* first obtains directed within-person temporal effects by performing node-wise multilevel regressions where each node is predicted by itself and all other nodes at the previous time point. The resulting temporal networks display directed edges, reflecting directed partial regression coefficients between two variables while controlling for all other relationships. In the second step, node-wise multilevel regressions with the residuals of the first step are conducted to obtain undirected contemporaneous networks. These contain partial correlations between the variables, controlled for the lag-1 temporal associations. Thickness and saturation of edges within graphical depictions indicate the strength of relationships, with positive edges shown in blue and negative edges in red. Both temporal and contemporaneous network structures were modeled for all nodes of interest.

For temporal networks, we computed node centrality using the centrality indices in-strength (IS) and out-strength (OS). In-strength centrality refers to the summation of all absolute values of the weighted edges directed towards a node, while the out-strength parameter of a node indicates the summation of the absolute values of all outgoing edges. Regarding contemporaneous networks, node centrality was determined using strength centrality. We investigated the stability of centrality measures with a case-drop bootstrap. We repeatedly dropped 20% of the sample at random 1000 times, refitted our models and recalculated the centrality measures. We then assessed the rank consistency of the nodes.

## Results

### Descriptive statistics

**Figure 2:**
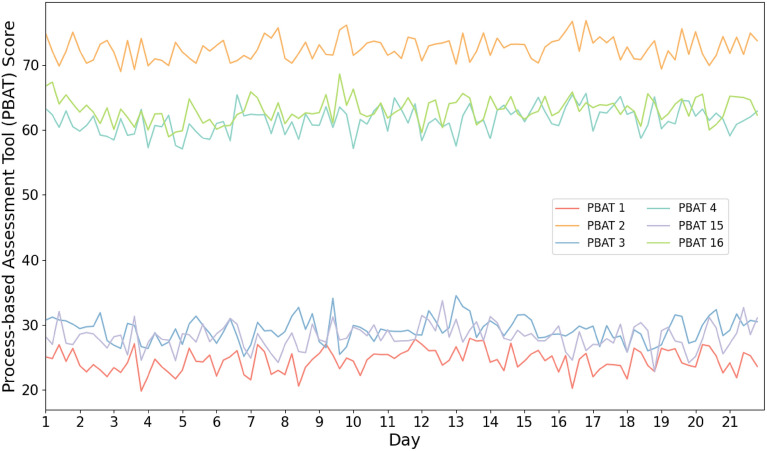
Temporal Evolution of Psychological Processes Averaged Across the Study Cohort. *Note* PBAT = Process-Based Assessment Tool; PBAT 1 = negative affect, PBAT 2 = positive affect, PBAT 3 = negative cognition, PBAT 4 = positive cognition, PBAT 15 = rigidity, PBAT 16 = variation.

Table 2 displays descriptive data representing the recorded processes for the group over time. Results indicate that, on average, participants experienced higher levels of positive affective and cognitive processes and variation as opposed to rigidity. Additionally, the sample tended to exhibit lower variability in their positive processes versus negative processes, as evidenced by lower standard deviations. Figure 2 illustrates the temporal progression of group processes, with both positive and negative processes exhibiting comparable patterns.

**Table 2:** Descriptives of process-based assessment tool items.

	Neg. affect	Pos. affect	Neg. cognition	Pos. cognition	Rigidity	Variation
M	24.50	72.51	29.26	61.50	28.20	63.01
SD	25.44	21.20	25.32	22.22	26.30	21.82
Percentile						
25%	2.00	63.00	10.00	46.00	6.00	50.00
50%	17.00	75.00	23.00	66.00	21.00	67.00
75%	35.00	88.00	42.00	77.00	43.00	78.00

M*,* Mean; SD*,* standard deviation; neg. negative; pos. positive.

### Network model

#### Contemporaneous network models

**Figure 3:**
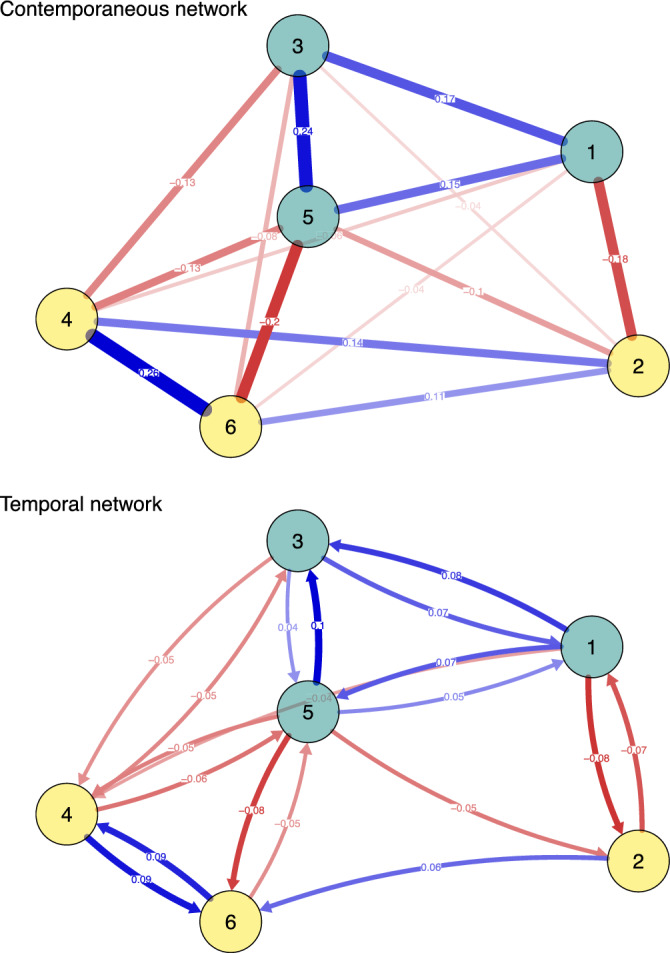
Networks of variation/rigidity, cognition, and affect. *Note* 1 = negative affect, 2 = positive affect, 3 = negative cognition, 4 = positive cognition, 5 = rigidity, 6 = variation; non-significant edges are omitted. In the contemporaneous network, all edges are non-directed. In the temporal network, all edges are directed. Thickness of edges indicates strength of associations. Blue edges indicate positive associations, red edges indicate negative associations.

Figure 3 displays contemporaneous and temporal networks depicting the associations between psychological flexibility and cognitive-affective processes. Non-significant edges in the network were omitted from visualization. Only statistically significant relationships are described below.

In the contemporaneous network, which represents relationships among items at the same time point, several anticipated associations among items were identified. Notably, all nodes (i.e., psychological processes) were interconnected within the same time window (Fig. 3). As hypothesized, positive processes showed positive associations with each other, with a range of coefficients from *r*_partial_ = 0.11 to r_partial_ = 0.26. Negative processes were also positively related to each other, with a range of coefficients from *r*_partial_ = 0.15 to *r*_partial_ = 0.25. Positive processes were found to be negatively related to negative processes. Descriptively, rigidity exhibited stronger positive associations with negative processes, whereas variation showed stronger positive associations with positive processes.

In terms of centrality within the contemporaneous network, rigidity comparatively demonstrated the highest strength centrality (S = 0.83), matching our assumptions. This indicates that rigidity had comparatively the highest degree of connectivity within the contemporaneous network. Centrality estimates of the contemporaneous network can be found in the supplementary materials (Table 3). Stability analyses of the centrality indices indicated that rigidity had the highest strength centrality in all iterations of the case-drop bootstrap.

**Table 3:** Centrality indices for variables under investigation in temporal and contemporaneous network.

	Temporal		Contemporaneous
	In-strength	Out-strength	Strength
PBAT1	0.18	0.27	0.61
PBAT2	0.14	0.17	0.57
PBAT3	0.26	0.15	0.67
PBAT4	0.23	0.20	0.70
PBAT15	0.22	0.33	0.83
PBAT16	0.24	0.14	0.69

PBAT1, negative affect; PBAT2, positive affect; PBAT3, negative cognition; PBAT4, positive cognition; PBAT15, rigidity; PBAT16, variation.

#### Temporal network models

In the temporal network structure, which depicts lag-1 relationships from one 3-h measurement period to the subsequent one, the results of the *mlVAR* network analyses revealed significant directed relationships both within and between nodes of the dimensions of processes, consistent with our assumptions (Fig. 3). Negative affective processes, negative cognitive processes, and rigidity were positively related to each other, with significant associations ranging from *β* = 0.05 to *β* = 0.10. This indicates that negative processes at a previous time point predicted an increased level of negative processes at the next measurement time. Regarding positive processes, bidirectional links were observed between variation and cognition, ranging from *β* = 0.09 to *β* = 0.10. Furthermore, positive affect at a previous time point predicted flexibility at the next measurement time (*β* = 0.06), but not vice versa. Moreover, processes of the same construct (e.g., cognitive processes) negatively predicted each other at the next time point. Interestingly, rigidity was not only primarily related to negative cognitive-affective processes but likewise to positive processes.

Rigidity had the highest centrality in the temporal network, considering out-strength centrality (OS = 0.33), but not considering in-strength centrality (IS = 0.22). Negative cognition (IS = 0.26), and variation (IS = 0.24) comparatively showed the highest in-strength centrality, suggesting that these nodes were most associated with other nodes at earlier time points. Notably, only positive affective processes did not predict rigidity at the next time point. The centrality estimates of the temporal network can be found in Supplementary Table 3. In the case-drop bootstrap, negative cognition had the highest in-strength centrality in 74.9% of iterations, while rigidity had the highest out-strength centrality in 88.8% of iterations.

## Discussion

To explore the functional relationships among a core feature of psychological flexibility – variation and rigidity in the capacity to change behavior – and positive and negative change processes (i.e., cognition, affect) in young adults, we utilized a time-series network approach. We examined both the temporal and contemporaneous network structures involving variation, rigidity, and negative and positive cognitive-affective processes.

In the temporal network, rigidity exhibited the highest out-strength. It predicted future experiences of negative cognitive-affective processes while inversely predicting future experiences of positive cognitive-affective processes as well as variation. This suggests that an individual’s overall flexibility at one point may be associated with the subsequent experiences of specific cognitive-affective processes. This finding aligns with our hypothesis and previous literature, which has underscored the pivotal role of rigidity in psychopathology^30,40,49,62^ and aligns with our assumption that rigidity (OS = 0.33) would have a more central role compared to variation (OS = 0.14). However, this cannot be stated for in-strength, with respect to which variation (IS = 0.24) and rigidity (IS = 0.22) are roughly equal. However, the interpretation of the centrality estimates should be approached cautiously, given their similarity and the uncertainty in the estimation procedure.

Interestingly, the node representing variation showed the least connectivity with other nodes in the network. It had bidirectional connections only with rigidity and positive cognition. Positive affect also predicted variation. In view of the comparatively high in-strength, variation might be more likely to be considered an endpoint of connections in the network. Moreover, it is notable that rigidity appears to have relatively stronger outgoing connections to other variables compared to incoming ones in the temporal network. This discrepancy could give insight into the nature of rigidity. For example, rigidity may be less influenced by cognitive and affective processes than it influences them. In addition, it could act more as a starting point for processes and a central control point in the network activities. Further research is needed to explore if rigidity behaves similarly in the context of psychopathological symptoms. However, the differences between absolute centrality values are relatively small. While we have attempted to account for uncertainty, it should be noted that these differences may merely represent measurement noise.

In the contemporaneous network, nodes related to positive or negative behavior exhibited positive correlations with each other (e.g., positive cognition, affect, and variation), and nodes with opposite valence showed negative associations (e.g., positive cognition and negative affect). Furthermore, the results support our assumption that rigidity exhibits stronger associations with negative processes, whereas variation demonstrates stronger associations with positive processes. The positive associations within positive and negative processes, respectively, align with prior research, which has demonstrated robust positive associations among processes of the same valence and negative relationships between processes of opposite valence^23,38,70,71^.

The findings offer valuable insights into the dynamic interplay between a core feature of psychological flexibility and cognitive-affective processes in young adults. The centrality of rigidity emphasizes its potential relevance in shaping subsequent psychological experiences. The results suggest that rigidity may play a relevant role in driving the progression of psychological processes over time. Constricted psychological flexibility, characterized by feeling stuck and unable to change ineffective behaviors, may lead to subsequent maladaptive cognitive-affective processes. This underscores the potential relevance of impaired psychological flexibility for mental health.

As expected, both the contemporaneous and temporal networks display extensive connections between nodes, highlighting the highly interconnected nature of psychological processes^52,72^. In the temporal network, numerous feedback loops with nodes showing reciprocal connections to one another are evident. These feedback loops can contribute to system stability or instability^73–75^. They can lead to the stabilization and maintenance of both adaptive and maladaptive states. It is well-established that feedback loops between nodes significantly influence system stability, which may explain the persistence of mental disorders^76,77^. The identification of this aspect of psychological flexibility as a core process integrated within feedback loops with psychological processes such as cognition and affect holds great relevance for researchers and practitioners. Consequently, a key implication could be to prioritize the examination of this feature of psychological flexibility in future studies, given its pronounced associations with the entire system of cognitive-affective processes.

The findings of this study hold significant relevance for young adults in their everyday lives as they provide valuable insights into the relationships between psychological processes that may impact their emotions, thoughts, and behaviors. Results show that the psychological processes experienced by young adults are closely connected within short time intervals. The central role of rigidity indicates its pivotal role in shaping subsequent psychological experiences and underscores its importance in understanding mental health in this population. Young adults who tend to exhibit rigidity, feeling stuck and unable to change ineffective behaviors, may be more prone to experiencing negative psychological states, especially in light of this critical developmental period^2,78,79^. Recognizing the significance of psychological flexibility can empower young adults to cultivate more adaptive, flexible coping strategies, which may also improve mental health.

As previously mentioned, there is a need for the simultaneous examination of both positive and negative manifestations of psychological flexibility more generally and identification of its primary determinants^38,80^. Additionally, the development of effective measures for assessing psychological flexibility in longitudinal studies is crucial for identifying meaningful processes of change at an individual level^81,82^. A key objective for researchers and practitioners in clinical settings is to manipulate elements within the network to steer the system toward a trajectory leading to a desired target state^83,84^. This necessitates identifying elements within the network that can efficiently drive functional changes. The present study explores how rigidity and variation as features of behavior change impact cognitive-affective processes. It suggests that a more nuanced approach to understanding this core feature of psychological flexibility should be considered in future research and therapy. This newfound understanding has the potential to improve the development of more effective interventions targeting the capability to flexibly change behavior, thereby positively influencing psychological processes, such as cognition and affect. However, future studies in clinical settings will be necessary to confirm these assumptions and explore the impact of psychological flexibility in areas beyond cognition and affect as well.

One limitation of this study is the restricted sample, which consisted solely of students from a single institution. Consequently, future research should aim to explore associations between psychological flexibility, cognition, and affect in a more diverse and representative sample encompassing a broader range of mental disorders. Another limitation pertains to the reliance on self-report measures for assessing psychological constructs. Such self-report data may be influenced by social desirability or lack of awareness, potentially impacting the observed relationships’ strength. In the context of psychological flexibility, however, there are currently few alternatives to self-report ratings. In future work, it may be beneficial to consider supplementing self-report with other measures, such as physiological data (e.g., heart rate variability, metabolic flexibility, blood pressure), brain correlates, and overt behaviors while recording the context.

Furthermore, we assessed the stability of centrality measures with a case-drop bootstrap. It is unclear how well this method performs in stability assessment, and there are no clear guidelines on how to best assess uncertainty in centrality estimates in dynamic networks^85^. Another important limitation is that mlVAR is a multilevel model that assumes that network edges of different individuals come from a common distribution. It is possible that there are subgroups, or that individual-level effects are so different from one another that the use of more idiographic techniques (such as uSEM or GIMME^86^) would be more appropriate. Ideally, such subgroup analyses would involve large data sets to find robust subgroups. As a first step, however, it seemed important to examine the dynamic network in a fashion that is more similar to current mainstream analytic practice.

While our hypothesis posits that incorporating the temporal dimension will enhance our comprehension of the temporal relationships among psychological flexibility, cognition, and affect, it is important to recognize that temporal associations and node centrality measures do not equate to causality^87–89^. Therefore, cautious interpretation is warranted, and future research should strive to establish causal relationships using longitudinal data and assess causal inference assumptions^90^.

Moreover, it is vital to consider that most network analyses, including the current study, operate under the assumption of a constant network structure, disregarding potential changes of mean or variance over time (i.e., stationarity). However, this assumption does not align with the dynamic nature of interventions and psychotherapeutic processes, where individuals’ experiences and responses evolve over time. To address the assumption of stationarity, future studies could employ innovative time-varying network models, capturing fluctuations in associations between variables across time points^91^, thus providing a more accurate representation of the evolving psychological processes. However, these analysis techniques need a large amount of data^92^ and are therefore often infeasible in typical psychological applications. Time-varying network models become particularly pertinent when examining the experimental influence of psychological flexibility on cognitive-affective processes.

Finally, participants received a relatively high payment for participation in the EMA phase. While it was also intended to compensate for the burden of wearing wearables required for future analyses, the reimbursement was based on response rate, with higher payment per prompt for higher response rates. This could have led to lower-quality responses. However, the remuneration system was intentionally selected to produce a significant amount of data, resulting in more reliable and meaningful results. To address the risk of invalid data, strict preprocessing steps were taken as described. Additionally, participants were given the option to anonymously indicate at the end if they had responded carelessly (such as to receive higher payment). The participants were informed that their payment would not be influenced by the disclosure. Data from participants who disclosed careless responding were excluded from the analyses.

## Conclusion

The present study contributes to the growing body of literature indicating the significance of psychological flexibility as a crucial transdiagnostic process for psychological phenomena among young adults. Overall psychological flexibility and its relation to cognitive-affective processes may play a crucial role in mental health. Future research should examine specific intervention strategies to target such maladaptive networks, such as through network control theory^93^.

### Supplementary Information

Supplementary Information 1.

Supplementary Information 2.

Supplementary Information 3.

## Data Availability

All data generated or analyzed during this study are included in this published article (and its Supplementary Information files).
