# Supplementary material for: Psychological flexibility and cognitive-affective processes in young adults’ daily lives
Source: Sci Rep. 2024 Apr 8;14:8182. doi: 10.1038/s41598-024-58598-3 (PMC11001944; doi:10.1038/s41598-024-58598-3)
Supplement: Supplementary file 3 — Supplementary Information 3. [file 41598_2024_58598_MOESM3_ESM.pdf]

```r
# ---------------------------------------------------------------------------
# -------------------Loading packages & Data ------------------------------
# ---------------------------------------------------------------------------
required_packages <- c(
  "ggplot2",
  "dplyr",
  "mlVAR",
  "qgraph",
  "bootnet",
  "reshape",
  "viridis",
  "lm.beta",
  "networktools",
  "igraph",
  "here")

# Check if packages are already installed
missing_packages <- setdiff(required_packages, installed.packages()[,"Package"])

# Install missing packages
if (length(missing_packages) > 0) {
  install.packages(missing_packages)
}

# Load packages
library(ggplot2)
library(dplyr)
library(mlVAR)
library(qgraph)
library(bootnet)
library(reshape)
library(viridis)
library(lm.beta)
library(networktools)
library(igraph)
library(here)


figs <- "./figures/" # figure directory
datapath <- "./data/" # data directory


# ---------------------------------------------------------------------------
# -------------------Load data ---------------------------------------------
# ---------------------------------------------------------------------------

data_in<- read.csv(file='Network Analysis Psychological Flexibility_Data.csv',header=TRUE)
data_in_new = subset(data_in, select =
c(Scheduled.Time,Response.Time,VID,Day,Session,Session.ID,PBAT.1,PBAT.2,PBAT.3,PBAT.4,PBAT.5,PBAT.6,PBAT.7,PBAT.8,PBAT.9,P

data_in_new[data_in_new == -1] <- NA

colnames(data_in_new) <-
c("Scheduled.Time","Response.Time",'VID','day','beep','SessionID',"PBAT1","PBAT2","PBAT3","PBAT4","PBAT5","PBAT6","PBAT7",


# Inspect data ---------------------------------------------------------------
# Calculate within-person statistics and variability thereof
data_in_new %>%
  dplyr::group_by(VID) %>%
  dplyr::summarize(across(all_of(rel_vars),
                          list(mean = mean, sd = sd),
                          na.rm = TRUE,
                          .names = "{.col}_{.fn}")) %>%
  ungroup() %>% View()


## Overall distributions
data_in_new %>%
  dplyr::select(all_of(rel_vars)) %>%
  tidyr::pivot_longer(cols = everything()) %>%
  ggplot(aes(x = value)) +
  geom_histogram()+
  facet_grid(.~name)+
  theme_classic()



# Missing data ---------------------------------------------------------------
data_miss <- data_in_new %>%
  dplyr::select(VID, day, beep, all_of(rel_vars)) %>%
  dplyr::mutate(miss_n = rowSums(is.na(.[, rel_vars])),
                miss_ind = ifelse(rowSums(is.na(.[, rel_vars])) == length(rel_vars), 1, 0))

# check if less than full row of missings occurs
data_miss %>%
  distinct(miss_n)   # no

# Summarize missingnes
data_miss %>%
  group_by(VID) %>%
```

```
  dplyr::summarize(prop_miss = sum(miss_ind)/n()) %>%
  View()



# ----------------------------------------------------------------------------
# --------------------Detrend data -----------------------------------------
# ----------------------------------------------------------------------------

# Alpha to detrend:
alpha <- 0.05

# Variables to investigate:

vars <-
c("PBAT1","PBAT2","PBAT3","PBAT4","PBAT5","PBAT6","PBAT7","PBAT8","PBAT9","PBAT10","PBAT11","PBAT12","PBAT13","PBAT14","PB

# Labels:
varLabs <-
c("PBAT1","PBAT2","PBAT3","PBAT4","PBAT5","PBAT6","PBAT7","PBAT8","PBAT9","PBAT10","PBAT11","PBAT12","PBAT13","PBAT14","PB

names(data_in_new)[names(data_in_new) %in% vars] <- varLabs

#Remove items:
data_in_new <- data_in_new %>% select(-PBAT5,-PBAT6,-PBAT7,-PBAT8,-PBAT9,-PBAT10,-PBAT11,-PBAT12,-PBAT13,-PBAT14,-
PBAT17,-PBAT18,-STOPD1,-STOPD2,-STOPD3,-STOPD4,-STOPD5)
varLabs <- varLabs[!varLabs %in%
c("PBAT5","PBAT6","PBAT7","PBAT8","PBAT9","PBAT10","PBAT11","PBAT12","PBAT13","PBAT14","PBAT17","PBAT18","STOPD1","STOPD2"


# Data frame with empty values for fitted effects (all):
fitted_all <- expand.grid(
  beep = seq(min(data_in_new$beep),max(data_in_new$beep)),
  day = seq(min(data_in_new$day),max(data_in_new$day))
)

# Data frame with empty values for day trends:
fitted_day <- data.frame(
  day = seq(min(data_in_new$day),max(data_in_new$day))
)

# Data frame with empty values for beeps:
fitted_beep <- data.frame(
  beep = seq(min(data_in_new$beep),max(data_in_new$beep))
)

# Data frame to store p-values:
p_values <- data.frame(
  var = c("day", "beep")
)

# Also empty data frame list for test statistics:
testStatistics <- list()
coefficients <- list()
stdcoefficients <- list()

# Make beep variable factor in dataset:
data_in_new$beepFactor <- factor(data_in_new$beep, levels = 1:5, labels = c("1","2","3","4","5"))
fitted_all$beepFactor <- factor(fitted_all$beep, levels = 1:5, labels = c("1","2","3","4","5"))
fitted_beep$beepFactor <- factor(fitted_beep$beep, levels = 1:5, labels = c("1","2","3","4","5"))

# Make day variable for dates:
data_in_new$date <- as.Date("2022-09-14") + data_in_new$day
fitted_all$date <- as.Date("2022-09-14") + fitted_all$day
fitted_day$date <- as.Date("2022-09-14") + fitted_day$day

# Add midpoints as time variable:
data_in_new$midTime <- as.character(factor(data_in_new$beep, levels = 1:5, labels =
c("8:30","11:30","14:30","17:30","20:30")))
data_in_new$midTime <- as.POSIXct(paste(data_in_new$beep,data_in_new$midTime), format = "%Y-%m-%d %H:%M", tz =
"Europe/Amsterdam")

fitted_all$midTime <- as.character(factor(fitted_all$beep, levels = 1:5, labels =
c("8:30","11:30","14:30","17:30","20:30")))
fitted_all$midTime <- as.POSIXct(paste(fitted_all$date,fitted_all$midTime), format = "%Y-%m-%d %H:%M", tz =
"Europe/Amsterdam")

# Data frame to store detrended data:
data_detrended <- data_in_new

# Fix curves:
for (v in seq_along(varLabs)){
  formula <- as.formula(paste0(varLabs[v], " ~ 1 + day + factor(beep)"))
  lmRes <- lm(formula, data = data_in_new)

  # Fixed effects:
  fixed <- coef(lmRes)

  # make zero if not significant at alpha:
  p_values[[varLabs[v]]] <- anova(lmRes)[["Pr(>F)"]][1:2]
  if (p_values[[varLabs[v]]][1] > alpha){
    fixed[2] <- 0
```

```
  }
  if (p_values[[varLabs[v]]][2] > alpha){
    fixed[3:5] <- 0
  }

  # Add to DFs:
  fitted_all[,varLabs[v]] <- fixed[1] + fixed[2] * fitted_all[["day"]]  +  fixed[3] * (fitted_all[["beep"]] == 1)  +
    fixed[4] * (fitted_all[["beep"]] == 2) + fixed[5] *  (fitted_all[["beep"]] == 3)

  fitted_day[,varLabs[v]] <- fixed[1] + fixed[2] * fitted_day[["day"]]

  fitted_beep[,varLabs[v]] <- fixed[1] + fixed[2] * median(fitted_day[["day"]]) +  fixed[3] * (fitted_beep[["beep"]] ==
1)  +
    fixed[4] * (fitted_beep[["beep"]] == 2) + fixed[5] *  (fitted_beep[["beep"]] == 3)

  # Detrend data:
  data_detrended[,varLabs[v]] <- data_in_new[,varLabs[v]] - (fixed[1] + fixed[2] * data_in_new[["day"]]  +  fixed[3] *
(data_in_new[["beep"]] == 1)  +
                                                          fixed[4] * (data_in_new[["beep"]] == 2) + fixed[5] *
(data_in_new[["beep"]] == 3))

  ids <- rownames(anova(lmRes))
  testStatistics[[v]] <- cbind(data.frame(var = varLabs[v], effect = ids), anova(lmRes))

  coefficients[[v]] <- data.frame(
    var = varLabs[v],
    type = names(coef(lmRes)),
    coef = coef(lmRes),
    std = coef(lm.beta(lmRes))
  )
}


# ------------------------------------------------------------------------
# ----------------------- Estimate network model----------------------------
# ------------------------------------------------------------------------

# Estimate network using multilevel VAR model
res_correlated <- mlVAR(data_detrended,
             vars=varLabs,
             idvar="VID",
             dayvar="day",
             beepvar="beep",
             lags = 1,
             temporal = "correlated",
             contemporaneous = "correlated",
             nCores = 12)

#######################################################
################ Calculate CIs ########################
#######################################################

# Calculate confidence intervals for edge weights
install.packages("tidyverse")
library(tidyverse)

# save edge names
edge_names <- dimnames(res_correlated$results$Beta$mean)

# Convert array to matrix and name correctly
edges <- apply(res_correlated$results$Beta$mean, 2, c)
dimnames(edges) <- edge_names[1:2]

# Convert array to matrix and name correctly
se_edges <- apply(res_correlated$results$Beta$SE, 2, c)
dimnames(se_edges) <- edge_names[1:2]

conf_level <- 0.95

# take absolute value
z_crit <- abs(qnorm((1 - conf_level) / 2))
lower_bound <- edges - z_crit * se_edges
upper_bound <- edges + z_crit * se_edges

# Create dataframe with edge weights and confidence intervals
df_edge <- edges %>%
  as.data.frame() %>%
  rownames_to_column() %>%
  pivot_longer(cols = !rowname,
               names_to = "iv",
               values_to = "estimate") %>%
  dplyr::rename(dv = rowname)

# matrices for dataframes
# dv = dependent variable, iv = independent variable
df_lb <- lower_bound %>%
  as.data.frame() %>%
  rownames_to_column() %>%
  pivot_longer(cols = !rowname,
               names_to = "iv",
               values_to = "lower_bound") %>%
  dplyr::rename(dv = rowname)
```

```
df_ub <- upper_bound %>%
  as.data.frame() %>%
  rownames_to_column() %>%
  pivot_longer(cols = !rowname,
               names_to = "iv",
               values_to = "upper_bound") %>%
  dplyr::rename(dv = rowname)

# merge
df_conf_intervals <- df_edge %>%
  left_join(df_lb, by = c("dv", "iv")) %>%
  left_join(df_ub, by = c("dv", "iv"))


# Output of the data frame with the edge weights and confidence intervals
print(df_conf_intervals)

write.csv(df_conf_intervals, file = "p_values_edges.csv", row.names = FALSE)



##############################################################################
################################ Plot ########################################
##############################################################################

#names <- c("PBAT1","PBAT2","PBAT3","PBAT4","PBT15", "PBAT16"
#PBAT 1 & 2 = Affect; PBAT 3 & 4 = Cognition; PBAT 15 & 16 = Variation
names <- c("PBAT1","PBAT2","PBAT3","PBAT4","PBAT15","PBAT16")


# Get networks:
cont <- getNet(res_correlated, "contemporaneous", layout = "spring", nonsig = "hide", rule = "and")
bet  <- getNet(res_correlated, "between", layout = "spring", nonsig = "hide", rule = "and")
temp <- getNet(res_correlated, "temporal", layout = "spring", nonsig = "hide")


shape_list <- c("circle","circle","circle","circle","circle","circle")

L <- averageLayout(cont,bet,temp)

pdf("figure_PBAT.pdf", width = 4, height = 8)

#pdf(paste0(figs, "figure.pdf"), width=6, height=2.5)

layout(matrix(c(1,1,2,2,2), nc=5, byrow = TRUE)) # 40% vs 60% widths
par(mfrow = c(3, 1), mar = c(4, 4, 4, 4))  # Adjust 'mar' for proper margins
n1 <- qgraph(cont, layout = L,
             title="Contemporaneous network", theme='colorblind', negDashed=FALSE,
             legend=FALSE, nodeNames = names, labels=c(1:6),
             vsize=14,color=viridis_pal(alpha = 0.5)(3)[2:3],edge.labels = TRUE)
n2 <- qgraph(temp, layout = L,
             title="Temporal network", theme='colorblind', negDashed=FALSE, diag=FALSE,
             legend.cex=0.5, legend=FALSE, nodeNames = names, labels=c(1:6),
             vsize=14,color=viridis_pal(alpha = 0.5)(3)[2:3], asize=6, curve=-0.8,
curveAll=T,shape=shape_list,edge.labels = TRUE )
n3 <- qgraph(bet, layout = L,
             title="Between network", theme='colorblind', negDashed=FALSE, diag=FALSE,
             legend.cex=0.5, legend=FALSE, nodeNames = names, labels=c(1:6),
             vsize=14,color=viridis_pal(alpha = 0.5)(3)[2:3], asize=6, curve=-0.8, curveAll=T,shape=shape_list
,edge.labels = TRUE)
par(mfrow = c(1, 1), mar = c(5, 4, 4, 2) + 0.1)
dev.off()



# ---------------------------------------------------------------------------
# -----------------------Calculating centrality metrics -------------------
# ---------------------------------------------------------------------------

####### Centrality Temporal ######

# Open file to save console output
sink("console_output_temporal_all.txt")

#graph <- graph_from_adjacency_matrix(cont)
qgraph_obj <- qgraph::qgraph(temp, DoNotPlot=TRUE)
plot (qgraph_obj)


center <- centrality(qgraph_obj)
center2 <- centrality_auto(qgraph_obj)
print(center2)


# Redirect the console output back to the console
sink()

#Plot Centrality
centralityPlot(qgraph_obj)
centralityTable(qgraph_obj)
```

```
igraph_obj <- igraph::graph_from_adjacency_matrix(cor(cont))
out_igraph <- expectedInf(igraph_obj)
qgraph_obj <- qgraph::qgraph(temp, DoNotPlot=TRUE)
out_qgraph <- expectedInf(qgraph_obj)

#Strength centrality
strength_centrality <- strength(igraph_obj, mode = "all")

#Show result
print(strength_centrality)

out_cont <- expectedInf(cont, step = c("both", 1, 2), directed = FALSE)
out_bet <- expectedInf(bet, step = c("both", 1, 2), directed = FALSE)
out_temp <- expectedInf(temp, step = c("both", 1, 2), directed = FALSE)

out_cont$step1
out_cont$step2

#expected influence plot for contemp network
plot(out_cont)
plot(out_cont, order="value", zscore=TRUE)

#expected influence plot for temporal network

plot(out_temp)
plot(out_temp, order="value", zscore=TRUE)



####### Centrality Contemporaneous ######

# Open file to save console output
sink("console_output_contemp_all.txt")

#graph <- graph_from_adjacency_matrix(cont)
qgraph_obj <- qgraph::qgraph(cont, DoNotPlot=TRUE)
plot (qgraph_obj)


center <- centrality(qgraph_obj)
center2 <- centrality_auto(qgraph_obj)
print(center2)


# Redirect the console output back to the console
sink()



# --------------------------------------------------------------------------
# ------------------------ Create correlation matrix ----------------------
# --------------------------------------------------------------------------

selected_vars <- c("PBAT1", "PBAT2", "PBAT3", "PBAT4", "PBAT15", "PBAT16")
data_selected <- data_detrended[, selected_vars]

# Remove rows with missing values (NA)
data_selected <- na.omit(data_selected)

# # Calculate the correlation matrix
correlation_matrix <- cor(data_selected)

# Show the correlation matrix
print(correlation_matrix)

# Define file name for the output file
output_file <- "cor_matrix.csv"

# Save correlation matrix as CSV file
write.csv(correlation_matrix, file = output_file, row.names = TRUE)


 -------------------------------------------------------------------------
# Bootstrapping -----------------------------------------------------------
# --------------------------------------------------------------------------
# Goal: Perform bootstrap across individuals
# calculate sampling variability in centrality estimates
# Instead of dropping 25% of sample like in panel-gvar,
# we here just bootstrap data of different IDs 1000 times
# and then recalculate centrality

# Function to do that
bootstrap_mlvar <- function(data,            # dataset
                            id_col,          # name of id column
                            n_iter = 1000,   # number of iterations
                            seed,            # set seed
                            pct_drop = FALSE,
                            n_pct_drop = NULL,
                            n_cores_boot,         # number of cores
                            ...){            # arguments to mlVAR

  set.seed(seed)
```

```
  # Sample IDs from id_col
  unique_ids <- unique(data[[id_col]])

  # Draw IDs for sampling
  l_ids <- list()
  if(!pct_drop){
    # sample IDs with replacement
    for(i in 1:n_iter){
      l_ids[[i]] <- sample(unique_ids, replace = TRUE)
      }

  }
  if(isTRUE(pct_drop)){
    # calculate number of individuals to drop
    num_ids_to_delete <- round(length(unique_ids) * (n_pct_drop/100))

    num_ids_to_sample <- length(unique_ids) - num_ids_to_delete

    # sample IDs with replacement
    for(i in 1:n_iter){
      l_ids[[i]] <- sample(unique_ids, size = num_ids_to_sample)
    }

  }

  # Setup parallelization
  future::plan(future::multisession, workers = n_cores_boot)

  l_out <- future.apply::future_lapply(1:n_iter, future.seed = TRUE,
                                       function(i){

      # Save output
      l_boot <- list()


      # Create a new dataframe based on the sampled IDs
      sampled_data <- data[data[[id_col]] %in% l_ids[[i]], ]

      # refit model
      fit <- mlVAR(data = sampled_data,
                   ...)

      # Calculate centrality values of interest
      # Temporal Network
      plot_temp <- plot(fit, "temporal")
      plot_cont <- plot(fit, "contemporaneous")
      l_boot$cent_temp <- centrality(plot_temp)
      l_boot$cent_cont <- centrality(plot_cont)
      # l_boot$sampled_data <- sampled_data

      return(l_boot)
    })

  # Explicitly stop parallelization
  future::plan(future::sequential)


  return(l_out)


}


boot_correlated <- bootstrap_mlvar(
                             data = data_detrended,
                             id_col = 'VID',
                             seed = 35037,
                             n_iter = 1000,
                             n_cores_boot = 50,
                             pct_drop = TRUE,
                             n_pct_drop = 20,
                             vars=varLabs,
                             idvar="VID",
                             dayvar="day",
                             beepvar="beep",
                             lags = 1,
                             temporal = "correlated",
                             contemporaneous = "correlated",
                             nCores = 1)


# 6604.569 sec elapsed

saveRDS(boot_correlated, here("output/bootstrap_correlated.RDS"))

# Analyze Bootstrap -------------------------------------------------------
bootstrap_correlated <- readRDS(here("output/bootstrap_correlated.RDS"))

# Function to obtain rank of centrality
rank_temp_centrality <- function(x) {
  rank_in_cont <- rank(x$cent_cont$InDegree)
```

```
  rank_out_cont <- rank(x$cent_cont$OutDegree)
  rank_in_temp <- rank(x$cent_temp$InDegree)
  rank_out_temp <- rank(x$cent_temp$OutDegree)
  return(c(rank_in_cont = rank_in_cont,
           rank_out_cont = rank_out_cont,
           rank_in_temp = rank_in_temp,
           rank_out_temp = rank_out_temp))
}

l_rank <- lapply(bootstrap_correlated, rank_centrality)

result_rank <- do.call(rbind, l_rank)

result_rank %>%
  as.data.frame() %>%
  pivot_longer(cols = everything(), names_to = "name") %>%
  separate(name, sep = "\\.", into = c("type", "item")) %>%
  group_by(type, item) %>%
  count(value)
```
