# Supplementary material for: Psychological flexibility and cognitive-affective processes in young adults’ daily lives
Source: Sci Rep. 2024 Apr 8;14:8182. doi: 10.1038/s41598-024-58598-3 (PMC11001944; doi:10.1038/s41598-024-58598-3)
Supplement: Supplementary file 2 — Supplementary Information 2. [file 41598_2024_58598_MOESM2_ESM.pdf]

Notebook of the POCEMAS study

In this notebook the steps for creating the datasheet are described. The steps are:

1. Merge the different sourcefiles from Ethica and Qualtrics into one file
2. Datacleaing of the data

Import

Import all the needed libraries for the analysis

In [1]:
```
import pandas as pd
import matplotlib.pyplot as plt
import seaborn as sns
import numpy as np
from datetime import datetime
import statistics
import warnings

#disable warnings
warnings.filterwarnings('ignore')
```

1) Merge different sourcefiles

Load all sourcefiles into dataframes.

In [2]:
```
df_morning = pd.read_csv('EMA-Monitoringmorning.csv',skiprows=lambda x: x in [1, 2])
df_day = pd.read_csv('EMA-Monitoring.csv',skiprows=lambda x: x in [1, 2])
df_intro = pd.read_csv('INTRO.csv',skiprows=lambda x: x in [1, 2],sep=',')
df_exit = pd.read_csv('EXIT.csv',skiprows=lambda x: x in [1, 2])
df_starting  = pd.read_csv("Waking_hours.csv",sep=';')
df_ethica_morning = pd.read_csv("survey_responses_morning.csv",sep=',')
df_ethica_day = pd.read_csv("survey_responses_day.csv",sep=',')
```

Merge Ethica datasets. Ethica has one dataset for the first session of the day and one for the rest.

In [3]:
```
df_ethica_all = pd.concat([df_ethica_morning, df_ethica_day], ignore_index=True)
```

Cut the String of the columns "Schedule Time" and "Response Time" so that in the next step they can be changed into the datetime format.

Note: Some entries have no entry for "Response time" which are listed in the output.

In [4]:
```
for index, row in df_ethica_all.iterrows():
    try:
        df_ethica_all.at[index,'Scheduled Time'] = row['Scheduled Time'][0:19]
        df_ethica_all.at[index,'Response Time'] = row['Response Time'][0:19]
    except:
        print(index)
        print( row['Scheduled Time'])
        print( row['Response Time'])
```

```
272
2022-10-22 09:33:08 CEST
nan
5899
2023-06-09 22:18:56 CEST
nan
6403
2022-11-28 15:23:03 CET
nan
8584
2023-04-03 19:13:25 CEST
nan
8585
2023-03-28 12:05:28 CEST
nan
8586
2023-04-01 16:58:52 CEST
nan
8587
2023-03-28 14:32:22 CEST
nan
8922
2023-03-05 20:31:42 CET
nan
8923
2023-03-05 18:53:49 CET
nan
```

Change the type of the column "Schedule Time" and "Response Time" from String to datetime.

In [5]:
```
df_ethica_all['Scheduled Time'] = pd.to_datetime(df_ethica_all['Scheduled Time'])
df_ethica_all['Response Time'] = pd.to_datetime(df_ethica_all['Response Time'])
```

Sort the dataframe based on "Name" and "Scheduled Time".

In [6]:
```
df_ethica_all = df_ethica_all.sort_values(by=['Name','Scheduled Time'])
```

Show the tail of the dataframe.

In [7]:
```
df_ethica_all.tail()
```

Out[7]:

	Name	Device ID	Scheduled Time	Issued Time	Response Time	Duration (seconds) from scheduled to completion time	Duration (seconds) from first response to completion time	Location
440	73623	5843B857A05545EE9C806ED1E88B27EF	2023-07-26 07:49:46	2023-07-26 07:49:46 CEST	2023-07-26 08:19:30	1784.248	Unknown	NaN
4385	73623	5843B857A05545EE9C806ED1E88B27EF	2023-07-26 10:52:05	2023-07-26 10:52:05 CEST	2023-07-26 10:59:01	416.027	Unknown	NaN
4387	73623	5843B857A05545EE9C806ED1E88B27EF	2023-07-26 15:06:23	2023-07-26 15:06:23 CEST	2023-07-26 16:50:31	6248.781	Unknown	NaN
4386	73623	5843B857A05545EE9C806ED1E88B27EF	2023-07-26 16:48:37	2023-07-26 16:48:37 CEST	2023-07-26 16:48:37	Blocked	Unknown	NaN
4388	73623	5843B857A05545EE9C806ED1E88B27EF	2023-07-26 19:26:24	2023-07-26 19:26:24 CEST	2023-07-26 19:30:53	269.269	Unknown	NaN

Reset the index of the dataframe.

In [8]:
```
df_ethica_all = df_ethica_all.reset_index(drop=True)
```

Drop all columns, which are not needed for the next steps.

In [9]:
```
df_ethica_all = df_ethica_all.drop(['Device ID', 'Issued Time',
       'Duration (seconds) from scheduled to completion time',
       'Duration (seconds) from first response to completion time',
       'Location'],axis=1)
```

Add the VID for each participant.

In [10]:
```
df_ethica_all.tail()
```

Out[10]:

	Name	Scheduled Time	Response Time
13121	73623	2023-07-26 07:49:46	2023-07-26 08:19:30
13122	73623	2023-07-26 10:52:05	2023-07-26 10:59:01
13123	73623	2023-07-26 15:06:23	2023-07-26 16:50:31
13124	73623	2023-07-26 16:48:37	2023-07-26 16:48:37
13125	73623	2023-07-26 19:26:24	2023-07-26 19:30:53

Look at the starting dataframe, which contains the Ethica ID and the Qualtrics ID to merge both datasets based on "Name" and "Label".

In [11]:
```
df_starting.head()
```

Out[11]:

	Name	Label	code	Um wie viel Uhr wachen Sie morgens in der Regel auf
0	59981	1	3	08:00
1	59986	2	5	10:00
2	59992	3	4	09:00
3	60060	9	4	09:00
4	60079	10	2	07:00

List all unique "Name" variables.

In [12]:
```
df_starting['Name'].unique()
```

Out[12]:
```
array([59981, 59986, 59992, 60060, 60079, 60195, 60199, 60252, 60256,
       60260, 60371, 60376, 60380, 60447, 60553, 60554, 60558, 60663,
       60667, 60670, 60879, 60880, 61246, 61249, 61250, 61251, 61344,
       61360, 61363, 61596, 61598, 61604, 61607, 61612, 61616, 61694,
       61696, 62351, 62354, 62357, 62358, 62363, 62366, 62563, 62566,
       62847, 62849, 62853, 62854, 62861, 62864, 62865, 62951, 63284,
       63287, 63298, 63302, 63501, 63502, 63527, 68177, 68178, 68209,
       68212, 68422, 68426, 68461, 68463, 68710, 68714, 68760, 68766,
       68937, 68986, 68991, 69674, 69676, 69806, 69813, 70198, 70200,
       70230, 70234, 70417, 70419, 70725, 70727, 70776, 70783, 71121,
       71125, 71175, 72065, 72069, 72262, 72290, 72291, 72401, 72403,
       72424, 72425, 72581, 72586, 72669, 72671, 72846, 72848, 72941,
       72943, 72965, 72967, 73170, 73196, 73198, 73351, 73353, 73377,
       73379, 73479, 73481, 73501, 73503, 73606, 73607, 73623],
      dtype=int64)
```

Add now the VID based on the "Name" variable.

In [13]:
```
df_ethica_all['VID'] = 0
for index, row in df_ethica_all.iterrows():
    try:
        df_ethica_all.at[index,'VID'] = df_starting[df_starting["Name"]==row.Name]['Label']
    except:
        print(index)
```

Show the first 15 entries of the updated dataframe.

In [14]:
```
df_ethica_all.head(15)
```

Out[14]:

	Name	Scheduled Time	Response Time	VID
0	59981	2022-09-14 09:55:28	2022-09-14 10:01:15	1
1	59981	2022-09-14 12:46:24	2022-09-14 12:54:14	1
2	59981	2022-09-14 15:10:30	2022-09-14 15:59:13	1
3	59981	2022-09-14 18:05:56	2022-09-14 18:49:16	1
4	59981	2022-09-14 21:33:08	2022-09-14 21:56:12	1
5	59981	2022-09-15 09:43:09	2022-09-15 11:07:56	1
6	59981	2022-09-15 11:54:33	2022-09-15 12:37:32	1
7	59981	2022-09-15 15:26:12	2022-09-15 17:02:26	1
8	59981	2022-09-15 18:22:57	2022-09-15 18:37:53	1
9	59981	2022-09-15 20:50:57	2022-09-15 20:54:30	1
10	59981	2022-09-16 09:19:13	2022-09-16 10:51:41	1
11	59981	2022-09-16 11:45:49	2022-09-16 12:32:20	1
12	59981	2022-09-16 14:32:32	2022-09-16 16:15:37	1
13	59981	2022-09-16 18:15:48	2022-09-16 19:12:54	1
14	59981	2022-09-16 21:43:08	2022-09-16 21:50:45	1

Add "Session-ID" for each participant:

- Session-ID 0 is the introduction session
- Session-ID [1-105] are the EMA sessions
- Session-ID 106 is the exit session

In [15]:
```
df_ethica_all['Session-ID'] = 0
counter = 1
temp_vid = 1
for index, row in df_ethica_all.iterrows():
    if row.VID != temp_vid:
        counter = 1
        temp_vid = row.VID
    df_ethica_all.at[index,'Session-ID'] = counter
    counter = counter +1
```

Show the last 15 entries of the updated dataframe.

In [16]:
```
df_ethica_all.tail(15)
```

Out[16]:

	Name	Scheduled Time	Response Time	VID	Session-ID
13111	73623	2023-07-24 07:04:36	2023-07-24 07:06:35	172	91
13112	73623	2023-07-24 11:26:44	2023-07-24 11:30:00	172	92
13113	73623	2023-07-24 13:55:16	2023-07-24 14:28:51	172	93
13114	73623	2023-07-24 18:10:13	2023-07-24 18:14:10	172	94
13115	73623	2023-07-24 19:44:19	2023-07-24 19:51:41	172	95
13116	73623	2023-07-25 08:28:48	2023-07-25 08:31:09	172	96
13117	73623	2023-07-25 11:15:07	2023-07-25 12:13:21	172	97
13118	73623	2023-07-25 14:47:39	2023-07-25 14:53:40	172	98
13119	73623	2023-07-25 18:07:12	2023-07-25 18:11:16	172	99
13120	73623	2023-07-25 20:33:44	2023-07-25 21:33:13	172	100
13121	73623	2023-07-26 07:49:46	2023-07-26 08:19:30	172	101
13122	73623	2023-07-26 10:52:05	2023-07-26 10:59:01	172	102
13123	73623	2023-07-26 15:06:23	2023-07-26 16:50:31	172	103
13124	73623	2023-07-26 16:48:37	2023-07-26 16:48:37	172	104
13125	73623	2023-07-26 19:26:24	2023-07-26 19:30:53	172	105

Look at the updated dataframe if there are Session-IDs which are > 105

In [17]:
```
df_ethica_all[df_ethica_all['Session-ID']>105]
```

Out[17]:

	Name	Scheduled Time	Response Time	VID	Session-ID
4515	62366	2022-11-22 22:34:29	2022-11-22 22:36:31	45	106
6721	68212	2023-03-15 20:44:40	2023-03-15 20:50:43	104	106

Two subjects have 106 so a deeper look into the data has to be done.

Look for VID 45. There were two prompts sended by ethica at the last day, so that the last prompt can be deleted.

In [18]:
```
df_ethica_all.loc[[4513,4514,4515,4516]]
```

Out[18]:

	Name	Scheduled Time	Response Time	VID	Session-ID
4513	62366	2022-11-22 17:59:09	2022-11-22 18:14:45	45	104
4514	62366	2022-11-22 21:25:28	2022-11-22 22:34:24	45	105
4515	62366	2022-11-22 22:34:29	2022-11-22 22:36:31	45	106
4516	62563	2022-11-04 08:25:24	2022-11-04 08:30:06	47	1

In [19]:
```
df_ethica_all = df_ethica_all.drop([4515])
```

In [20]:
```
df_ethica_all.loc[[4513,4514,4516]]
```

Out[20]:

	Name	Scheduled Time	Response Time	VID	Session-ID
4513	62366	2022-11-22 17:59:09	2022-11-22 18:14:45	45	104
4514	62366	2022-11-22 21:25:28	2022-11-22 22:34:24	45	105
4516	62563	2022-11-04 08:25:24	2022-11-04 08:30:06	47	1

Look for VID 104. There were two prompts at the last day, so that Session ID 104 will be deleted and the following has to be reindexed.

In [21]:
```
df_ethica_all.loc[[6717,6718,6719,6720,6721,6722]]
```

Out[21]:

	Name	Scheduled Time	Response Time	VID	Session-ID
6717	68212	2023-03-15 12:10:50	2023-03-15 14:10:50	104	102
6718	68212	2023-03-15 15:32:57	2023-03-15 17:08:05	104	103
6719	68212	2023-03-15 17:08:06	2023-03-15 17:09:33	104	104
6720	68212	2023-03-15 18:19:50	2023-03-15 18:26:56	104	105
6721	68212	2023-03-15 20:44:40	2023-03-15 20:50:43	104	106
6722	68422	2023-03-01 08:19:25	2023-03-01 09:51:18	111	1

Delete Session-ID 104 reindex the Session-ID of the following entries.

In [22]:
```
df_ethica_all = df_ethica_all.drop([6719])
df_ethica_all.at[6720,'Session-ID'] = 104
df_ethica_all.at[6721,'Session-ID'] = 105
```

In [23]:
```
df_ethica_all.loc[[6717,6718,6720,6721,6722]]
```

Out[23]:

	Name	Scheduled Time	Response Time	VID	Session-ID
6717	68212	2023-03-15 12:10:50	2023-03-15 14:10:50	104	102
6718	68212	2023-03-15 15:32:57	2023-03-15 17:08:05	104	103
6720	68212	2023-03-15 18:19:50	2023-03-15 18:26:56	104	104
6721	68212	2023-03-15 20:44:40	2023-03-15 20:50:43	104	105
6722	68422	2023-03-01 08:19:25	2023-03-01 09:51:18	111	1

Check again if any Session-IDs > 105 are available in the dataframe

In [24]:
```
df_ethica_all[df_ethica_all['Session-ID']>105]
```

Out[24]:

Name	Scheduled Time	Response Time	VID	Session-ID

Reset the indes of the dataframe after these steps

In [25]:
```
df_ethica_all = df_ethica_all.reset_index(drop=True)
```

Merge the different datasets

First of all to look what kind of column are available for each dataset.

Columns of EMA morning dataframe

'StartDate', 'EndDate', 'Status', 'IPAddress', 'Progress','Duration (in seconds)', 'Finished', 'RecordedDate', 'ResponseId', 'RecipientLastName', 'RecipientFirstName', 'RecipientEmail', 'ExternalReference', 'LocationLatitude', 'LocationLongitude','DistributionChannel', 'UserLanguage', 'VID', 'Sleep-Hour_1','Sleep-Quality _1', 'PBAT-1_1', 'PBAT-2 _2', 'PBAT-3_2', 'PBAT-4_2','PBAT-5_2', 'PBAT-6_2', 'PBAT-7_2', 'PBAT-8_2', 'PBAT-9_2', 'PBAT-10_2','PBAT-11_2', 'PBAT-12_2', 'PBAT-13_2', 'PBAT-14_2', 'PBAT-15_2','PBAT-16_2', 'PBAT-17_2', 'PBAT-18_2', 'Core-Affect-Valence_1','Core-Affect-Arousal_1', 'LifeSatisfaction_1', 'STOPD-1_1', 'STOPD-2_2','STOPD-3_2', 'STOPD-4_2', 'STOPD-5_2'

Columns of EMA day dataframe

'StartDate', 'EndDate', 'Status', 'IPAddress', 'Progress','Duration (in seconds)', 'Finished', 'RecordedDate', 'ResponseId', 'RecipientLastName', 'RecipientFirstName', 'RecipientEmail','ExternalReference', 'LocationLatitude', 'LocationLongitude', 'DistributionChannel', 'UserLanguage', 'VID', 'PBAT-1_1', 'PBAT-2_1', 'PBAT-3_1', 'PBAT-4_1', 'PBAT-5_1', 'PBAT-6_1', 'PBAT-7 _1', 'PBAT-8 _1', 'PBAT-9_1', 'PBAT-10_1', 'PBAT-11_1', 'PBAT-12_1', 'PBAT-13_1', 'PBAT-14_1', 'PBAT-15_1', 'PBAT-16_1', 'PBAT-17_1', 'PBAT-18_1', 'STOPD-1_1', 'STOPD-2_1', 'STOPD-3_1', 'STOPD-4_1', 'STOPD-5_1', 'Core-Affect-Arousal_1', 'Core-Affect-Valence_1', 'LifeSatisfaction_1'

Columns of Intro dataframe

'StartDate' , 'EndDate' , 'Status' , 'IPAddress' , 'Progress' , 'Duration (in seconds)' , 'Finished' , 'RecordedDate' , 'ResponseId' , 'RecipientLastName' , 'RecipientFirstName' , 'RecipientEmail' , 'ExternalReference' , 'LocationLatitude' , 'LocationLongitude' , 'DistributionChannel' , 'UserLanguage' , 'Einwilligung' , 'Criteria' , 'PBAT-1_1' , 'PBAT-2 _1' , 'PBAT-3 _1' , 'PBAT-4 _1' , 'PBAT-5 _1' , 'PBAT-6 _1' , 'PBAT-7 _1' , 'PBAT-8 _1' , 'PBAT-9 _1' , 'PBAT-10 _1' , 'PBAT-11 _1' , 'PBAT-12 _1' , 'PBAT-13_1' , 'PBAT-14 _1' , 'PBAT-15 _1' , 'PBAT-16 _1' , 'PBAT-17 _1' , 'PBAT-18 _1' , 'ExpCloseRelat_1' , 'ExpCloseRelat_2' , 'ExpCloseRelat_3' , 'ExpCloseRelat_4' , 'ExpCloseRelat_5' , 'ExpCloseRelat_6' , 'ExpCloseRelat_7' , 'ExpCloseRelat_8' , 'ExpCloseRelat_9' , 'ExpCloseRelat_10' , 'ExpCloseRelat_11' , 'ExpCloseRelat_12' , 'ExpCloseRelat_13' , 'ExpCloseRelat_14' , 'ExpCloseRelat_15' , 'ExpCloseRelat_16' , 'ExpCloseRelat_17' , 'ExpCloseRelat_18' , 'ExpCloseRelat_19' , 'ExpCloseRelat_20' , 'ExpCloseRelat_21' , 'ExpCloseRelat_22' , 'ExpCloseRelat_23' , 'ExpCloseRelat_24' , 'ExpCloseRelat_25' , 'ExpCloseRelat_26' , 'ExpCloseRelat_27' , 'ExpCloseRelat_28' , 'ExpCloseRelat_29' , 'ExpCloseRelat_30' , 'ExpCloseRelat_31' , 'ExpCloseRelat_32' , 'ExpCloseRelat_33' , 'ExpCloseRelat_34' , 'ExpCloseRelat_35' , 'ExpCloseRelat_36' , 'CovidImpactsQuesti_1' , 'CovidImpactsQuesti_2' , 'CovidImpactsQuesti_3' , 'CovidImpactsQuesti_4' , 'CovidImpactsQuesti_5' , 'CovidImpactsQuesti_6' , 'DASS_1' , 'DASS_2' , 'DASS_3' , 'DASS_4' , 'DASS_5' , 'DASS_6' , 'DASS_7' , 'DASS_8' , 'DASS_9' , 'DASS_10' , 'DASS_11' , 'DASS_12' , 'DASS_13' , 'DASS_14' , 'DASS_15' , 'DASS_16' , 'DASS_17' , 'DASS_18' , 'DASS_19' , 'DASS_20' , 'DASS_21' , 'AAQ_1' , 'AAQ_2' , 'AAQ_3' , 'AAQ_4' , 'AAQ_5' , 'AAQ_6' , 'AAQ_7' , 'Health-Survey' , 'CF_1' , 'CF_2' , 'CF_3' , 'CF_4' , 'CF_5' , 'CF_6' , 'CF_7' , 'BriefResilience_1' , 'BriefResilience_2' , 'BriefResilience_3' , 'BriefResilience_4' , 'BriefResilience_5' , 'BriefResilience_6' , 'SatisfactLife_1' , 'SatisfactLife_2' , 'SatisfactLife_3' , 'SatisfactLife_4' , 'SatisfactLife_5' , 'PercCovidThreat_1' , 'PercCovidThreat_2' , 'PercCovidThreat_3' , 'PercCovidThreat_4' , 'PercCovidThreat_5' , 'PercCovidThreat_6' , 'PANAS_1' , 'PANAS_2' , 'PANAS_3' , 'PANAS_4' , 'PANAS_5' , 'PANAS_6' , 'PANAS_7' , 'PANAS_8' , 'PANAS_9' , 'PANAS_10' , 'PANAS_11' , 'PANAS_12' , 'PANAS_13' , 'PANAS_14' , 'PANAS_15' , 'PANAS_16' , 'PANAS_17' , 'PANAS_18' , 'PANAS_19' , 'PANAS_20' , 'PSS_1' , 'PSS_2' , 'PSS_3' , 'PSS_4' , 'PSS_5' , 'PSS_6' , 'ASQ_1' , 'ASQ_2' , 'ASQ_3' , 'ASQ_4' , 'ASQ_5' , 'ASQ_6' , 'ASQ_7' , 'ASQ_8' , 'ASQ_9' , 'ASQ_10' , 'ASQ_11' , 'ASQ_12' , 'ASQ_13' , 'ASQ_14' , 'ASQ_15' , 'ASQ_16' , 'ASQ_17' , 'ASQ_18' , 'ASQ_19' , 'ASQ_20' , 'BPNSF_1' , 'BPNSF_2' , 'BPNSF_3' , 'BPNSF_4' , 'BPNSF_5' , 'BPNSF_6' , 'BPNSF_7' , 'BPNSF_8' , 'BPNSF_9' , 'BPNSF_10' , 'BPNSF_11' , 'BPNSF_12' , 'BPNSF_13' , 'BPNSF_14' , 'BPNSF_15' , 'BPNSF_16' , 'BPNSF_17' , 'BPNSF_18' , 'BPNSF_19' , 'BPNSF_20' , 'BPNSF_21' , 'BPNSF_22' , 'BPNSF_23' , 'BPNSF_24' , 'BriefSelfControl_1' , 'BriefSelfControl_2' , 'BriefSelfControl_3' , 'BriefSelfControl_4' , 'BriefSelfControl_5' , 'BriefSelfControl_6' , 'BriefSelfControl_7' , 'BriefSelfControl_8' , 'BriefSelfControl_9' , 'BriefSelfControl_10' , 'BriefSelfControl_11' , 'BriefSelfControl_12' , 'BriefSelfControl_13' , 'MAAS_1' , 'MAAS_2' , 'MAAS_3' , 'MAAS_4' , 'MAAS_5' , 'MAAS_6' , 'MAAS_7' , 'MAAS_8' , 'MAAS_9' , 'MAAS_10' , 'MAAS_11' , 'MAAS_12' , 'MAAS_13' , 'MAAS_14' , 'MAAS_15' , 'SFHS-1' , 'SFHS-2' , 'SFHS-3_1' , 'SFHS-3_2' , 'SFHS-3_3' , 'SFHS-3_4' , 'SFHS-3_5' , 'SFHS-3_6' , 'SFHS-3_7' , 'SFHS-3_8' , 'SFHS-3_9' , 'SFHS-3_10' , 'SFHS-4_1' , 'SFHS-4_2' , 'SFHS-4_3' , 'SFHS-4_4' , 'SFHS-5_1' , 'SFHS-5_2' , 'SFHS-5_3' , 'SFHS-6' , 'SFHS-7' , 'SFHS-8' , 'SFHS-9_1' , 'SFHS-9_2' , 'SFHS-9_3' , 'SFHS-9_4' , 'SFHS-9_5' , 'SFHS-9_6' , 'SFHS-9_7' , 'SFHS-9_8' , 'SFHS-9_9' , 'SFHS-10' , 'SFHS-11_1' , 'SFHS-11_2' , 'SFHS-11_3' , 'SFHS-11_4' , 'Gender' , 'Gender_3_TEXT' , 'Age' , 'Relationship' , 'Livingstatus' , 'Degree' , 'Occupation' , 'VID'

Columns of Exit dataframe

'StartDate' , 'EndDate' , 'Progress' , 'Duration (in seconds)' , 'Finished' , 'RecordedDate' , 'ResponseId' , 'AAQ_1' , 'AAQ_2' , 'AAQ_3' , 'AAQ_4' , 'AAQ_5' , 'AAQ_6' , 'AAQ_7' , 'ExpCloseRelat_1' , 'ExpCloseRelat_2' , 'ExpCloseRelat_3' , 'ExpCloseRelat_4' , 'ExpCloseRelat_5' , 'ExpCloseRelat_6' , 'ExpCloseRelat_7' , 'ExpCloseRelat_8' , 'ExpCloseRelat_9' , 'ExpCloseRelat_10' , 'ExpCloseRelat_11' , 'ExpCloseRelat_12' , 'ExpCloseRelat_13' , 'ExpCloseRelat_14' , 'ExpCloseRelat_15' , 'ExpCloseRelat_16' , 'ExpCloseRelat_17' , 'ExpCloseRelat_18' , 'ExpCloseRelat_19' , 'ExpCloseRelat_20' , 'ExpCloseRelat_21' , 'ExpCloseRelat_22' , 'ExpCloseRelat_23' , 'ExpCloseRelat_24' , 'ExpCloseRelat_25' , 'ExpCloseRelat_26' , 'ExpCloseRelat_27' , 'ExpCloseRelat_28' , 'ExpCloseRelat_29' , 'ExpCloseRelat_30' , 'ExpCloseRelat_31' , 'ExpCloseRelat_32' , 'ExpCloseRelat_33' , 'ExpCloseRelat_34' , 'ExpCloseRelat_35' , 'ExpCloseRelat_36' , 'SatisfactLife_1' , 'SatisfactLife_2' , 'SatisfactLife_3' , 'SatisfactLife_4' , 'SatisfactLife_5' , 'PercCovidThreat_1' , 'PercCovidThreat_2' , 'PercCovidThreat_3' , 'PercCovidThreat_4' , 'PercCovidThreat_5' , 'PercCovidThreat_6' , 'CovidImpactsQuesti_1' , 'CovidImpactsQuesti_2' , 'CovidImpactsQuesti_3' , 'CovidImpactsQuesti_4' , 'CovidImpactsQuesti_5' , 'CovidImpactsQuesti_6' , 'PSS_1' , 'PSS_2' , 'PSS_3' , 'PSS_4' , 'PSS_5' , 'PSS_6' , 'BriefResilience_1' , 'BriefResilience_2' , 'BriefResilience_3' , 'BriefResilience_4' , 'BriefResilience_5' , 'BriefResilience_6' , 'Health-Survey' , 'ASQ_1' , 'ASQ_2' , 'ASQ_3' , 'ASQ_4' , 'ASQ_5' , 'ASQ_6' , 'ASQ_7' , 'ASQ_8' , 'ASQ_9' , 'ASQ_10' , 'ASQ_11' , 'ASQ_12' , 'ASQ_13' , 'ASQ_14' , 'ASQ_15' , 'ASQ_16' , 'ASQ_17' , 'ASQ_18' , 'ASQ_19' , 'ASQ_20' , 'PANAS_1' , 'PANAS_2' , 'PANAS_3' , 'PANAS_4' , 'PANAS_5' , 'PANAS_6' , 'PANAS_7' , 'PANAS_8' , 'PANAS_9' , 'PANAS_10' , 'PANAS_11' , 'PANAS_12' , 'PANAS_13' , 'PANAS_14' , 'PANAS_15' , 'PANAS_16' , 'PANAS_17' , 'PANAS_18' , 'PANAS_19' , 'PANAS_20' , 'CF_1' , 'CF_2' , 'CF_3' , 'CF_4' , 'CF_5' , 'CF_6' , 'CF_7' , 'PBAT-1_18' , 'PBAT-2_17' , 'PBAT-3 _17' , 'PBAT-4 _17' , 'PBAT-5 _17' , 'PBAT-6 _17' , 'PBAT-7 _17' , 'PBAT-8 _17' , 'PBAT-9 _17' , 'PBAT-10 _17' , 'PBAT-11_17' , 'PBAT-12 _17' , 'PBAT-13 _17' , 'PBAT-14 _17' , 'PBAT-15 _17' , 'PBAT-16 _17' , 'PBAT-17 _17' , 'PBAT-18 _17' , 'DASS_1' , 'DASS_2' , 'DASS_3' , 'DASS_4' , 'DASS_5' , 'DASS_6' , 'DASS_7' , 'DASS_8' , 'DASS_9' , 'DASS_10' , 'DASS_11' , 'DASS_12' , 'DASS_13' , 'DASS_14' , 'DASS_15' , 'DASS_16' , 'DASS_17' , 'DASS_18' , 'DASS_19' , 'DASS_20' , 'DASS_21' , 'BSCS_1' , 'BSCS_2' , 'BSCS_3' , 'BSCS_4' , 'BSCS_5' , 'BSCS_6' , 'BSCS_7' , 'BSCS_8' , 'BSCS_9' , 'BSCS_10' , 'BSCS_11' , 'BSCS_12' , 'BSCS_13' , 'BPNSF_1' , 'BPNSF_2' , 'BPNSF_3' , 'BPNSF_4' , 'BPNSF_5' , 'BPNSF_6' , 'BPNSF_7' , 'BPNSF_8' , 'BPNSF_9' , 'BPNSF_10' , 'BPNSF_11' , 'BPNSF_12' , 'BPNSF_13' , 'BPNSF_14' , 'BPNSF_15' , 'BPNSF_16' , 'BPNSF_17' , 'BPNSF_18' , 'BPNSF_19' , 'BPNSF_20' , 'BPNSF_21' , 'BPNSF_22' , 'BPNSF_23' , 'BPNSF_24' , 'MAAS_1' , 'MAAS_2' , 'MAAS_3' , 'MAAS_4' , 'MAAS_5' , 'MAAS_6' , 'MAAS_7' , 'MAAS_8' , 'MAAS_9' , 'MAAS_10' , 'MAAS_11' , 'MAAS_12' , 'MAAS_13' , 'MAAS_14' , 'MAAS_15' , 'SFHS 1' , 'SFHS 2 ' , 'SFHS 3 _1' , 'SFHS 3 _2' , 'SFHS 3 _3' , 'SFHS 3 _4' , 'SFHS 3 _5' , 'SFHS 3 _6' , 'SFHS 3 _7' , 'SFHS 3 _8' , 'SFHS 3 _9' , 'SFHS 3 _10' , 'SFHS 4_1' , 'SFHS 4_2' , 'SFHS 4_3' , 'SFHS 4_4' , 'SFHS 5_1' , 'SFHS 5_2' , 'SFHS 5_3' , 'SFHS 6 ' , 'SFHS 7' , 'SFHS 8' , 'SFHS 9 _1' , 'SFHS 9 _2' , 'SFHS 9 _3' , 'SFHS 9 _4' , 'SFHS 9 _5' , 'SFHS 9 _6' , 'SFHS 9 _7' , 'SFHS 9 _8' , 'SFHS 9 _9' , 'SFHS 10' , 'SFHS 11 _1' , 'SFHS 11 _2' , 'SFHS 11 _3' , 'SFHS 11 _4' , 'Closure-2' , 'Closure-3_1' , 'Closure-3_2' , 'Closure-3_3' , 'Closure-3_4' , 'Closure-3_5' , 'Closure-3_6' , 'VID'

append the following colums to the dataframe

'Day', 'Session', 'weekday', 'LocationLatitude', 'LocationLongitude' ,'Gender' , 'Age' , 'Relationship' , 'Livingstatus' , 'Degree' , 'Occupation','StartDate' , 'EndDate' , 'Progress' , 'Duration (in seconds)' , 'Finished' , 'RecordedDate' , 'AAQ-1' , 'AAQ-2' , 'AAQ-3' , 'AAQ-4' , 'AAQ-5' , 'AAQ-6' , 'AAQ-7' , 'ExpCloseRelat-1' , 'ExpCloseRelat-2' , 'ExpCloseRelat-3' , 'ExpCloseRelat-4' , 'ExpCloseRelat-5' , 'ExpCloseRelat-6' , 'ExpCloseRelat-7' , 'ExpCloseRelat-8' , 'ExpCloseRelat-9' , 'ExpCloseRelat-10' , 'ExpCloseRelat-11' , 'ExpCloseRelat-12' , 'ExpCloseRelat-13' , 'ExpCloseRelat-14' , 'ExpCloseRelat-15' , 'ExpCloseRelat-16' , 'ExpCloseRelat-17' , 'ExpCloseRelat-18' , 'ExpCloseRelat-19' , 'ExpCloseRelat-20' , 'ExpCloseRelat-21' , 'ExpCloseRelat-22' , 'ExpCloseRelat-23' , 'ExpCloseRelat-24' , 'ExpCloseRelat-25' , 'ExpCloseRelat-26' , 'ExpCloseRelat-27' , 'ExpCloseRelat-28' , 'ExpCloseRelat-29' , 'ExpCloseRelat-30' , 'ExpCloseRelat-31' , 'ExpCloseRelat-32' , 'ExpCloseRelat-33' , 'ExpCloseRelat-34' , 'ExpCloseRelat-35' , 'ExpCloseRelat-36' , 'SatisfactLife-1' , 'SatisfactLife-2' , 'SatisfactLife-3' , 'SatisfactLife-4' , 'SatisfactLife-5' , 'PercCovidThreat-1' , 'PercCovidThreat-2' , 'PercCovidThreat-3' , 'PercCovidThreat-4' , 'PercCovidThreat-5' , 'PercCovidThreat-6' , 'CovidImpactsQuesti-1' , 'CovidImpactsQuesti-2' , 'CovidImpactsQuesti-3' , 'CovidImpactsQuesti-4' , 'CovidImpactsQuesti-5' , 'CovidImpactsQuesti-6' , 'PSS-1' , 'PSS-2' , 'PSS-3' , 'PSS-4' , 'PSS-5' , 'PSS-6' , 'BriefResilience-1' , 'BriefResilience-2' , 'BriefResilience-3' , 'BriefResilience-4' , 'BriefResilience-5' , 'BriefResilience-6' , 'Health-Survey' , 'ASQ-1' , 'ASQ-2' , 'ASQ-3' , 'ASQ-4' , 'ASQ-5' , 'ASQ-6' , 'ASQ-7' , 'ASQ-8' , 'ASQ-9' , 'ASQ-10' , 'ASQ-11' , 'ASQ-12' , 'ASQ-13' , 'ASQ-14' , 'ASQ-15' , 'ASQ-16' , 'ASQ-17' , 'ASQ-18' , 'ASQ-19' , 'ASQ-20' , 'PANAS-1' , 'PANAS-2' , 'PANAS-3' , 'PANAS-4' , 'PANAS-5' , 'PANAS-6' , 'PANAS-7' , 'PANAS-8' , 'PANAS-9' , 'PANAS-10' , 'PANAS-11' , 'PANAS-12' , 'PANAS-13' , 'PANAS-14' , 'PANAS-15' , 'PANAS-16' , 'PANAS-17' , 'PANAS-18' , 'PANAS-19' , 'PANAS-20' , 'CF-1' , 'CF-2' , 'CF-3' , 'CF-4' , 'CF-5' , 'CF-6' , 'CF-7' , 'DASS-1' , 'DASS-2' , 'DASS-3' , 'DASS-4' , 'DASS-5' , 'DASS-6' , 'DASS-7' , 'DASS-8' , 'DASS-9' , 'DASS-10' , 'DASS-11' , 'DASS-12' , 'DASS-13' , 'DASS-14' , 'DASS-15' , 'DASS-16' , 'DASS-17' , 'DASS-18' , 'DASS-19' , 'DASS-20' , 'DASS-21' , 'BSCS-1' , 'BSCS-2' , 'BSCS-3' , 'BSCS-4' , 'BSCS-5' , 'BSCS-6' , 'BSCS-7' , 'BSCS-8' , 'BSCS-9' , 'BSCS-10' , 'BSCS-11' , 'BSCS-12' , 'BSCS-13' , 'BPNSF-1' , 'BPNSF-2' , 'BPNSF-3' , 'BPNSF-4' , 'BPNSF-5' , 'BPNSF-6' , 'BPNSF-7' , 'BPNSF-8' , 'BPNSF-9' , 'BPNSF-10' , 'BPNSF-11' , 'BPNSF-12' , 'BPNSF-13' , 'BPNSF-14' , 'BPNSF-15' , 'BPNSF-16' , 'BPNSF-17' , 'BPNSF-18' , 'BPNSF-19' , 'BPNSF-20' , 'BPNSF-21' , 'BPNSF-22' , 'BPNSF-23' , 'BPNSF-24' , 'MAAS-1' , 'MAAS-2' , 'MAAS-3' , 'MAAS-4' , 'MAAS-5' , 'MAAS-6' , 'MAAS-7' , 'MAAS-8' , 'MAAS-9' , 'MAAS-10' , 'MAAS-11' , 'MAAS-12' , 'MAAS-13' , 'MAAS-14' , 'MAAS-15' , 'SFHS-1' , 'SFHS-2' , 'SFHS-3' , 'SFHS-4' , 'SFHS-5' , 'SFHS-6' , 'SFHS-7' , 'SFHS-8' , 'SFHS-9' , 'SFHS-10' , 'SFHS-11' , 'SFHS-12' , 'SFHS-13' , 'SFHS-14' , 'SFHS-15' , 'SFHS-16' , 'SFHS-17' , 'SFHS-18' , 'SFHS-19' , 'SFHS-20' , 'SFHS-21' , 'SFHS-22' , 'SFHS-23' , 'SFHS-24' , 'SFHS-25' , 'SFHS-26' , 'SFHS-27' , 'SFHS-28' , 'SFHS-29' , 'SFHS-30' , 'SFHS-31' , 'SFHS-32' , 'SFHS-33' , 'SFHS-34' , 'SFHS-35' , 'SFHS-36' , 'Closure-3-1' , 'Closure-3-2' , 'Closure-3-3' , 'Closure-3-4' , 'Closure-3-5' , 'Closure-3-6', 'PBAT-1' , 'PBAT-2' , 'PBAT-3' , 'PBAT-4' , 'PBAT-5' , 'PBAT-6' , 'PBAT-7' , 'PBAT-8' , 'PBAT-9' , 'PBAT-10' , 'PBAT-11' , 'PBAT-12' , 'PBAT-13' , 'PBAT-14' , 'PBAT-15' , 'PBAT-16' , 'PBAT-17' , 'PBAT-18' ,'Core-Affect-Valence','Core-Affect-Arousal','LifeSatisfaction', 'STOPD-1', 'STOPD-2', 'STOPD-3', 'STOPD-4', 'STOPD-5', 'Sleep-Hour','Sleep-Quality'

In [26]:
```
for i in ['Day', 'Session', 'weekday', 'LocationLatitude', 'LocationLongitude' ,'Gender' , 'Age' , 'Relationship' , 'Livingstatus' , 'Degree' , 'Occupation','StartDate'
    df_ethica_all[i] = -1
```

Check the updated dataframe by showing the first 15 rows.

In [27]:
```
df_ethica_all.head(15)
```

Out[27]:

	Name	Scheduled Time	Response Time	VID	Session-ID	Day	Session	weekday	LocationLatitude	LocationLongitude	...	Core-Affect-Valence	Core-Affect-Arousal	LifeSatisfaction	STOPD-1	STOPD-2	STOPD-3	STOPD-4	ST
0	59981	2022-09-14 09:55:28	2022-09-14 10:01:15	1	1	-1	-1	-1	-1	-1	...	-1	-1	-1	-1	-1	-1	-1	
1	59981	2022-09-14 12:46:24	2022-09-14 12:54:14	1	2	-1	-1	-1	-1	-1	...	-1	-1	-1	-1	-1	-1	-1	
2	59981	2022-09-14 15:10:30	2022-09-14 15:59:13	1	3	-1	-1	-1	-1	-1	...	-1	-1	-1	-1	-1	-1	-1	
3	59981	2022-09-14 18:05:56	2022-09-14 18:49:16	1	4	-1	-1	-1	-1	-1	...	-1	-1	-1	-1	-1	-1	-1	
4	59981	2022-09-14 21:33:08	2022-09-14 21:56:12	1	5	-1	-1	-1	-1	-1	...	-1	-1	-1	-1	-1	-1	-1	
5	59981	2022-09-15 09:43:09	2022-09-15 11:07:56	1	6	-1	-1	-1	-1	-1	...	-1	-1	-1	-1	-1	-1	-1	
6	59981	2022-09-15 11:54:33	2022-09-15 12:37:32	1	7	-1	-1	-1	-1	-1	...	-1	-1	-1	-1	-1	-1	-1	
7	59981	2022-09-15 15:26:12	2022-09-15 17:02:26	1	8	-1	-1	-1	-1	-1	...	-1	-1	-1	-1	-1	-1	-1	
8	59981	2022-09-15 18:22:57	2022-09-15 18:37:53	1	9	-1	-1	-1	-1	-1	...	-1	-1	-1	-1	-1	-1	-1	
9	59981	2022-09-15 20:50:57	2022-09-15 20:54:30	1	10	-1	-1	-1	-1	-1	...	-1	-1	-1	-1	-1	-1	-1	
10	59981	2022-09-16 09:19:13	2022-09-16 10:51:41	1	11	-1	-1	-1	-1	-1	...	-1	-1	-1	-1	-1	-1	-1	
11	59981	2022-09-16 11:45:49	2022-09-16 12:32:20	1	12	-1	-1	-1	-1	-1	...	-1	-1	-1	-1	-1	-1	-1	
12	59981	2022-09-16 14:32:32	2022-09-16 16:15:37	1	13	-1	-1	-1	-1	-1	...	-1	-1	-1	-1	-1	-1	-1	
13	59981	2022-09-16 18:15:48	2022-09-16 19:12:54	1	14	-1	-1	-1	-1	-1	...	-1	-1	-1	-1	-1	-1	-1	
14	59981	2022-09-16 21:43:08	2022-09-16 21:50:45	1	15	-1	-1	-1	-1	-1	...	-1	-1	-1	-1	-1	-1	-1	

15 rows × 285 columns

Prepare the intro dataframe for insertion into the main dataframe. Therefore remove all values which are not marked as finished.

In [28]:
```
df_intro.head()
```

Out[28]:

	StartDate	EndDate	Status	IPAddress	Progress	Duration (in seconds)	Finished	RecordedDate	ResponseId	RecipientLastName	...	SFHS-11_3	SFHS-11_4	Gender	Gender_3_TEXT	Age	Relatior
0	2022-09-13 09:47:48	2022-09-13 10:50:12	0	137.248.1.2	100	3744	1	2022-09-13 10:50:15	R_3O3yhe9lil1k6pB	NaN	...	3.0	2.0	2.0	NaN	22.0	
1	2022-09-13 11:50:04	2022-09-13 13:16:57	0	137.248.1.2	100	5212	1	2022-09-13 13:16:59	R_2TNnEC7V4Nnb5jy	NaN	...	5.0	2.0	1.0	NaN	21.0	
2	2022-09-13 14:00:56	2022-09-13 15:06:24	0	137.248.1.2	100	3927	1	2022-09-13 15:06:27	R_2Vg4VUB0DcQZnZy	NaN	...	4.0	4.0	1.0	NaN	21.0	
3	2022-09-07 16:13:02	2022-09-07 16:15:06	0	137.248.1.2	2	124	0	2022-09-14 16:15:09	R_5z2KaduRt5MT8B3	NaN	...	NaN	NaN	NaN	NaN	NaN	
4	2022-09-07 16:15:45	2022-09-07 16:16:55	8	137.248.1.2	2	70	0	2022-09-14 16:17:00	R_NVmfBJN6yv6Qunn	NaN	...	NaN	NaN	NaN	NaN	NaN	

5 rows × 274 columns

In [29]:
```
df_intro = df_intro[df_intro['Finished']==1]
df_intro['VID'].unique()
```

Out[29]:
```
array([  1.,   2.,   3.,   9.,  10.,   4.,   5.,   7.,   8.,  15.,  12.,
        11.,  16.,  13.,  18.,  17.,  19.,  20.,  23.,  24.,  21.,  22.,
        28.,  25.,  29.,  30.,  31.,  26.,  33.,  36.,  35.,  14.,  37.,
        39.,  38.,  34.,  40.,  32.,  27.,  42.,  43.,  45.,  44.,  46.,
        47.,  48.,  50.,  51.,  53.,  60.,  58.,  63.,  49.,  55.,  62.,
        61.,  59.,  54.,  57.,  64., 101., 102., 107., 104., 111., 105.,
       103., 110., 117., 113., 123., 134., 112., 133., 131., 126., 122.,
       116., 119., 130., 129., 108., 114., 128., 124., 120., 138., 132.,
       115., 135., 141., 137., 139., 125., 140., 146., 143., 148., 145.,
       127., 150., 147., 144., 151., 155., 142., 157., 162., 154., 152.,
       163., 171., 164., 161., 159.,  nan, 158., 166., 160., 168., 169.,
       149., 165., 153., 170., 172.])
```

Delete the entry containing the nan value and reindex the intro dataframe.

In [30]:
```
df_intro.loc[123]
```

Out[30]:
```
StartDate       2023-06-19 11:12:13
EndDate         2023-06-19 11:37:02
Status                            0
IPAddress               137.248.1.6
Progress                        100
                         ...
Relationship                    NaN
Livingstatus                    NaN
Degree                          NaN
Occupation                      NaN
VID                             NaN
Name: 123, Length: 274, dtype: object
```

In [31]:
```
df_intro.drop(123,inplace=True)
df_intro = df_intro.reset_index(drop=True)
```

Insert now the data from the intro dataframe into the main dataframe.

VID 9 has two entries for the introduction. The second entry is the exit of VID 9. Therefore the other Session-Id is used.

In [32]:
```
for index,row in df_intro.iterrows():
    if row['VID'] == 9 and row['StartDate'] == '2022-10-07 11:28:34':
        ses_id = 106
    else:
        ses_id = 0

    new_row = {'VID':row['VID'],
               'StartDate':row['StartDate'],
               'EndDate':row['EndDate'],
               'Progress':row['Progress'],
               'Duration (in seconds)':row['Duration (in seconds)'],
               'Finished':row['Finished'],
               'RecordedDate':row['RecordedDate'],
               'Gender':row['Gender'],
               'Age':row['Age'],
               'Relationship':row['Relationship'],
               'Livingstatus':row['Livingstatus'],
               'Degree':row['Degree'],
               'Occupation':row['Occupation'],
               'LocationLatitude':row['LocationLatitude'],
               'LocationLongitude':row['LocationLongitude'],
               'Session-ID' : ses_id,



               'PBAT-1':row['PBAT-1_1'],
               'PBAT-2':row['PBAT-2 _1'],
               'PBAT-3':row['PBAT-3 _1'],
               'PBAT-4':row['PBAT-4 _1'],
               'PBAT-5':row['PBAT-5 _1'],
               'PBAT-6':row['PBAT-6 _1'],
               'PBAT-7':row['PBAT-7 _1'],
               'PBAT-8':row['PBAT-8 _1'],
               'PBAT-9':row['PBAT-9 _1'],
               'PBAT-10':row['PBAT-10 _1'],
               'PBAT-11':row['PBAT-11 _1'],
               'PBAT-12':row['PBAT-12 _1'],
               'PBAT-13':row['PBAT-13_1'],
               'PBAT-14':row['PBAT-14 _1'],
               'PBAT-15':row['PBAT-15 _1'],
               'PBAT-16':row['PBAT-16 _1'],
               'PBAT-17':row['PBAT-17 _1'],
               'PBAT-18':row['PBAT-18 _1'],

               'ExpCloseRelat-1':row['ExpCloseRelat_1'],
               'ExpCloseRelat-2':row['ExpCloseRelat_2'],
               'ExpCloseRelat-3':row['ExpCloseRelat_3'],
               'ExpCloseRelat-4':row['ExpCloseRelat_4'],
               'ExpCloseRelat-5':row['ExpCloseRelat_5'],
               'ExpCloseRelat-6':row['ExpCloseRelat_6'],
               'ExpCloseRelat-7':row['ExpCloseRelat_7'],
               'ExpCloseRelat-8':row['ExpCloseRelat_8'],
               'ExpCloseRelat-9':row['ExpCloseRelat_9'],
               'ExpCloseRelat-10':row['ExpCloseRelat_10'],
               'ExpCloseRelat-11':row['ExpCloseRelat_11'],
               'ExpCloseRelat-12':row['ExpCloseRelat_12'],
               'ExpCloseRelat-13':row['ExpCloseRelat_13'],
               'ExpCloseRelat-14':row['ExpCloseRelat_14'],
               'ExpCloseRelat-15':row['ExpCloseRelat_15'],
               'ExpCloseRelat-16':row['ExpCloseRelat_16'],
               'ExpCloseRelat-17':row['ExpCloseRelat_17'],
               'ExpCloseRelat-18':row['ExpCloseRelat_18'],
               'ExpCloseRelat-19':row['ExpCloseRelat_19'],
               'ExpCloseRelat-20':row['ExpCloseRelat_20'],
               'ExpCloseRelat-21':row['ExpCloseRelat_21'],
               'ExpCloseRelat-22':row['ExpCloseRelat_22'],
               'ExpCloseRelat-23':row['ExpCloseRelat_23'],
               'ExpCloseRelat-24':row['ExpCloseRelat_24'],
               'ExpCloseRelat-25':row['ExpCloseRelat_25'],
               'ExpCloseRelat-26':row['ExpCloseRelat_26'],
               'ExpCloseRelat-27':row['ExpCloseRelat_27'],
               'ExpCloseRelat-28':row['ExpCloseRelat_28'],
               'ExpCloseRelat-29':row['ExpCloseRelat_29'],
               'ExpCloseRelat-30':row['ExpCloseRelat_30'],
               'ExpCloseRelat-31':row['ExpCloseRelat_31'],
               'ExpCloseRelat-32':row['ExpCloseRelat_32'],
               'ExpCloseRelat-33':row['ExpCloseRelat_33'],
               'ExpCloseRelat-34':row['ExpCloseRelat_34'],
               'ExpCloseRelat-35':row['ExpCloseRelat_35'],
               'ExpCloseRelat-36':row['ExpCloseRelat_36'],

               'CovidImpactsQuesti-1':row['CovidImpactsQuesti_1'],
               'CovidImpactsQuesti-2':row['CovidImpactsQuesti_2'],
               'CovidImpactsQuesti-3':row['CovidImpactsQuesti_3'],
               'CovidImpactsQuesti-4':row['CovidImpactsQuesti_4'],
               'CovidImpactsQuesti-5':row['CovidImpactsQuesti_5'],
               'CovidImpactsQuesti-6':row['CovidImpactsQuesti_6'],

               'DASS-1':row['DASS_1'],
               'DASS-2':row['DASS_2'],
               'DASS-3':row['DASS_3'],
               'DASS-4':row['DASS_4'],
               'DASS-5':row['DASS_5'],
               'DASS-6':row['DASS_6'],
               'DASS-7':row['DASS_7'],
               'DASS-8':row['DASS_8'],
               'DASS-9':row['DASS_9'],
               'DASS-10':row['DASS_10'],
               'DASS-11':row['DASS_11'],
               'DASS-12':row['DASS_12'],
               'DASS-13':row['DASS_13'],
               'DASS-14':row['DASS_14'],
               'DASS-15':row['DASS_15'],
               'DASS-16':row['DASS_16'],
               'DASS-17':row['DASS_17'],
               'DASS-18':row['DASS_18'],
               'DASS-19':row['DASS_19'],
               'DASS-20':row['DASS_20'],
               'DASS-21':row['DASS_21'],

               'AAQ-1':row['AAQ_1'],
               'AAQ-2':row['AAQ_2'],
               'AAQ-3':row['AAQ_3'],
               'AAQ-4':row['AAQ_4'],
               'AAQ-5':row['AAQ_5'],
               'AAQ-6':row['AAQ_6'],
               'AAQ-7':row['AAQ_7'],

               'Health-Survey':row['Health-Survey'],

               'CF-1':row['CF_1'],
               'CF-2':row['CF_2'],
               'CF-3':row['CF_3'],
               'CF-4':row['CF_4'],
               'CF-5':row['CF_5'],
               'CF-6':row['CF_6'],
               'CF-7':row['CF_7'],

               'BriefResilience-1':row['BriefResilience_1'],
               'BriefResilience-2':row['BriefResilience_2'],
               'BriefResilience-3':row['BriefResilience_3'],
               'BriefResilience-4':row['BriefResilience_4'],
               'BriefResilience-5':row['BriefResilience_5'],
               'BriefResilience-6':row['BriefResilience_6'],

               'SatisfactLife-1':row['SatisfactLife_1'],
               'SatisfactLife-2':row['SatisfactLife_2'],
               'SatisfactLife-3':row['SatisfactLife_3'],
               'SatisfactLife-4':row['SatisfactLife_4'],
               'SatisfactLife-5':row['SatisfactLife_5'],

               'PercCovidThreat-1':row['PercCovidThreat_1'],
               'PercCovidThreat-2':row['PercCovidThreat_2'],
               'PercCovidThreat-3':row['PercCovidThreat_3'],
               'PercCovidThreat-4':row['PercCovidThreat_4'],
               'PercCovidThreat-5':row['PercCovidThreat_5'],
               'PercCovidThreat-6':row['PercCovidThreat_6'],

               'PANAS-1':row['PANAS_1'],
               'PANAS-2':row['PANAS_2'],
               'PANAS-3':row['PANAS_3'],
               'PANAS-4':row['PANAS_4'],
               'PANAS-5':row['PANAS_5'],
               'PANAS-6':row['PANAS_6'],
               'PANAS-7':row['PANAS_7'],
               'PANAS-8':row['PANAS_8'],
               'PANAS-9':row['PANAS_9'],
               'PANAS-10':row['PANAS_10'],
               'PANAS-11':row['PANAS_11'],
               'PANAS-12':row['PANAS_12'],
               'PANAS-13':row['PANAS_13'],
               'PANAS-14':row['PANAS_14'],
               'PANAS-15':row['PANAS_15'],
               'PANAS-16':row['PANAS_16'],
               'PANAS-17':row['PANAS_17'],
               'PANAS-18':row['PANAS_18'],
               'PANAS-19':row['PANAS_19'],
               'PANAS-20':row['PANAS_20'],

               'PSS-1':row['PSS_1'],
               'PSS-2':row['PSS_2'],
               'PSS-3':row['PSS_3'],
               'PSS-4':row['PSS_4'],
               'PSS-5':row['PSS_5'],
               'PSS-6':row['PSS_6'],

               'ASQ-1':row['ASQ_1'],
               'ASQ-2':row['ASQ_2'],
               'ASQ-3':row['ASQ_3'],
               'ASQ-4':row['ASQ_4'],
               'ASQ-5':row['ASQ_5'],
               'ASQ-6':row['ASQ_6'],
               'ASQ-7':row['ASQ_7'],
               'ASQ-8':row['ASQ_8'],
               'ASQ-9':row['ASQ_9'],
               'ASQ-10':row['ASQ_10'],
               'ASQ-11':row['ASQ_11'],
               'ASQ-12':row['ASQ_12'],
               'ASQ-13':row['ASQ_13'],
               'ASQ-14':row['ASQ_14'],
               'ASQ-15':row['ASQ_15'],
               'ASQ-16':row['ASQ_16'],
               'ASQ-17':row['ASQ_17'],
               'ASQ-18':row['ASQ_18'],
               'ASQ-19':row['ASQ_19'],
               'ASQ-20':row['ASQ_20'],

               'BPNSF-1':row['BPNSF_1'],
               'BPNSF-2':row['BPNSF_2'],
               'BPNSF-3':row['BPNSF_3'],
               'BPNSF-4':row['BPNSF_4'],
               'BPNSF-5':row['BPNSF_5'],
               'BPNSF-6':row['BPNSF_6'],
               'BPNSF-7':row['BPNSF_7'],
               'BPNSF-8':row['BPNSF_8'],
               'BPNSF-9':row['BPNSF_9'],
               'BPNSF-10':row['BPNSF_10'],
               'BPNSF-11':row['BPNSF_11'],
               'BPNSF-12':row['BPNSF_12'],
               'BPNSF-13':row['BPNSF_13'],
               'BPNSF-14':row['BPNSF_14'],
               'BPNSF-15':row['BPNSF_15'],
               'BPNSF-16':row['BPNSF_16'],
               'BPNSF-17':row['BPNSF_17'],
               'BPNSF-18':row['BPNSF_18'],
               'BPNSF-19':row['BPNSF_19'],
               'BPNSF-20':row['BPNSF_20'],
               'BPNSF-21':row['BPNSF_21'],
               'BPNSF-22':row['BPNSF_22'],
               'BPNSF-23':row['BPNSF_23'],
               'BPNSF-24':row['BPNSF_24'],

                #BriefSelfControl_1
               'BSCS-1':row['BriefSelfControl_1'],
               'BSCS-2':row['BriefSelfControl_2'],
               'BSCS-3':row['BriefSelfControl_3'],
               'BSCS-4':row['BriefSelfControl_4'],
               'BSCS-5':row['BriefSelfControl_5'],
               'BSCS-6':row['BriefSelfControl_6'],
               'BSCS-7':row['BriefSelfControl_7'],
               'BSCS-8':row['BriefSelfControl_8'],
               'BSCS-9':row['BriefSelfControl_9'],
```

```
                'BSCS-10':row['BriefSelfControl_10'],
                'BSCS-11':row['BriefSelfControl_11'],
                'BSCS-12':row['BriefSelfControl_12'],
                'BSCS-13':row['BriefSelfControl_13'],

                'MAAS-1':row['MAAS_1'],
                'MAAS-2':row['MAAS_2'],
                'MAAS-3':row['MAAS_3'],
                'MAAS-4':row['MAAS_4'],
                'MAAS-5':row['MAAS_5'],
                'MAAS-6':row['MAAS_6'],
                'MAAS-7':row['MAAS_7'],
                'MAAS-8':row['MAAS_8'],
                'MAAS-9':row['MAAS_9'],
                'MAAS-10':row['MAAS_10'],
                'MAAS-11':row['MAAS_11'],
                'MAAS-12':row['MAAS_12'],
                'MAAS-13':row['MAAS_13'],
                'MAAS-14':row['MAAS_14'],
                'MAAS-15':row['MAAS_15'],

                'SFHS-1':row['SFHS-1'],
                'SFHS-2':row['SFHS-2'],
                'SFHS-3':row['SFHS-3_1'],
                'SFHS-4':row['SFHS-3_2'],
                'SFHS-5':row['SFHS-3_3'],
                'SFHS-6':row['SFHS-3_4'],
                'SFHS-7':row['SFHS-3_5'],
                'SFHS-8':row['SFHS-3_6'],
                'SFHS-9':row['SFHS-3_7'],
                'SFHS-10':row['SFHS-3_8'],
                'SFHS-11':row['SFHS-3_9'],
                'SFHS-12':row['SFHS-3_10'],
                'SFHS-13':row['SFHS-4_1'],
                'SFHS-14':row['SFHS-4_2'],
                'SFHS-15':row['SFHS-4_3'],
                'SFHS-16':row['SFHS-4_4'],
                'SFHS-17':row['SFHS-5_1'],
                'SFHS-18':row['SFHS-5_2'],
                'SFHS-19':row['SFHS-5_3'],
                'SFHS-20':row['SFHS-6'],
                'SFHS-21':row['SFHS-7'],
                'SFHS-22':row['SFHS-8'],
                'SFHS-23':row['SFHS-9_1'],
                'SFHS-24':row['SFHS-9_2'],
                'SFHS-25':row['SFHS-9_3'],
                'SFHS-26':row['SFHS-9_4'],
                'SFHS-27':row['SFHS-9_5'],
                'SFHS-28':row['SFHS-9_6'],
                'SFHS-29':row['SFHS-9_7'],
                'SFHS-30':row['SFHS-9_8'],
                'SFHS-31':row['SFHS-9_9'],
                'SFHS-32':row['SFHS-10'],
                'SFHS-33':row['SFHS-11_1'],
                'SFHS-34':row['SFHS-11_2'],
                'SFHS-35':row['SFHS-11_3'],
                'SFHS-36':row['SFHS-11_4'],

                }
    #append row to the dataframe
    df_ethica_all = pd.concat([df_ethica_all, pd.DataFrame([new_row])], ignore_index=True)
```

Prepare the exit dataframe for insertion into the main dataframe. Therefore remove all values which are not marked as finished.

In [33]:
```
df_exit = df_exit[df_exit['Finished']==1]
df_exit['VID'].unique()
```

Out[33]:
```
array([  2.,   1.,   3.,  10.,   4.,   5.,   8.,   7.,  15.,  12.,  16.,
        11.,  13.,  18.,  17.,  19.,  24.,  20.,  23.,  21.,  22.,  25.,
        30.,  28.,  29.,  31.,  33.,  26.,  14.,  39.,  35.,  37.,  38.,
        36.,  34.,  40.,  42.,  45.,  32.,  44.,  27.,  43.,  47.,  46.,
        48.,  53.,  60.,  58.,  51.,  50.,  49.,  63.,  61.,  55.,  59.,
        62.,  54.,  64.,  57., 101., 102., 104., 107., 105., 111., 103.,
       110., 117., 113., 123., 134., 112., 131., 133., 126., 122., 116.,
       119., 129., 130., 114., 108., 128., 124., 138., 120., 115., 132.,
       141., 135., 137., 139., 125., 140., 143., 146., 148., 150., 127.,
       145., 147., 144., 151., 155., 157., 142., 162., 154., 163., 152.,
       171., 164., 161., 159., 158., 166., 160., 168., 169., 165., 149.,
       153., 170., 172.])
```

In [34]:
```
for index,row in df_exit.iterrows():


    new_row = {'VID':row['VID'],
                'StartDate':row['StartDate'],
                'EndDate':row['EndDate'],
                'Progress':row['Progress'],
                'Duration (in seconds)':row['Duration (in seconds)'],
                'Finished':row['Finished'],
                'RecordedDate':row['RecordedDate'],
                'LocationLatitude':row['LocationLatitude'],
                'LocationLongitude':row['LocationLongitude'],
                'Session-ID' : 106,

                'AAQ-1':row['AAQ_1'],
                'AAQ-2':row['AAQ_2'],
                'AAQ-3':row['AAQ_3'],
                'AAQ-4':row['AAQ_4'],
                'AAQ-5':row['AAQ_5'],
                'AAQ-6':row['AAQ_6'],
                'AAQ-7':row['AAQ_7'],

                'ExpCloseRelat-1':row['ExpCloseRelat_1'],
                'ExpCloseRelat-2':row['ExpCloseRelat_2'],
                'ExpCloseRelat-3':row['ExpCloseRelat_3'],
                'ExpCloseRelat-4':row['ExpCloseRelat_4'],
                'ExpCloseRelat-5':row['ExpCloseRelat_5'],
                'ExpCloseRelat-6':row['ExpCloseRelat_6'],
                'ExpCloseRelat-7':row['ExpCloseRelat_7'],
                'ExpCloseRelat-8':row['ExpCloseRelat_8'],
                'ExpCloseRelat-9':row['ExpCloseRelat_9'],
                'ExpCloseRelat-10':row['ExpCloseRelat_10'],
                'ExpCloseRelat-11':row['ExpCloseRelat_11'],
                'ExpCloseRelat-12':row['ExpCloseRelat_12'],
                'ExpCloseRelat-13':row['ExpCloseRelat_13'],
                'ExpCloseRelat-14':row['ExpCloseRelat_14'],
                'ExpCloseRelat-15':row['ExpCloseRelat_15'],
                'ExpCloseRelat-16':row['ExpCloseRelat_16'],
                'ExpCloseRelat-17':row['ExpCloseRelat_17'],
                'ExpCloseRelat-18':row['ExpCloseRelat_18'],
                'ExpCloseRelat-19':row['ExpCloseRelat_19'],
                'ExpCloseRelat-20':row['ExpCloseRelat_20'],
                'ExpCloseRelat-21':row['ExpCloseRelat_21'],
                'ExpCloseRelat-22':row['ExpCloseRelat_22'],
                'ExpCloseRelat-23':row['ExpCloseRelat_23'],
                'ExpCloseRelat-24':row['ExpCloseRelat_24'],
                'ExpCloseRelat-25':row['ExpCloseRelat_25'],
                'ExpCloseRelat-26':row['ExpCloseRelat_26'],
                'ExpCloseRelat-27':row['ExpCloseRelat_27'],
                'ExpCloseRelat-28':row['ExpCloseRelat_28'],
                'ExpCloseRelat-29':row['ExpCloseRelat_29'],
                'ExpCloseRelat-30':row['ExpCloseRelat_30'],
                'ExpCloseRelat-31':row['ExpCloseRelat_31'],
                'ExpCloseRelat-32':row['ExpCloseRelat_32'],
                'ExpCloseRelat-33':row['ExpCloseRelat_33'],
                'ExpCloseRelat-34':row['ExpCloseRelat_34'],
                'ExpCloseRelat-35':row['ExpCloseRelat_35'],
                'ExpCloseRelat-36':row['ExpCloseRelat_36'],

                'SatisfactLife-1':row['SatisfactLife_1'],
                'SatisfactLife-2':row['SatisfactLife_2'],
                'SatisfactLife-3':row['SatisfactLife_3'],
                'SatisfactLife-4':row['SatisfactLife_4'],
                'SatisfactLife-5':row['SatisfactLife_5'],

                'PercCovidThreat-1':row['PercCovidThreat_1'],
                'PercCovidThreat-2':row['PercCovidThreat_2'],
                'PercCovidThreat-3':row['PercCovidThreat_3'],
                'PercCovidThreat-4':row['PercCovidThreat_4'],
                'PercCovidThreat-5':row['PercCovidThreat_5'],
                'PercCovidThreat-6':row['PercCovidThreat_6'],

                'CovidImpactsQuesti-1':row['CovidImpactsQuesti_1'],
                'CovidImpactsQuesti-2':row['CovidImpactsQuesti_2'],
                'CovidImpactsQuesti-3':row['CovidImpactsQuesti_3'],
                'CovidImpactsQuesti-4':row['CovidImpactsQuesti_4'],
                'CovidImpactsQuesti-5':row['CovidImpactsQuesti_5'],
                'CovidImpactsQuesti-6':row['CovidImpactsQuesti_6'],

                'PSS-1':row['PSS_1'],
                'PSS-2':row['PSS_2'],
                'PSS-3':row['PSS_3'],
                'PSS-4':row['PSS_4'],
                'PSS-5':row['PSS_5'],
                'PSS-6':row['PSS_6'],

                'BriefResilience-1':row['BriefResilience_1'],
                'BriefResilience-2':row['BriefResilience_2'],
                'BriefResilience-3':row['BriefResilience_3'],
                'BriefResilience-4':row['BriefResilience_4'],
                'BriefResilience-5':row['BriefResilience_5'],
                'BriefResilience-6':row['BriefResilience_6'],

                'Health-Survey':row['Health-Survey'],

                'ASQ-1':row['ASQ_1'],
                'ASQ-2':row['ASQ_2'],
                'ASQ-3':row['ASQ_3'],
                'ASQ-4':row['ASQ_4'],
                'ASQ-5':row['ASQ_5'],
                'ASQ-6':row['ASQ_6'],
                'ASQ-7':row['ASQ_7'],
                'ASQ-8':row['ASQ_8'],
                'ASQ-9':row['ASQ_9'],
                'ASQ-10':row['ASQ_10'],
                'ASQ-11':row['ASQ_11'],
                'ASQ-12':row['ASQ_12'],
                'ASQ-13':row['ASQ_13'],
                'ASQ-14':row['ASQ_14'],
                'ASQ-15':row['ASQ_15'],
                'ASQ-16':row['ASQ_16'],
                'ASQ-17':row['ASQ_17'],
                'ASQ-18':row['ASQ_18'],
                'ASQ-19':row['ASQ_19'],
                'ASQ-20':row['ASQ_20'],

                'PANAS-1':row['PANAS_1'],
                'PANAS-2':row['PANAS_2'],
                'PANAS-3':row['PANAS_3'],
                'PANAS-4':row['PANAS_4'],
                'PANAS-5':row['PANAS_5'],
                'PANAS-6':row['PANAS_6'],
                'PANAS-7':row['PANAS_7'],
                'PANAS-8':row['PANAS_8'],
                'PANAS-9':row['PANAS_9'],
                'PANAS-10':row['PANAS_10'],
                'PANAS-11':row['PANAS_11'],
                'PANAS-12':row['PANAS_12'],
                'PANAS-13':row['PANAS_13'],
                'PANAS-14':row['PANAS_14'],
                'PANAS-15':row['PANAS_15'],
                'PANAS-16':row['PANAS_16'],
                'PANAS-17':row['PANAS_17'],
                'PANAS-18':row['PANAS_18'],
                'PANAS-19':row['PANAS_19'],
                'PANAS-20':row['PANAS_20'],

                'CF-1':row['CF_1'],
                'CF-2':row['CF_2'],
                'CF-3':row['CF_3'],
                'CF-4':row['CF_4'],
                'CF-5':row['CF_5'],
                'CF-6':row['CF_6'],
                'CF-7':row['CF_7'],

                'PBAT-1':row['PBAT-1_18'],
                'PBAT-2':row['PBAT-2_17'],
                'PBAT-3':row['PBAT-3 _17'],
                'PBAT-4':row['PBAT-4 _17'],
                'PBAT-5':row['PBAT-5 _17'],
                'PBAT-6':row['PBAT-6 _17'],
                'PBAT-7':row['PBAT-7 _17'],
                'PBAT-8':row['PBAT-8 _17'],
                'PBAT-9':row['PBAT-9 _17'],
                'PBAT-10':row['PBAT-10 _17'],
                'PBAT-11':row['PBAT-11_17'],
                'PBAT-12':row['PBAT-12 _17'],
                'PBAT-13':row['PBAT-13 _17'],
                'PBAT-14':row['PBAT-14 _17'],
                'PBAT-15':row['PBAT-15 _17'],
                'PBAT-16':row['PBAT-16 _17'],
                'PBAT-17':row['PBAT-17 _17'],
                'PBAT-18':row['PBAT-18 _17'],

                'DASS-1':row['DASS_1'],
                'DASS-2':row['DASS_2'],
                'DASS-3':row['DASS_3'],
                'DASS-4':row['DASS_4'],
                'DASS-5':row['DASS_5'],
                'DASS-6':row['DASS_6'],
                'DASS-7':row['DASS_7'],
                'DASS-8':row['DASS_8'],
                'DASS-9':row['DASS_9'],
                'DASS-10':row['DASS_10'],
                'DASS-11':row['DASS_11'],
                'DASS-12':row['DASS_12'],
                'DASS-13':row['DASS_13'],
                'DASS-14':row['DASS_14'],
                'DASS-15':row['DASS_15'],
                'DASS-16':row['DASS_16'],
                'DASS-17':row['DASS_17'],
                'DASS-18':row['DASS_18'],
                'DASS-19':row['DASS_19'],
                'DASS-20':row['DASS_20'],
                'DASS-21':row['DASS_21'],

                'BSCS-1':row['BSCS_1'],
                'BSCS-2':row['BSCS_2'],
                'BSCS-3':row['BSCS_3'],
                'BSCS-4':row['BSCS_4'],
                'BSCS-5':row['BSCS_5'],
                'BSCS-6':row['BSCS_6'],
                'BSCS-7':row['BSCS_7'],
                'BSCS-8':row['BSCS_8'],
                'BSCS-9':row['BSCS_9'],
                'BSCS-10':row['BSCS_10'],
                'BSCS-11':row['BSCS_11'],
                'BSCS-12':row['BSCS_12'],
                'BSCS-13':row['BSCS_13'],

                'BPNSF-1':row['BPNSF_1'],
                'BPNSF-2':row['BPNSF_2'],
                'BPNSF-3':row['BPNSF_3'],
                'BPNSF-4':row['BPNSF_4'],
                'BPNSF-5':row['BPNSF_5'],
                'BPNSF-6':row['BPNSF_6'],
                'BPNSF-7':row['BPNSF_7'],
                'BPNSF-8':row['BPNSF_8'],
                'BPNSF-9':row['BPNSF_9'],
                'BPNSF-10':row['BPNSF_10'],
                'BPNSF-11':row['BPNSF_11'],
                'BPNSF-12':row['BPNSF_12'],
                'BPNSF-13':row['BPNSF_13'],
                'BPNSF-14':row['BPNSF_14'],
                'BPNSF-15':row['BPNSF_15'],
                'BPNSF-16':row['BPNSF_16'],
                'BPNSF-17':row['BPNSF_17'],
                'BPNSF-18':row['BPNSF_18'],
                'BPNSF-19':row['BPNSF_19'],
                'BPNSF-20':row['BPNSF_20'],
                'BPNSF-21':row['BPNSF_21'],
                'BPNSF-22':row['BPNSF_22'],
                'BPNSF-23':row['BPNSF_23'],
                'BPNSF-24':row['BPNSF_24'],

                'MAAS-1':row['MAAS_1'],
                'MAAS-2':row['MAAS_2'],
                'MAAS-3':row['MAAS_3'],
                'MAAS-4':row['MAAS_4'],
                'MAAS-5':row['MAAS_5'],
                'MAAS-6':row['MAAS_6'],
                'MAAS-7':row['MAAS_7'],
                'MAAS-8':row['MAAS_8'],
                'MAAS-9':row['MAAS_9'],
                'MAAS-10':row['MAAS_10'],
                'MAAS-11':row['MAAS_11'],
                'MAAS-12':row['MAAS_12'],
                'MAAS-13':row['MAAS_13'],
                'MAAS-14':row['MAAS_14'],
                'MAAS-15':row['MAAS_15'],

                'SFHS-1':row['SFHS 1'],
                'SFHS-2':row['SFHS 2 '],
                'SFHS-3':row['SFHS 3 _1'],
                'SFHS-4':row['SFHS 3 _2'],
                'SFHS-5':row['SFHS 3 _3'],
                'SFHS-6':row['SFHS 3 _4'],
                'SFHS-7':row['SFHS 3 _5'],
                'SFHS-8':row['SFHS 3 _6'],
                'SFHS-9':row['SFHS 3 _7'],
                'SFHS-10':row['SFHS 3 _8'],
                'SFHS-11':row['SFHS 3 _9'],
                'SFHS-12':row['SFHS 3 _10'],
                'SFHS-13':row['SFHS 4_1'],
                'SFHS-14':row['SFHS 4_2'],
                'SFHS-15':row['SFHS 4_3'],
                'SFHS-16':row['SFHS 4_4'],
                'SFHS-17':row['SFHS 5_1'],
                'SFHS-18':row['SFHS 5_2'],
                'SFHS-19':row['SFHS 5_3'],
                'SFHS-20':row['SFHS 6 '],
                'SFHS-21':row['SFHS 7'],
                'SFHS-22':row['SFHS 8'],
                'SFHS-23':row['SFHS 9 _1'],
                'SFHS-24':row['SFHS 9 _2'],
                'SFHS-25':row['SFHS 9 _3'],
                'SFHS-26':row['SFHS 9 _4'],
                'SFHS-27':row['SFHS 9 _5'],
                'SFHS-28':row['SFHS 9 _6'],
                'SFHS-29':row['SFHS 9 _7'],
                'SFHS-30':row['SFHS 9 _8'],
                'SFHS-31':row['SFHS 9 _9'],
                'SFHS-32':row['SFHS 10'],
                'SFHS-33':row['SFHS 11 _1'],
                'SFHS-34':row['SFHS 11 _2'],
                'SFHS-35':row['SFHS 11 _3'],
                'SFHS-36':row['SFHS 11 _4'],

                'Closure-3-1':row['Closure-3_1'],
                'Closure-3-2':row['Closure-3_2'],
                'Closure-3-3':row['Closure-3_3'],
                'Closure-3-4':row['Closure-3_4'],
                'Closure-3-5':row['Closure-3_5'],
                'Closure-3-6':row['Closure-3_6'],

                }
    #append row to the dataframe
    df_ethica_all = pd.concat([df_ethica_all, pd.DataFrame([new_row])], ignore_index=True)
```

Now change the column "weekday" to String and "Schedule Time" and "Response Time" to datetime

In [35]:
```
df_ethica_all['weekday'] = df_ethica_all['weekday'].astype(str)
df_ethica_all['Scheduled Time'] = pd.to_datetime(df_ethica_all['Scheduled Time'])
df_ethica_all['Response Time'] = pd.to_datetime(df_ethica_all['Response Time'])
```

Prepare and insert EMA morning dataframe. First remove all values which are not marked as finished and drop VID 777.

Show the first 5 entries of the EMA morning dataframe.

In [36]:
```
df_morning.head()
```

Out[36]:

	StartDate	EndDate	Status	IPAddress	Progress	Duration (in seconds)	Finished	RecordedDate	ResponseId	RecipientLastName	...	PBAT-17_2	PBAT-18_2	Core-Affect-Valence_1	Core-Affect-Arousal_1	LifeSatisfactio
0	2022-09-13 10:07:45	2022-09-13 10:07:52	0	137.248.79.198	3	6	0	2022-09-13 11:07:57	R_2UflicaDMETwgBO	NaN	...	NaN	NaN	NaN	NaN	
1	2022-09-13 11:11:59	2022-09-13 11:12:04	0	137.248.4.248	3	5	0	2022-09-13 12:22:12	R_1dbMooyGsPnCwjs	NaN	...	NaN	NaN	NaN	NaN	
2	2022-09-14 09:33:46	2022-09-14 09:36:42	0	178.203.144.72	100	175	1	2022-09-14 09:36:42	R_22Q6I8xNVSdapdJ	NaN	...	58.0	59.0	80.0	29.0	
3	2022-09-14 09:58:55	2022-09-14 10:01:09	0	178.203.146.227	100	134	1	2022-09-14 10:01:10	R_8Hcv7IXg1IqeGwF	NaN	...	60.0	61.0	92.0	71.0	
4	2022-09-14 13:07:44	2022-09-14 13:12:04	0	84.134.81.182	100	259	1	2022-09-14 13:12:06	R_Zn6q7u5CL4vmT4J	NaN	...	78.0	78.0	66.0	11.0	

5 rows × 46 columns

In [37]:
```
df_morning = df_morning[df_morning['Finished']==1]
df_morning = df_morning.drop(df_morning[df_morning['VID']==777].index)
```

Set "StartDate" as datetime, then sort the values by "VID" and "StartDate" and reset the index.

In [38]:
```
df_morning['StartDate'] = pd.to_datetime(df_morning['StartDate'])
df_morning = df_morning.sort_values(by=['VID','StartDate'])
df_morning = df_morning.reset_index(drop=True)
```

Drop index 120, because VID 7 is not reidentifiable.

In [39]:
```
df_morning = df_morning.drop(120)
df_morning = df_morning.reset_index(drop=True)
```

Find the morning sessions baded on the Session-ID (using modulo 5 with rest = 1) and insert the data.

In [40]:
```
morning_index = []

for index,row in df_ethica_all.iterrows():
    if row["Session-ID"]%5==1 and row["Session-ID"] < 106:
        temp_vid = row['VID']
        day_ethica = pd.Timestamp(row['Scheduled Time'].year, row['Scheduled Time'].month, row['Scheduled Time'].day)
        for index2,row2 in df_morning[df_morning['VID']==temp_vid].iterrows():
            day_morining = pd.Timestamp(row2.StartDate.year, row2.StartDate.month, row2.StartDate.day)
            if (row2['VID'] == temp_vid) and (day_ethica == day_morining):

                df_ethica_all.at[index, "weekday"] = row2["StartDate"].day_name()
                df_ethica_all.at[index, "StartDate"] = row2["StartDate"]
                df_ethica_all.at[index, "EndDate"] = row2["EndDate"]
                df_ethica_all.at[index, "Progress"] = row2["Progress"]
                df_ethica_all.at[index, "Duration (in seconds)"] = row2["Duration (in seconds)"]
                df_ethica_all.at[index, "Finished"] = row2["Finished"]
                df_ethica_all.at[index, "LocationLatitude"] = row2["LocationLatitude"]
                df_ethica_all.at[index, "LocationLongitude"] = row2["LocationLongitude"]
                df_ethica_all.at[index, "Session"] = 1

                df_ethica_all.at[index, "Sleep-Hour"] = row2["Sleep-Hour_1"]
                df_ethica_all.at[index, "Sleep-Quality"] = row2["Sleep-Quality _1"]


                df_ethica_all.at[index, "PBAT-1"] = row2["PBAT-1_1"]
                df_ethica_all.at[index, "PBAT-2"] = row2["PBAT-2 _2"]
                df_ethica_all.at[index, "PBAT-3"] = row2["PBAT-3_2"]
                df_ethica_all.at[index, "PBAT-4"] = row2["PBAT-4_2"]
                df_ethica_all.at[index, "PBAT-5"] = row2["PBAT-5_2"]
                df_ethica_all.at[index, "PBAT-6"] = row2["PBAT-6_2"]
                df_ethica_all.at[index, "PBAT-7"] = row2["PBAT-7_2"]
                df_ethica_all.at[index, "PBAT-8"] = row2["PBAT-8_2"]
                df_ethica_all.at[index, "PBAT-9"] = row2["PBAT-9_2"]
                df_ethica_all.at[index, "PBAT-10"] = row2["PBAT-10_2"]
                df_ethica_all.at[index, "PBAT-11"] = row2["PBAT-11_2"]
                df_ethica_all.at[index, "PBAT-12"] = row2["PBAT-12_2"]
                df_ethica_all.at[index, "PBAT-13"] = row2["PBAT-13_2"]
                df_ethica_all.at[index, "PBAT-14"] = row2["PBAT-14_2"]
                df_ethica_all.at[index, "PBAT-15"] = row2["PBAT-15_2"]
                df_ethica_all.at[index, "PBAT-16"] = row2["PBAT-16_2"]
                df_ethica_all.at[index, "PBAT-17"] = row2["PBAT-17_2"]
                df_ethica_all.at[index, "PBAT-18"] = row2["PBAT-18_2"]

                df_ethica_all.at[index, "Core-Affect-Valence"] = row2["Core-Affect-Valence_1"]
                df_ethica_all.at[index, "Core-Affect-Arousal"] = row2["Core-Affect-Arousal_1"]

                df_ethica_all.at[index, "LifeSatisfaction"] = row2["LifeSatisfaction_1"]

                df_ethica_all.at[index, "STOPD-1"] = row2["STOPD-1_1"]
                df_ethica_all.at[index, "STOPD-2"] = row2["STOPD-2_2"]
                df_ethica_all.at[index, "STOPD-3"] = row2["STOPD-3_2"]
                df_ethica_all.at[index, "STOPD-4"] = row2["STOPD-4_2"]
                df_ethica_all.at[index, "STOPD-5"] = row2["STOPD-5_2"]

                morning_index.append(index2)
                break
```

Create a file where all not inserted entries were listed which then will be checked by one author.

In [41]:
```
df_morning_drops = pd.read_csv('EMA-Monitoringmorning.csv',skiprows=lambda x: x in [1, 2])
df_morning_drops = df_morning_drops[df_morning_drops['Finished']==1]
df_morning_drops = df_morning_drops.drop(df_morning_drops[df_morning_drops['VID']==777].index)

df_morning_drops['StartDate'] = pd.to_datetime(df_morning_drops['StartDate'])
df_morning_drops = df_morning_drops.sort_values(by=['VID','StartDate'])
df_morning_drops = df_morning_drops.reset_index(drop=True)
```

In [42]:
```
df_morning_drops = df_morning_drops.drop(morning_index)
```

In [43]:
```
df_morning_drops.to_csv("EMA-morning-not-insert.csv")
```

Prepare and insert EMA day dataframe. Therefore remove all values which are not marked as finished.

Show the first 5 entries of the dataframe.

In [44]:
```
df_day.head()
```

Out[44]:

	StartDate	EndDate	Status	IPAddress	Progress	Duration (in seconds)	Finished	RecordedDate	ResponseId	RecipientLastName	...	PBAT-17_1	PBAT-18_1	STOPD-1_1	STOPD-2_1	STOPD-3_1	STOPD-4_
0	2022-09-14 12:49:55	2022-09-14 12:54:09	0	178.203.144.80	100	254	1	2022-09-14 12:54:11	R_xxUhhTe7GSoaWBP	NaN	...	38.0	70.0	23.0	28.0	34.0	25.
1	2022-09-14 13:18:46	2022-09-14 13:28:02	0	84.134.91.141	100	556	1	2022-09-14 13:28:04	R_1H2dqrEoZ0MfVsd	NaN	...	69.0	71.0	66.0	28.0	24.0	0.
2	2022-09-14 14:15:12	2022-09-14 14:17:17	0	178.203.144.34	100	125	1	2022-09-14 14:17:19	R_2AZ1vnijj5jrboP	NaN	...	77.0	61.0	19.0	0.0	87.0	0.
3	2022-09-14 13:18:36	2022-09-14 13:18:44	0	84.134.91.141	4	7	0	2022-09-14 14:18:58	R_u3t5zmCiHkRD5NT	NaN	...	NaN	NaN	NaN	NaN	NaN	Na
4	2022-09-14 14:36:25	2022-09-14 14:39:29	0	137.248.77.216	100	184	1	2022-09-14 14:39:31	R_ZFbw7HNHHXZ0deV	NaN	...	76.0	78.0	16.0	58.0	34.0	0.

5 rows × 44 columns

In [45]:
```
df_day = df_day[df_day['Finished']==1]
```

In [46]:
```
df_day['VID'].unique()
```

Out[46]:
```
array([  1, 998,   3,   2,  10,   9,   4,   5,  15,   7,   8,  16,  12,
        11,  13,  19,  18,  17,  23,  24,  20,  22,  21,  29,  30,  28,
        25,  31,  26,  33,  38,  36,  39,  37,  35,  14,  34,  40,  27,
        42,  43,  45,  32,  44,  46,  47,  50,  53,  51,  58,  48,  60,
        63,  49, 480,  59,  61,  55,  62,  64,  57,  54, 102, 101, 107,
       104, 111, 105, 110, 103, 117, 113, 134, 123, 112, 133, 131, 122,
       126, 116, 119, 130, 129, 114, 108, 128, 124, 138, 120, 132, 115,
       141, 135, 137, 139, 125, 140, 143, 146, 148, 145, 127, 150, 147,
       144, 155, 151, 157, 142, 154, 162, 163, 152, 171, 164, 161, 159,
       158, 160, 166, 168, 169, 149, 165, 153, 170, 172], dtype=int64)
```

Change VID 480 to 48.

In [47]:
```
df_day.at[df_day[df_day['VID']==480].index[0],'VID']=48
```

Drop VID 998 (a test participant), change the type of "StartDate" and "EndDate" to datetime, sort the values based on "VID" and "StartDate" and reindex the dataframe.

In [48]:
```
df_day = df_day.drop(df_day[df_day['VID']==998].index)
df_day['StartDate'] = pd.to_datetime(df_day['StartDate'])
df_day['EndDate'] = pd.to_datetime(df_day['EndDate'])
df_day = df_day.sort_values(by=['VID','StartDate'])
df_day = df_day.reset_index(drop=True)
```

Find all day sessions baded on the Session-ID (using modulo 5 with rest ≠ 1) and insert the data.

In [49]:
```
day_index = []
for index,row in df_ethica_all.iterrows():
    if row["Session-ID"]%5!=1 and row["Session-ID"] < 106 and row["Session-ID"] > 0 :
        temp_vid = row['VID']
        start_range = row['Scheduled Time']
        end_range = start_range + pd.Timedelta(hours=2)
        for index2,row2 in df_day[df_day['VID']==temp_vid].iterrows():
            if row2['StartDate'] > start_range and row2['EndDate']<end_range:
                df_ethica_all.at[index, "weekday"] = row2["StartDate"].day_name()
                df_ethica_all.at[index, "StartDate"] = row2["StartDate"]
                df_ethica_all.at[index, "EndDate"] = row2["EndDate"]
                df_ethica_all.at[index, "Progress"] = row2["Progress"]
                df_ethica_all.at[index, "Duration (in seconds)"] = row2["Duration (in seconds)"]
                df_ethica_all.at[index, "Finished"] = row2["Finished"]
                df_ethica_all.at[index, "LocationLatitude"] = row2["LocationLatitude"]
                df_ethica_all.at[index, "LocationLongitude"] = row2["LocationLongitude"]

                df_ethica_all.at[index, "PBAT-1"] = row2["PBAT-1_1"]
                df_ethica_all.at[index, "PBAT-2"] = row2["PBAT-2_1"]
                df_ethica_all.at[index, "PBAT-3"] = row2["PBAT-3_1"]
                df_ethica_all.at[index, "PBAT-4"] = row2["PBAT-4_1"]
                df_ethica_all.at[index, "PBAT-5"] = row2["PBAT-5_1"]
                df_ethica_all.at[index, "PBAT-6"] = row2["PBAT-6_1"]
                df_ethica_all.at[index, "PBAT-7"] = row2["PBAT-7 _1"]
                df_ethica_all.at[index, "PBAT-8"] = row2["PBAT-8 _1"]
                df_ethica_all.at[index, "PBAT-9"] = row2["PBAT-9_1"]
                df_ethica_all.at[index, "PBAT-10"] = row2["PBAT-10_1"]
                df_ethica_all.at[index, "PBAT-11"] = row2["PBAT-11_1"]
                df_ethica_all.at[index, "PBAT-12"] = row2["PBAT-12_1"]
                df_ethica_all.at[index, "PBAT-13"] = row2["PBAT-13_1"]
                df_ethica_all.at[index, "PBAT-14"] = row2["PBAT-14_1"]
                df_ethica_all.at[index, "PBAT-15"] = row2["PBAT-15_1"]
                df_ethica_all.at[index, "PBAT-16"] = row2["PBAT-16_1"]
                df_ethica_all.at[index, "PBAT-17"] = row2["PBAT-17_1"]
                df_ethica_all.at[index, "PBAT-18"] = row2["PBAT-18_1"]

                df_ethica_all.at[index, "Core-Affect-Valence"] = row2["Core-Affect-Valence_1"]
                df_ethica_all.at[index, "Core-Affect-Arousal"] = row2["Core-Affect-Arousal_1"]

                df_ethica_all.at[index, "LifeSatisfaction"] = row2["LifeSatisfaction_1"]

                df_ethica_all.at[index, "STOPD-1"] = row2["STOPD-1_1"]
                df_ethica_all.at[index, "STOPD-2"] = row2["STOPD-2_1"]
                df_ethica_all.at[index, "STOPD-3"] = row2["STOPD-3_1"]
                df_ethica_all.at[index, "STOPD-4"] = row2["STOPD-4_1"]
                df_ethica_all.at[index, "STOPD-5"] = row2["STOPD-5_1"]

                day_index.append(index2)
                break
```

Create a file where all not inserted Entries were listed which then will be checked by one author.

In [50]:
```
df_day_drop = pd.read_csv('EMA-Monitoring.csv',skiprows=lambda x: x in [1, 2])
df_day_drop = df_day_drop[df_day_drop['Finished']==1]
df_day_drop.at[df_day_drop[df_day_drop['VID']==480].index[0],'VID']=48
df_day_drop = df_day_drop.drop(df_day_drop[df_day_drop['VID']==998].index)

df_day_drop['StartDate'] = pd.to_datetime(df_day_drop['StartDate'])
df_day_drop['EndDate'] = pd.to_datetime(df_day_drop['EndDate'])
df_day_drop = df_day_drop.sort_values(by=['VID','StartDate'])
df_day_drop = df_day_drop.reset_index(drop=True)
```

In [51]:
```
df_day_drop = df_day_drop.drop(day_index)
```

In [52]:
```
df_day_drop.to_csv('EMA-day-not-inculded.csv')
```

Check the saved files and insert the not included values which can be used. Therefore one author decided which values shoud be included or not. Therefore different categories were defined. Categories:

- exclude (value: 0)
- insert (a given timepoint is given but is empty and should be filled; value: 1)
- change (there is are two values of the prompt but it has to be changed based on the duration for answering; ; value: 3)
- add (add the value as a last entry for the day - a complete new entry; value: 4)
- add (add the entry because others were positioned at a different timeslot; value: 5)

The index of ema morning/ema day and df_ethica_all were used so that these values can be merged.

First look at the not included morning values.

In [53]:
```
df_morning_drop_author = pd.read_csv('EMA-morning-not-insert_author.csv',sep=';')
```

First have a look at the first 5 rows of the dataset and then select values which are bigger 0.

In [54]:
```
df_morning_drop_author.head()
```

Out[54]:

	index neu	Verwendung	index alt	StartDate	EndDate	Status	IPAddress	Progress	Duration (in seconds)	Finished	...	PBAT-17_2	PBAT-18_2	Core-Affect-Valence_1	Core-Affect-Arousal_1	LifeSatisfaction_1	STOPD-1_1	STOPD-2_2	ST
0	NaN	0	2	15.09.2022 11:06	15.09.2022 11:07	0	178.203.146.227	100	82	1	...	25	63	76	30	71	20	27	
1	NaN	0	197	13.10.2022 11:46	13.10.2022 11:48	0	80.187.102.68	100	102	1	...	1	38	54	90	94	0	0	
2	NaN	0	467	25.10.2022 08:26	25.10.2022 08:32	0	46.114.2.188	100	349	1	...	14	69	84	60	86	0	23	
3	NaN	0	615	21.10.2022 07:59	21.10.2022 08:04	0	84.138.242.59	100	303	1	...	83	93	79	72	95	10	0	
4	NaN	0	616	21.10.2022 08:14	21.10.2022 08:16	0	84.138.242.59	100	142	1	...	9	100	92	68	88	0	0	

5 rows × 49 columns

In [55]:
```
df_morning_drop_author = df_morning_drop_author[df_morning_drop_author['Verwendung'] > 0]
```

In [56]:
```
df_morning_drop_author
```

Out[56]:

index neu	Verwendung	index alt	StartDate	EndDate	Status	IPAddress	Progress	Duration (in seconds)	Finished	...	PBAT-17_2	PBAT-18_2	Core-Affect-Valence_1	Core-Affect-Arousal_1	LifeSatisfaction_1	STOPD-1_1	STOPD-2_2	STOPD-3_2	ST

0 rows × 49 columns

So no values have to be included for the morning sessions.

Look at the not included day values.

In [57]:
```
df_day_drop_author = pd.read_csv('EMA-day-not-inculded_author.csv',sep=';')
```

First have a look at the first 5 rows of the dataset and then select values which are bigger 0.

In [58]:
```
df_day_drop_author.head()
```

Out[58]:

	index neu	ToDo	index alt	StartDate	EndDate	Status	IPAddress	Progress	Duration (in seconds)	Finished	...	PBAT-17_1	PBAT-18_1	STOPD-1_1	STOPD-2_1	STOPD-3_1	STOPD-4_1	STOPD-5_1	Core-Affect-Arousal_1	Core-Affect-Valence_1
0	33.0	1	26	20.09.2022 19:40	20.09.2022 19:53	0	137.248.65.237	100	780	1	...	26	64	31	25	9	30	24	70	75
1	NaN	0	72	02.10.2022 16:29	02.10.2022 16:30	0	46.114.5.55	100	51	1	...	73	64	33	33	34	31	33	61	61
2	NaN	0	81	04.10.2022 16:18	04.10.2022 16:19	0	178.203.147.77	100	101	1	...	33	65	26	30	31	36	28	69	74
3	NaN	0	266	27.09.2022 15:58	27.09.2022 16:00	0	88.64.89.171	100	87	1	...	9	91	67	33	13	0	14	10	42
4	NaN	0	280	01.10.2022 15:44	01.10.2022 15:45	0	88.64.89.171	100	88	1	...	0	99	16	8	0	0	34	0	59

5 rows × 47 columns

In [59]:
```
df_day_drop_author = df_day_drop_author[df_day_drop_author['ToDo'] > 0]
```

Check for type 3

In [60]:
```
df_day_drop_author[df_day_drop_author['ToDo']==3]
```

Out[60]:

index neu	ToDo	index alt	StartDate	EndDate	Status	IPAddress	Progress	Duration (in seconds)	Finished	...	PBAT-17_1	PBAT-18_1	STOPD-1_1	STOPD-2_1	STOPD-3_1	STOPD-4_1	STOPD-5_1	Core-Affect-Arousal_1	Core-Affect-Valence_1	LifeSatisf

0 rows × 47 columns

Check for type 5 (change the position of available entries)

In [61]:
```
df_day_drop_author[df_day_drop_author['ToDo']==5]
```

Out[61]:

	index neu	ToDo	index alt	StartDate	EndDate	Status	IPAddress	Progress	Duration (in seconds)	Finished	...	PBAT-17_1	PBAT-18_1	STOPD-1_1	STOPD-2_1	STOPD-3_1	STOPD-4_1	STOPD-5_1	Core-Affect-Arousal_1
92	8193.0	5	5925	23.03.2023 19:41	23.03.2023 19:43	0	91.9.159.104	100	176	1	...	93	13	69	90	82	0	12	18
94	8259.0	5	5974	05.04.2023 22:41	05.04.2023 22:45	0	2.247.245.8	100	234	1	...	76	30	0	41	63	0	0	63
145	10917.0	5	8332	14.06.2023 18:17	14.06.2023 18:18	0	93.205.2.17	100	78	1	...	23	60	0	1	11	0	0	59
147	12816.0	5	8396	06.07.2023 15:55	06.07.2023 15:57	0	84.138.234.157	100	106	1	...	23	72	10	0	58	3	0	79
197	11970.0	5	8724	21.06.2023 13:12	21.06.2023 13:15	0	2.202.165.148	100	198	1	...	18	63	0	0	0	0	100	92
200	11980.0	5	8732	23.06.2023 12:39	23.06.2023 12:40	0	84.134.93.231	100	91	1	...	81	66	0	0	40	0	96	74
204	11991.0	5	8740	25.06.2023 16:06	25.06.2023 16:08	0	94.218.230.179	100	105	1	...	21	66	0	0	16	0	9	60
213	12015.0	5	8756	30.06.2023 12:47	30.06.2023 12:49	0	84.134.93.231	100	106	1	...	13	62	0	0	25	0	0	78
219	12027.0	5	8765	02.07.2023 19:26	02.07.2023 19:27	0	94.218.230.179	100	66	1	...	0	68	0	0	11	0	0	72
232	12067.0	5	8789	10.07.2023 19:44	10.07.2023 19:47	0	94.218.230.179	100	197	1	...	47	54	0	0	22	0	0	53
249	12335.0	5	8833	02.07.2023 11:00	02.07.2023 11:01	0	137.248.251.127	100	71	1	...	15	81	11	8	15	17	10	81
264	12387.0	5	8875	12.07.2023 17:35	12.07.2023 17:37	0	137.248.70.1	100	103	1	...	20	67	12	13	14	8	14	68
281	12210.0	5	9304	28.06.2023 11:11	28.06.2023 11:12	0	109.43.115.45	100	101	1	...	61	69	76	67	77	21	73	71
288	12232.0	5	9321	02.07.2023 17:21	02.07.2023 17:26	0	109.43.112.245	100	275	1	...	73	72	27	99	90	61	95	25

14 rows × 47 columns

First move the existing variables to their corresponding position, so that the values which have to be insert can be placed at the corresponding position.

In [62]:
```
change_variables_day = [[8192,8191],
                        [8193,8192],
                        [8259,8258],
                        [10917,10918],
                        [12816,12815],
                        [11971,11972],
                        [11970,11971],
                        [11981,11982],
                        [11980,11981],
                        [11992,11993],
                        [11991,11992],
                        [12015,12016],
                        [12027,12028],
                        [12067,12068],
                        [12335,12336],
                        [12387,12388],
                        [12210,12111],
                        [12232,12233]]
```

In [63]:
```
for i in change_variables_day:

    df_ethica_all.at[i[1], "StartDate"] = df_ethica_all.at[i[0],"StartDate"]
    df_ethica_all.at[i[1], "EndDate"] = df_ethica_all.at[i[0],"EndDate"]
    df_ethica_all.at[i[1], "Progress"] = df_ethica_all.at[i[0],"Progress"]
    df_ethica_all.at[i[1], "Duration (in seconds)"] = df_ethica_all.at[i[0],"Duration (in seconds)"]
    df_ethica_all.at[i[1], "Finished"] = df_ethica_all.at[i[0],"Finished"]
    df_ethica_all.at[i[1], "LocationLatitude"] = df_ethica_all.at[i[0],"LocationLatitude"]
    df_ethica_all.at[i[1], "LocationLongitude"] = df_ethica_all.at[i[0],"LocationLongitude"]


    df_ethica_all.at[i[1], "PBAT-1"] = df_ethica_all.at[i[0],"PBAT-1"]
    df_ethica_all.at[i[1], "PBAT-2"] = df_ethica_all.at[i[0],"PBAT-2"]
    df_ethica_all.at[i[1], "PBAT-3"] = df_ethica_all.at[i[0],"PBAT-3"]
    df_ethica_all.at[i[1], "PBAT-4"] = df_ethica_all.at[i[0],"PBAT-4"]
    df_ethica_all.at[i[1], "PBAT-5"] = df_ethica_all.at[i[0],"PBAT-5"]
    df_ethica_all.at[i[1], "PBAT-6"] = df_ethica_all.at[i[0],"PBAT-6"]
    df_ethica_all.at[i[1], "PBAT-7"] = df_ethica_all.at[i[0],"PBAT-7"]
    df_ethica_all.at[i[1], "PBAT-8"] = df_ethica_all.at[i[0],"PBAT-8"]
    df_ethica_all.at[i[1], "PBAT-9"] = df_ethica_all.at[i[0],"PBAT-9"]
    df_ethica_all.at[i[1], "PBAT-10"] = df_ethica_all.at[i[0],"PBAT-10"]
    df_ethica_all.at[i[1], "PBAT-11"] = df_ethica_all.at[i[0],"PBAT-11"]
    df_ethica_all.at[i[1], "PBAT-12"] = df_ethica_all.at[i[0],"PBAT-12"]
    df_ethica_all.at[i[1], "PBAT-13"] = df_ethica_all.at[i[0],"PBAT-13"]
    df_ethica_all.at[i[1], "PBAT-14"] = df_ethica_all.at[i[0],"PBAT-14"]
    df_ethica_all.at[i[1], "PBAT-15"] = df_ethica_all.at[i[0],"PBAT-15"]
    df_ethica_all.at[i[1], "PBAT-16"] = df_ethica_all.at[i[0],"PBAT-16"]
    df_ethica_all.at[i[1], "PBAT-17"] = df_ethica_all.at[i[0],"PBAT-17"]
    df_ethica_all.at[i[1], "PBAT-18"] = df_ethica_all.at[i[0],"PBAT-18"]

    df_ethica_all.at[i[1], "Core-Affect-Valence"] = df_ethica_all.at[i[0],"Core-Affect-Valence"]
    df_ethica_all.at[i[1], "Core-Affect-Arousal"] = df_ethica_all.at[i[0],"Core-Affect-Arousal"]

    df_ethica_all.at[i[1], "LifeSatisfaction"] = df_ethica_all.at[i[0],"LifeSatisfaction"]

    df_ethica_all.at[i[1], "STOPD-1"] = df_ethica_all.at[i[0],"STOPD-1"]
    df_ethica_all.at[i[1], "STOPD-2"] = df_ethica_all.at[i[0],"STOPD-2"]
    df_ethica_all.at[i[1], "STOPD-3"] = df_ethica_all.at[i[0],"STOPD-3"]
    df_ethica_all.at[i[1], "STOPD-4"] = df_ethica_all.at[i[0],"STOPD-4"]
    df_ethica_all.at[i[1], "STOPD-5"] = df_ethica_all.at[i[0],"STOPD-5"]
```

Now get the index of the old position and the new position of the entry to place it at the right position.

In [64]:
```
index_new = df_day_drop_author[df_day_drop_author['ToDo']==5]['index neu'].tolist()
index_old = df_day_drop_author[df_day_drop_author['ToDo']==5]['index alt'].tolist()

index_list_day = []
for i  in range(len(index_new)):
```

```
    index_list_day.append([index_old[i],index_new[i]])
```

In [65]:
```
index_list_day
```

Out[65]:
```
[[5925, 8193.0],
 [5974, 8259.0],
 [8332, 10917.0],
 [8396, 12816.0],
 [8724, 11970.0],
 [8732, 11980.0],
 [8740, 11991.0],
 [8756, 12015.0],
 [8765, 12027.0],
 [8789, 12067.0],
 [8833, 12335.0],
 [8875, 12387.0],
 [9304, 12210.0],
 [9321, 12232.0]]
```

In [66]:
```
for i in index_list_day:

    df_ethica_all.at[i[1], "StartDate"] = df_day.at[i[0],"StartDate"]
    df_ethica_all.at[i[1], "EndDate"] = df_day.at[i[0],"EndDate"]
    df_ethica_all.at[i[1], "Progress"] = df_day.at[i[0],"Progress"]
    df_ethica_all.at[i[1], "Duration (in seconds)"] = df_day.at[i[0],"Duration (in seconds)"]
    df_ethica_all.at[i[1], "Finished"] = df_day.at[i[0],"Finished"]
    df_ethica_all.at[i[1], "LocationLatitude"] = df_day.at[i[0],"LocationLatitude"]
    df_ethica_all.at[i[1], "LocationLongitude"] = df_day.at[i[0],"LocationLongitude"]


    df_ethica_all.at[i[1], "PBAT-1"] = df_day.at[i[0],"PBAT-1_1"]
    df_ethica_all.at[i[1], "PBAT-2"] = df_day.at[i[0],"PBAT-2_1"]
    df_ethica_all.at[i[1], "PBAT-3"] = df_day.at[i[0],"PBAT-3_1"]
    df_ethica_all.at[i[1], "PBAT-4"] = df_day.at[i[0],"PBAT-4_1"]
    df_ethica_all.at[i[1], "PBAT-5"] = df_day.at[i[0],"PBAT-5_1"]
    df_ethica_all.at[i[1], "PBAT-6"] = df_day.at[i[0],"PBAT-6_1"]
    df_ethica_all.at[i[1], "PBAT-7"] = df_day.at[i[0],"PBAT-7 _1"]
    df_ethica_all.at[i[1], "PBAT-8"] = df_day.at[i[0],"PBAT-8 _1"]
    df_ethica_all.at[i[1], "PBAT-9"] = df_day.at[i[0],"PBAT-9_1"]
    df_ethica_all.at[i[1], "PBAT-10"] = df_day.at[i[0],"PBAT-10_1"]
    df_ethica_all.at[i[1], "PBAT-11"] = df_day.at[i[0],"PBAT-11_1"]
    df_ethica_all.at[i[1], "PBAT-12"] = df_day.at[i[0],"PBAT-12_1"]
    df_ethica_all.at[i[1], "PBAT-13"] = df_day.at[i[0],"PBAT-13_1"]
    df_ethica_all.at[i[1], "PBAT-14"] = df_day.at[i[0],"PBAT-14_1"]
    df_ethica_all.at[i[1], "PBAT-15"] = df_day.at[i[0],"PBAT-15_1"]
    df_ethica_all.at[i[1], "PBAT-16"] = df_day.at[i[0],"PBAT-16_1"]
    df_ethica_all.at[i[1], "PBAT-17"] = df_day.at[i[0],"PBAT-17_1"]
    df_ethica_all.at[i[1], "PBAT-18"] = df_day.at[i[0],"PBAT-18_1"]

    df_ethica_all.at[i[1], "Core-Affect-Valence"] = df_day.at[i[0],"Core-Affect-Valence_1"]
    df_ethica_all.at[i[1], "Core-Affect-Arousal"] = df_day.at[i[0],"Core-Affect-Arousal_1"]

    df_ethica_all.at[i[1], "LifeSatisfaction"] = df_day.at[i[0],"LifeSatisfaction_1"]

    df_ethica_all.at[i[1], "STOPD-1"] = df_day.at[i[0],"STOPD-1_1"]
    df_ethica_all.at[i[1], "STOPD-2"] = df_day.at[i[0],"STOPD-2_1"]
    df_ethica_all.at[i[1], "STOPD-3"] = df_day.at[i[0],"STOPD-3_1"]
    df_ethica_all.at[i[1], "STOPD-4"] = df_day.at[i[0],"STOPD-4_1"]
    df_ethica_all.at[i[1], "STOPD-5"] = df_day.at[i[0],"STOPD-5_1"]
```

Check for type 1.

In [67]:
```
df_day_drop_author[df_day_drop_author['ToDo']==1]
```

Out[67]:

	index neu	ToDo	index alt	StartDate	EndDate	Status	IPAddress	Progress	Duration (in seconds)	Finished	...	PBAT-17_1	PBAT-18_1	STOPD-1_1	STOPD-2_1	STOPD-3_1	STOPD-4_1	STOPD-5_1	Core-Affect-Arousal_1
**0**	33.0	1	26	20.09.2022 19:40	20.09.2022 19:53	0	137.248.65.237	100	780	1	...	26	64	31	25	9	30	24	70
**5**	808.0	1	456	07.10.2022 20:04	07.10.2022 20:07	0	82.113.106.103	100	184	1	...	0	82	0	0	0	0	0	25
**7**	833.0	1	475	12.10.2022 20:52	12.10.2022 20:56	0	46.114.178.177	100	255	1	...	21	69	0	0	0	0	12	89
**8**	848.0	1	487	24.09.2022 19:06	24.09.2022 19:08	0	84.134.94.9	100	123	1	...	34	73	0	0	0	0	45	24
**9**	851.0	1	489	25.09.2022 12:59	25.09.2022 13:02	0	84.134.94.9	100	173	1	...	64	74	0	48	38	0	11	69
**...**	...	...	...	...	...	...	...	...	...	...	...	...	...	...	...	...	...	...	...
**305**	12281.0	1	9359	12.07.2023 13:42	12.07.2023 13:44	0	95.90.221.226	100	75	1	...	92	17	92	13	75	11	92	59
**307**	12522.0	1	9468	03.07.2023 19:23	03.07.2023 20:13	0	137.248.4.247	100	2980	1	...	28	65	16	0	4	0	3	69
**308**	12548.0	1	9488	08.07.2023 23:26	08.07.2023 23:29	0	109.43.50.95	100	155	1	...	53	23	59	0	46	88	7	60
**309**	12562.0	1	9499	11.07.2023 19:06	11.07.2023 19:40	0	95.223.184.106	100	2039	1	...	28	31	0	0	20	0	0	57
**310**	12938.0	1	9544	10.07.2023 00:50	10.07.2023 00:53	0	79.214.137.74	100	172	1	...	18	57	0	0	0	0	66	0

240 rows × 47 columns

Create a list with the old and new index position.

In [68]:
```
index_new = df_day_drop_author[df_day_drop_author['ToDo']==1]['index neu'].tolist()
index_old = df_day_drop_author[df_day_drop_author['ToDo']==1]['index alt'].tolist()

index_list_day = []
for i  in range(len(index_new)):
    index_list_day.append([index_old[i],index_new[i]])
```

In [69]:
```
for i in index_list_day:

    df_ethica_all.at[i[1], "StartDate"] = df_day.at[i[0],"StartDate"]
    df_ethica_all.at[i[1], "EndDate"] = df_day.at[i[0],"EndDate"]
    df_ethica_all.at[i[1], "Progress"] = df_day.at[i[0],"Progress"]
    df_ethica_all.at[i[1], "Duration (in seconds)"] = df_day.at[i[0],"Duration (in seconds)"]
    df_ethica_all.at[i[1], "Finished"] = df_day.at[i[0],"Finished"]
    df_ethica_all.at[i[1], "LocationLatitude"] = df_day.at[i[0],"LocationLatitude"]
    df_ethica_all.at[i[1], "LocationLongitude"] = df_day.at[i[0],"LocationLongitude"]


    df_ethica_all.at[i[1], "PBAT-1"] = df_day.at[i[0],"PBAT-1_1"]
    df_ethica_all.at[i[1], "PBAT-2"] = df_day.at[i[0],"PBAT-2_1"]
    df_ethica_all.at[i[1], "PBAT-3"] = df_day.at[i[0],"PBAT-3_1"]
    df_ethica_all.at[i[1], "PBAT-4"] = df_day.at[i[0],"PBAT-4_1"]
    df_ethica_all.at[i[1], "PBAT-5"] = df_day.at[i[0],"PBAT-5_1"]
    df_ethica_all.at[i[1], "PBAT-6"] = df_day.at[i[0],"PBAT-6_1"]
    df_ethica_all.at[i[1], "PBAT-7"] = df_day.at[i[0],"PBAT-7 _1"]
    df_ethica_all.at[i[1], "PBAT-8"] = df_day.at[i[0],"PBAT-8 _1"]
    df_ethica_all.at[i[1], "PBAT-9"] = df_day.at[i[0],"PBAT-9_1"]
    df_ethica_all.at[i[1], "PBAT-10"] = df_day.at[i[0],"PBAT-10_1"]
    df_ethica_all.at[i[1], "PBAT-11"] = df_day.at[i[0],"PBAT-11_1"]
    df_ethica_all.at[i[1], "PBAT-12"] = df_day.at[i[0],"PBAT-12_1"]
    df_ethica_all.at[i[1], "PBAT-13"] = df_day.at[i[0],"PBAT-13_1"]
    df_ethica_all.at[i[1], "PBAT-14"] = df_day.at[i[0],"PBAT-14_1"]
    df_ethica_all.at[i[1], "PBAT-15"] = df_day.at[i[0],"PBAT-15_1"]
    df_ethica_all.at[i[1], "PBAT-16"] = df_day.at[i[0],"PBAT-16_1"]
    df_ethica_all.at[i[1], "PBAT-17"] = df_day.at[i[0],"PBAT-17_1"]
    df_ethica_all.at[i[1], "PBAT-18"] = df_day.at[i[0],"PBAT-18_1"]

    df_ethica_all.at[i[1], "Core-Affect-Valence"] = df_day.at[i[0],"Core-Affect-Valence_1"]
    df_ethica_all.at[i[1], "Core-Affect-Arousal"] = df_day.at[i[0],"Core-Affect-Arousal_1"]

    df_ethica_all.at[i[1], "LifeSatisfaction"] = df_day.at[i[0],"LifeSatisfaction_1"]

    df_ethica_all.at[i[1], "STOPD-1"] = df_day.at[i[0],"STOPD-1_1"]
    df_ethica_all.at[i[1], "STOPD-2"] = df_day.at[i[0],"STOPD-2_1"]
    df_ethica_all.at[i[1], "STOPD-3"] = df_day.at[i[0],"STOPD-3_1"]
    df_ethica_all.at[i[1], "STOPD-4"] = df_day.at[i[0],"STOPD-4_1"]
    df_ethica_all.at[i[1], "STOPD-5"] = df_day.at[i[0],"STOPD-5_1"]
```

Check for type 4 (a complete new entry)

In [70]:
```
df_day_drop_author[df_day_drop_author['ToDo']==4]
```

Out[70]:

	index neu	ToDo	index alt	StartDate	EndDate	Status	IPAddress	Progress	Duration (in seconds)	Finished	...	PBAT-17_1	PBAT-18_1	STOPD-1_1	STOPD-2_1	STOPD-3_1	STOPD-4_1	STOPD-5_1	Core-Affect-Arousal_1
**102**	10499.0	4	6528	07.06.2023 21:27	07.06.2023 21:32	0	95.223.185.179	100	290	1	...	65	62	65	0	0	0	0	23

1 rows × 47 columns

This entry must get the Session ID 105

In [71]:
```
df_ethica_all.tail()
```

Out[71]:

	Name	Scheduled Time	Response Time	VID	Session-ID	Day	Session	weekday	LocationLatitude	LocationLongitude	...	Core-Affect-Valence	Core-Affect-Arousal	LifeSatisfaction	STOPD-1	STOPD-2	STOPD-3
**13370**	NaN	NaT	NaT	165.0	106	NaN	NaN	nan	50.7996	8.7669	...	NaN	NaN	NaN	NaN	NaN	NaN
**13371**	NaN	NaT	NaT	149.0	106	NaN	NaN	nan	50.7996	8.7669	...	NaN	NaN	NaN	NaN	NaN	NaN
**13372**	NaN	NaT	NaT	153.0	106	NaN	NaN	nan	50.7996	8.7669	...	NaN	NaN	NaN	NaN	NaN	NaN
**13373**	NaN	NaT	NaT	170.0	106	NaN	NaN	nan	50.7996	8.7669	...	NaN	NaN	NaN	NaN	NaN	NaN
**13374**	NaN	NaT	NaT	172.0	106	NaN	NaN	nan	50.7996	8.7669	...	NaN	NaN	NaN	NaN	NaN	NaN

5 rows × 285 columns

In [72]:
```
 new_row = {'VID':df_day.at[6528,'VID'],
          'StartDate':df_day.at[6528,'StartDate'],
          'EndDate':df_day.at[6528,'EndDate'],
          'Progress':df_day.at[6528,'Progress'],
          'Duration (in seconds)':df_day.at[6528,'Duration (in seconds)'],
          'Finished':df_day.at[6528,'Finished'],
          'LocationLatitude':df_day.at[6528,'LocationLatitude'],
          'LocationLongitude':df_day.at[6528,'LocationLongitude'],
          'Session-ID' : 105,

          'PBAT-1':df_day.at[6528,"PBAT-1_1"],
          'PBAT-2':df_day.at[6528,"PBAT-2_1"],
          'PBAT-3':df_day.at[6528,"PBAT-3_1"],
          'PBAT-4':df_day.at[6528,"PBAT-4_1"],
          'PBAT-5':df_day.at[6528,"PBAT-5_1"],
          'PBAT-6':df_day.at[6528,"PBAT-6_1"],
          'PBAT-7':df_day.at[6528,"PBAT-7 _1"],
          'PBAT-8':df_day.at[6528,"PBAT-8 _1"],
          'PBAT-9':df_day.at[6528,"PBAT-9_1"],
          'PBAT-10':df_day.at[6528,"PBAT-10_1"],
          'PBAT-11':df_day.at[6528,"PBAT-11_1"],
          'PBAT-12':df_day.at[6528,"PBAT-12_1"],
          'PBAT-13':df_day.at[6528,"PBAT-13_1"],
          'PBAT-14':df_day.at[6528,"PBAT-14_1"],
          'PBAT-15':df_day.at[6528,"PBAT-15_1"],
          'PBAT-16':df_day.at[6528,"PBAT-16_1"],
          'PBAT-17':df_day.at[6528,"PBAT-17_1"],
          'PBAT-18':df_day.at[6528,"PBAT-18_1"],

          "Core-Affect-Valence":df_day.at[6528,"Core-Affect-Valence_1"],
          "Core-Affect-Arousal":df_day.at[6528,"Core-Affect-Arousal_1"],

          "LifeSatisfaction":df_day.at[6528,"LifeSatisfaction_1"],

          "STOPD-1": df_day.at[6528,"STOPD-1_1"],
          "STOPD-2": df_day.at[6528,"STOPD-2_1"],
          "STOPD-3": df_day.at[6528,"STOPD-3_1"],
          "STOPD-4": df_day.at[6528,"STOPD-4_1"],
          "STOPD-5": df_day.at[6528,"STOPD-5_1"]
          }
df_ethica_all = pd.concat([df_ethica_all, pd.DataFrame([new_row])], ignore_index=True)
```

In [73]:
```
df_ethica_all.tail()
```

Out[73]:

	Name	Scheduled Time	Response Time	VID	Session-ID	Day	Session	weekday	LocationLatitude	LocationLongitude	...	Core-Affect-Valence	Core-Affect-Arousal	LifeSatisfaction	STOPD-1	STOPD-2	STOPD-3
**13371**	NaN	NaT	NaT	149.0	106	NaN	NaN	nan	50.7996	8.7669	...	NaN	NaN	NaN	NaN	NaN	NaN
**13372**	NaN	NaT	NaT	153.0	106	NaN	NaN	nan	50.7996	8.7669	...	NaN	NaN	NaN	NaN	NaN	NaN
**13373**	NaN	NaT	NaT	170.0	106	NaN	NaN	nan	50.7996	8.7669	...	NaN	NaN	NaN	NaN	NaN	NaN
**13374**	NaN	NaT	NaT	172.0	106	NaN	NaN	nan	50.7996	8.7669	...	NaN	NaN	NaN	NaN	NaN	NaN
**13375**	NaN	NaT	NaT	127.0	105	NaN	NaN	NaN	50.5866	8.6771	...	26.0	23.0	48.0	65.0	0.0	0.0

5 rows × 285 columns

Add number of the day for all the entries in the main dataframe.

In [74]:
```
for index,row in df_ethica_all.iterrows():
    if(row['Session-ID']>=1 and row['Session-ID']<=5):
        df_ethica_all.at[index,"Day"] = 1
    if(row['Session-ID']>=6 and row['Session-ID']<=10):
        df_ethica_all.at[index,"Day"] = 2
    if(row['Session-ID']>=11 and row['Session-ID']<=15):
        df_ethica_all.at[index,"Day"] = 3
    if(row['Session-ID']>=16 and row['Session-ID']<=20):
        df_ethica_all.at[index,"Day"] = 4
    if(row['Session-ID']>=21 and row['Session-ID']<=25):
        df_ethica_all.at[index,"Day"] = 5
    if(row['Session-ID']>=26 and row['Session-ID']<=30):
        df_ethica_all.at[index,"Day"] = 6
    if(row['Session-ID']>=31 and row['Session-ID']<=35):
        df_ethica_all.at[index,"Day"] = 7
    if(row['Session-ID']>=36 and row['Session-ID']<=40):
        df_ethica_all.at[index,"Day"] = 8
    if(row['Session-ID']>=41 and row['Session-ID']<=45):
        df_ethica_all.at[index,"Day"] = 9
    if(row['Session-ID']>=46 and row['Session-ID']<=50):
        df_ethica_all.at[index,"Day"] = 10
    if(row['Session-ID']>=51 and row['Session-ID']<=55):
        df_ethica_all.at[index,"Day"] = 11
    if(row['Session-ID']>=56 and row['Session-ID']<=60):
        df_ethica_all.at[index,"Day"] = 12
    if(row['Session-ID']>=61 and row['Session-ID']<=65):
        df_ethica_all.at[index,"Day"] = 13
    if(row['Session-ID']>=66 and row['Session-ID']<=70):
        df_ethica_all.at[index,"Day"] = 14
    if(row['Session-ID']>=71 and row['Session-ID']<=75):
        df_ethica_all.at[index,"Day"] = 15
    if(row['Session-ID']>=76 and row['Session-ID']<=80):
        df_ethica_all.at[index,"Day"] = 16
    if(row['Session-ID']>=81 and row['Session-ID']<=85):
        df_ethica_all.at[index,"Day"] = 17
    if(row['Session-ID']>=86 and row['Session-ID']<=90):
        df_ethica_all.at[index,"Day"] = 18
    if(row['Session-ID']>=91 and row['Session-ID']<=95):
        df_ethica_all.at[index,"Day"] = 19
    if(row['Session-ID']>=96 and row['Session-ID']<=100):
        df_ethica_all.at[index,"Day"] = 20
    if(row['Session-ID']>=101 and row['Session-ID']<=105):
        df_ethica_all.at[index,"Day"] = 21
```

Add demographics for all entries in the main dataframe.

In [75]:
```
sub_list = df_ethica_all["VID"].unique().tolist()
```

In [76]:
```
for i in sub_list:
    try:
        index_sub = df_ethica_all[(df_ethica_all["VID"]==i)&(df_ethica_all["Session-ID"]==0)].index
        gender = df_ethica_all.at[index_sub[0],"Gender"]
        age =  df_ethica_all.at[index_sub[0],"Age"]
        relationship =  df_ethica_all.at[index_sub[0],"Relationship"]
        livingstatus =  df_ethica_all.at[index_sub[0],"Livingstatus"]
        degree = df_ethica_all.at[index_sub[0],"Degree"]
        occupation =  df_ethica_all.at[index_sub[0],"Occupation"]

        for index,row in df_ethica_all[(df_ethica_all["VID"]==i)].iterrows():
            df_ethica_all.at[index,"Gender"] = gender
            df_ethica_all.at[index,"Age"] = age
            df_ethica_all.at[index,"Relationship"] = relationship
            df_ethica_all.at[index,"Livingstatus"] = livingstatus
            df_ethica_all.at[index,"Degree"] = degree
            df_ethica_all.at[index,"Occupation"] = occupation
    except:
        print(i)
```

Sort the main dataframe based on "VID" and "Session-ID" and reindex the dataframe.

In [77]:
```
df_ethica_all = df_ethica_all.sort_values(by=['VID','Session-ID'])
df_ethica_all = df_ethica_all.reset_index(drop=True)
```

Based on the insertion the weekday has to be reset for VID 127 at Session-ID 105.

In [78]:
```
df_ethica_all[df_ethica_all['VID']==127]
```

Out[78]:

	Name	Scheduled Time	Response Time	VID	Session-ID	Day	Session	weekday	LocationLatitude	LocationLongitude	...	Core-Affect-Valence	Core-Affect-Arousal	LifeSatisfaction	STOPD-1	STOPD-2	STOPD-3
**8774**	NaN	NaT	NaT	127.0	0	NaN	NaN	nan	50.7996	8.7669	...	NaN	NaN	NaN	NaN	NaN	NaN
**8775**	72424.0	2023-05-18 09:02:31	2023-05-18 09:17:45	127.0	1	1.0	1.0	Thursday	50.5866	8.6771	...	69.0	29.0	73.0	12.0	0.0	26.0
**8776**	72424.0	2023-05-18 12:19:13	2023-05-18 12:27:08	127.0	2	1.0	-1.0	Thursday	51.4444	7.0114	...	76.0	25.0	80.0	15.0	0.0	0.0
**8777**	72424.0	2023-05-18 14:31:25	2023-05-18 14:37:04	127.0	3	1.0	-1.0	Thursday	51.4444	7.0114	...	82.0	36.0	84.0	0.0	0.0	0.0
**8778**	72424.0	2023-05-18 18:50:28	2023-05-18 19:01:04	127.0	4	1.0	-1.0	Thursday	48.7670	9.1827	...	65.0	51.0	100.0	0.0	0.0	0.0
**...**	...	...	...	...	...	...	...	...	...	...	...	...	...	...	...	...	...
**8876**	72424.0	2023-06-07 11:45:35	2023-06-07 12:22:00	127.0	102	21.0	-1.0	Wednesday	50.5866	8.6771	...	34.0	48.0	32.0	73.0	0.0	0.0
**8877**	72424.0	2023-06-07 15:09:02	2023-06-07 15:14:07	127.0	103	21.0	-1.0	Wednesday	53.5544	9.9960	...	57.0	52.0	29.0	68.0	0.0	0.0
**8878**	72424.0	2023-06-07 17:37:57	2023-06-07 18:20:54	127.0	104	21.0	-1.0	Wednesday	53.5544	9.9960	...	59.0	61.0	50.0	60.0	0.0	0.0
**8879**	NaN	NaT	NaT	127.0	105	21.0	NaN	NaN	50.5866	8.6771	...	26.0	23.0	48.0	65.0	0.0	0.0
**8880**	NaN	NaT	NaT	127.0	106	NaN	NaN	nan	50.7996	8.7669	...	NaN	NaN	NaN	NaN	NaN	NaN

107 rows × 285 columns

In [79]:
```
df_ethica_all.at[8879, 'weekday'] = df_ethica_all.loc[8879]["StartDate"].day_name()
```

Set number of day, Session-ID and weekday for the intro sessions in the main dataframe.

In [80]:
```
for i in df_ethica_all[df_ethica_all['Session-ID']==0].index:
    df_ethica_all.at[i, "Day"] = 0
    df_ethica_all.at[i, "Session"] = 1
    df_ethica_all.at[i, 'weekday'] = datetime.strptime(df_ethica_all.iloc[i]["StartDate"], '%Y-%m-%d %H:%M:%S').strftime("%A")
```

Set number of day, Session-ID and weekday for the exit sessions in the main dataframe.

In [81]:
```
for i in df_ethica_all[df_ethica_all['Session-ID']==106].index:
    df_ethica_all.at[i, "Day"] = 22
    df_ethica_all.at[i, "Session"] = 1
    df_ethica_all.at[i, 'weekday'] = datetime.strptime(df_ethica_all.iloc[i]["StartDate"], '%Y-%m-%d %H:%M:%S').strftime("%A")
```

Set now the Session for each day (from 1-5).

In [82]:
```
session_ids = [x for x in range(1,106)]
```

In [83]:
```
for i in session_ids:
    next_day = i%5
    counter = 1
    if next_day == 0:
        for i in df_ethica_all[df_ethica_all['Session-ID']==i].index:
            df_ethica_all.at[i, "Session"] = 5
        counter = counter +1

    else:
        for i in df_ethica_all[df_ethica_all['Session-ID']==i].index:
            df_ethica_all.at[i, "Session"] = next_day
```

In [84]:
```
df_ethica_all.head()
```

Out[84]:

	Name	Scheduled Time	Response Time	VID	Session-ID	Day	Session	weekday	LocationLatitude	LocationLongitude	...	Core-Affect-Valence	Core-Affect-Arousal	LifeSatisfaction	STOPD-1	STOPD-2	STOPD-3	STOPD-4
**0**	NaN	NaT	NaT	1.0	0	0.0	1.0	Tuesday	50.8021	8.7857	...	NaN	NaN	NaN	NaN	NaN	NaN	NaN
**1**	59981.0	2022-09-14 09:55:28	2022-09-14 10:01:15	1.0	1	1.0	1.0	Wednesday	50.5866	8.6771	...	92.0	71.0	85.0	19.0	12.0	18.0	19.0
**2**	59981.0	2022-09-14 12:46:24	2022-09-14 12:54:14	1.0	2	1.0	2.0	Wednesday	50.5866	8.6771	...	74.0	80.0	85.0	23.0	28.0	34.0	25.0
**3**	59981.0	2022-09-14 15:10:30	2022-09-14 15:59:13	1.0	3	1.0	3.0	Wednesday	50.5866	8.6771	...	72.0	34.0	69.0	23.0	28.0	26.0	23.0
**4**	59981.0	2022-09-14 18:05:56	2022-09-14 18:49:16	1.0	4	1.0	4.0	Wednesday	50.5866	8.6771	...	67.0	17.0	83.0	24.0	29.0	59.0	22.0

5 rows × 285 columns

2) Datacleaing of the data

Copy the dataframe and find double entries in the main dataframe.

In [85]:
```
df_cleaning = df_ethica_all.copy()
```

In [86]:
```
same_values = []
same_values2 = []
for index,row in df_cleaning.iterrows():
    try:
        if (df_cleaning.at[index,'StartDate'] == df_cleaning.at[index+1,'StartDate']) and (df_cleaning.at[index,'StartDate'] != -1):
            same_values.append([index,index+1])
            same_values2.append(index)
            same_values2.append(index+1)
    except:
        print (row['Session-ID'])
```

```
106
```

Save double values to file and inspect them manually by one author.

In [87]:
```
df_cleaning[df_cleaning.index.isin(same_values2)].to_csv('double_values.csv')
```

After manual inspection the file is loaded into a dataframe and the data, which is maked as "Delete" == 1 must be deleted (set -1).

In [88]:
```
df_double_values = pd.read_csv("double_values_author.csv",sep=';')
```

In [89]:
```
df_double_values.head()
```

Out[89]:

	Delete	Index	Name	Scheduled Time	Response Time	VID	StartDate	EndDate	Progress	Duration (in seconds)	...	CovidImpactsQuesti-Resource	CovidImpactsQuesti-Psychological	SFHS-Physical-Functioning	SFHS-Role-Health	SFHS-Role-Emotional	SFHS-Energy-Fatigue
**0**	NaN	369	60195.0	30.09.22 16:39	30.09.22 18:34	4.0	30.09.22 18:31	30.09.22 18:34	100	188	...	-1	-1	-1.0	-1.0	-1.0	-1.0
**1**	1.0	370	60195.0	30.09.22 18:31	30.09.22 18:31	4.0	30.09.22 18:31	30.09.22 18:34	100	188	...	-1	-1	-1.0	-1.0	-1.0	-1.0
**2**	NaN	1122	60371.0	08.10.22 14:05	08.10.22 15:57	12.0	08.10.22 15:56	08.10.22 15:57	100	110	...	-1	-1	-1.0	-1.0	-1.0	-1.0
**3**	1.0	1123	60371.0	08.10.22 15:30	08.10.22 15:30	12.0	08.10.22 15:56	08.10.22 15:57	100	110	...	-1	-1	-1.0	-1.0	-1.0	-1.0
**4**	NaN	1138	60371.0	11.10.22 16:54	11.10.22 18:50	12.0	11.10.22 18:47	11.10.22 18:50	100	209	...	-1	-1	-1.0	-1.0	-1.0	-1.0

5 rows × 311 columns

In [90]:
```
double_index_list = df_double_values[df_double_values['Delete']==1]['Index'].tolist()
```

In [91]:
```
for index in double_index_list:
    print(index)
    df_cleaning.at[index, "weekday"] = -1
    df_cleaning.at[index, "StartDate"] = -1
    df_cleaning.at[index, "EndDate"] = -1
    df_cleaning.at[index, "Progress"] = -1
    df_cleaning.at[index, "Duration (in seconds)"] = -1
    df_cleaning.at[index, "Finished"] = -1
    df_cleaning.at[index, "LocationLatitude"] = -1
    df_cleaning.at[index, "LocationLongitude"] = -1

    df_cleaning.at[index, "PBAT-1"] = -1
    df_cleaning.at[index, "PBAT-2"] = -1
    df_cleaning.at[index, "PBAT-3"] = -1
    df_cleaning.at[index, "PBAT-4"] = -1
    df_cleaning.at[index, "PBAT-5"] = -1
    df_cleaning.at[index, "PBAT-6"] = -1
    df_cleaning.at[index, "PBAT-7"] = -1
    df_cleaning.at[index, "PBAT-8"] = -1
    df_cleaning.at[index, "PBAT-9"] = -1
    df_cleaning.at[index, "PBAT-10"] = -1
    df_cleaning.at[index, "PBAT-11"] = -1
    df_cleaning.at[index, "PBAT-12"] = -1
    df_cleaning.at[index, "PBAT-13"] = -1
    df_cleaning.at[index, "PBAT-14"] = -1
    df_cleaning.at[index, "PBAT-15"] = -1
    df_cleaning.at[index, "PBAT-16"] = -1
    df_cleaning.at[index, "PBAT-17"] = -1
    df_cleaning.at[index, "PBAT-18"] = -1

    df_cleaning.at[index, "Core-Affect-Valence"] = -1
    df_cleaning.at[index, "Core-Affect-Arousal"] = -1

    df_cleaning.at[index, "LifeSatisfaction"] = -1

    df_cleaning.at[index, "STOPD-1"] = -1
    df_cleaning.at[index, "STOPD-2"] = -1
    df_cleaning.at[index, "STOPD-3"] = -1
    df_cleaning.at[index, "STOPD-4"] = -1
    df_cleaning.at[index, "STOPD-5"] = -1
```

```
370
1123
1139
1582
1643
2037
2336
2444
2534
2760
2860
3260
3518
3741
4134
5332
5363
5394
5405
5450
5511
5527
5736
5760
6129
6713
7156
7356
7443
7548
7563
8135
8180
8610
8650
10119
10303
10736
10821
10861
11000
11163
11189
11320
11391
11510
11897
12533
12610
13001
13349
```

Calculate the compliance rate

Therefore count the -1 entries for each subject. Print the VID if the compliance rate is below 70%.

In [92]:
```
subject_list = df_cleaning['VID'].unique().tolist()
```

In [93]:
```
for i in range(len(subject_list)):

    all_promts = df_cleaning[df_cleaning['VID'] == subject_list[i]]['Duration (in seconds)'].count() -1
    try:
        missing_promps = df_cleaning[df_cleaning['VID'] == subject_list[i]]['Duration (in seconds)'].value_counts()[-1]
    except:
        missing_promps = 0

    rate= ((all_promts - missing_promps) / all_promts) *100

    if rate < 70:
        print('VID: '+str(subject_list[i]))
        print (rate)
```

```
VID: 120.0
57.54716981132076
VID: 158.0
41.509433962264154
```

Check closure items

Closure item 3-1

Check if there are items which are >= 4 and print them.

In [94]:
```
for i in range(len(subject_list)):


    close_1 = df_cleaning[(df_cleaning['VID'] == subject_list[i]) & (df_cleaning['Session-ID'] == 106)]['Closure-3-1']
    if close_1.iloc[0] >= 4:
        print('VID: '+str(subject_list[i]))
        print(close_1.iloc[0])
```

```
VID: 1.0
4.0
VID: 16.0
4.0
VID: 38.0
4.0
VID: 158.0
4.0
```

Closure item 3-2

Check if there are items which are < 4 and print them.

In [95]:
```
for i in range(len(subject_list)):

    close_2 = df_cleaning[(df_cleaning['VID'] == subject_list[i]) & (df_cleaning['Session-ID'] == 106)]['Closure-3-2']
    if close_2.iloc[0] < 4:
        print('VID: '+str(subject_list[i]))
        print(close_2.iloc[0])
```

```
VID: 160.0
1.0
```

Closure item 3-3

Check if there are items which are < 4 and print them.

In [96]:
```
for i in range(len(subject_list)):

    close_3 = df_cleaning[(df_cleaning['VID'] == subject_list[i]) & (df_cleaning['Session-ID'] == 106)]['Closure-3-3']
    if close_3.iloc[0] < 4:
        print('VID: '+str(subject_list[i]))
        print(close_3.iloc[0])
```

```
VID: 158.0
2.0
VID: 160.0
2.0
```

Closure item 3-6

Check if there are items which are ≠ 4 and print them.

In [97]:
```
for i in range(len(subject_list)):

    close_6 = df_cleaning[(df_cleaning['VID'] == subject_list[i]) & (df_cleaning['Session-ID'] == 106)]['Closure-3-6']
    if close_6.iloc[0] != 4:
        print('VID: '+str(subject_list[i]))
        print(close_6.iloc[0])
```

```
VID: 9.0
nan
VID: 26.0
2.0
VID: 111.0
2.0
```

Delete the subjects based on the items

Subjects will be deleted for EMA:

- 1
- 9
- 16
- 26
- 38
- 111
- 120
- 158
- 160

Select the needed columns for the EMA dataset.

In [98]:
```
drop_cols_ema = ['AAQ-1' , 'AAQ-2' , 'AAQ-3' , 'AAQ-4' , 'AAQ-5' , 'AAQ-6' , 'AAQ-7' , 'ExpCloseRelat-1' , 'ExpCloseRelat-2' , 'ExpCloseRelat-3' , 'ExpCloseRelat-4' , '
```

Save EMA data.

In [99]:
```
df_cleaning_ema = df_cleaning.drop(drop_cols_ema, axis=1)
```

Drop the VIDs who did not pass the datacheck.

In [100…
```
df_cleaning_ema = df_cleaning_ema.drop(df_cleaning_ema[df_cleaning_ema['VID']== 1].index)
df_cleaning_ema = df_cleaning_ema.drop(df_cleaning_ema[df_cleaning_ema['VID']== 9].index)
df_cleaning_ema = df_cleaning_ema.drop(df_cleaning_ema[df_cleaning_ema['VID']== 16].index)
df_cleaning_ema = df_cleaning_ema.drop(df_cleaning_ema[df_cleaning_ema['VID']== 26].index)
df_cleaning_ema = df_cleaning_ema.drop(df_cleaning_ema[df_cleaning_ema['VID']== 38].index)
df_cleaning_ema = df_cleaning_ema.drop(df_cleaning_ema[df_cleaning_ema['VID']== 111].index)
df_cleaning_ema = df_cleaning_ema.drop(df_cleaning_ema[df_cleaning_ema['VID']== 120].index)
df_cleaning_ema = df_cleaning_ema.drop(df_cleaning_ema[df_cleaning_ema['VID']== 158].index)
df_cleaning_ema = df_cleaning_ema.drop(df_cleaning_ema[df_cleaning_ema['VID']== 160].index)
```

Remove the intro and exit sessions from dataframe for the RSI calculation.

In [101…
```
df_cleaning_ema = df_cleaning_ema[(df_cleaning_ema['Session-ID'] != 0) & (df_cleaning_ema['Session-ID'] != 106)]
```

In [102…
```
df_cleaning_ema.head()
```

Out[102]:

	Name	Scheduled Time	Response Time	VID	Session-ID	Day	Session	weekday	LocationLatitude	LocationLongitude	...	Core-Affect-Valence	Core-Affect-Arousal	LifeSatisfaction	STOPD-1	STOPD-2	STOPD-3
**108**	59986.0	2022-09-14 11:58:56	2022-09-14 13:12:14	2.0	1	1.0	1.0	Wednesday	50.8021	8.7857	...	66.0	11.0	73.0	30.0	59.0	25.0
**109**	59986.0	2022-09-14 14:15:15	2022-09-14 14:39:34	2.0	2	1.0	2.0	Wednesday	50.8021	8.7857	...	74.0	57.0	82.0	16.0	58.0	34.0
**110**	59986.0	2022-09-14 18:11:39	2022-09-14 18:37:03	2.0	3	1.0	3.0	-1.0	-1.0000	-1.0000	...	-1.0	-1.0	-1.0	-1.0	-1.0	-1.0
**111**	59986.0	2022-09-14 20:24:34	2022-09-14 20:32:40	2.0	4	1.0	4.0	Wednesday	50.8021	8.7857	...	79.0	10.0	77.0	6.0	0.0	0.0
**112**	59986.0	2022-09-14 23:39:07	2022-09-14 23:48:30	2.0	5	1.0	5.0	-1.0	-1.0000	-1.0000	...	-1.0	-1.0	-1.0	-1.0	-1.0	-1.0

5 rows × 50 columns

Calculate the RSI value

In [103…
```
subject_list = df_cleaning_ema['VID'].unique()
```

Calculate the median of the EMA sessions for all participants

In [104…
```
subject_median_list = []
for i in subject_list:
    subject_median_list.append(df_cleaning_ema[(df_cleaning_ema['VID']==i)&(df_cleaning_ema['Duration (in seconds)']!=-1)]['Duration (in seconds)'].median())
```

In [105…
```
median_subject_median_list = statistics.median(subject_median_list)
```

Check for each entry of the dataframe, if the RSI is ⩾ 2, and if then delete this entry (set values to -1)

In [106…
```
i = subject_list[0]
for i in subject_list:
    subject_median = df_cleaning_ema[(df_cleaning_ema['VID']==i)&(df_cleaning_ema['Duration (in seconds)']!=-1)]['Duration (in seconds)'].median()
    for index, row in df_cleaning_ema[(df_cleaning_ema['VID']==i)].iterrows():
        if row['Duration (in seconds)'] != -1:
            rsi = subject_median/row['Duration (in seconds)']
            if rsi >= 2:
                df_cleaning_ema.at[index, "weekday"] = -1
                df_cleaning_ema.at[index, "StartDate"] = -1
                df_cleaning_ema.at[index, "EndDate"] = -1
                df_cleaning_ema.at[index, "Progress"] = -1
                df_cleaning_ema.at[index, "Duration (in seconds)"] = -1
                df_cleaning_ema.at[index, "Finished"] = -1
                df_cleaning_ema.at[index, "LocationLatitude"] = -1
                df_cleaning_ema.at[index, "LocationLongitude"] = -1

                df_cleaning_ema.at[index, "PBAT-1"] = -1
                df_cleaning_ema.at[index, "PBAT-2"] = -1
                df_cleaning_ema.at[index, "PBAT-3"] = -1
                df_cleaning_ema.at[index, "PBAT-4"] = -1
                df_cleaning_ema.at[index, "PBAT-5"] = -1
                df_cleaning_ema.at[index, "PBAT-6"] = -1
                df_cleaning_ema.at[index, "PBAT-7"] = -1
                df_cleaning_ema.at[index, "PBAT-8"] = -1
                df_cleaning_ema.at[index, "PBAT-9"] = -1
                df_cleaning_ema.at[index, "PBAT-10"] = -1
                df_cleaning_ema.at[index, "PBAT-11"] = -1
                df_cleaning_ema.at[index, "PBAT-12"] = -1
                df_cleaning_ema.at[index, "PBAT-13"] = -1
                df_cleaning_ema.at[index, "PBAT-14"] = -1
                df_cleaning_ema.at[index, "PBAT-15"] = -1
                df_cleaning_ema.at[index, "PBAT-16"] = -1
                df_cleaning_ema.at[index, "PBAT-17"] = -1
                df_cleaning_ema.at[index, "PBAT-18"] = -1

                df_cleaning_ema.at[index, "Core-Affect-Valence"] = -1
                df_cleaning_ema.at[index, "Core-Affect-Arousal"] = -1

                df_cleaning_ema.at[index, "LifeSatisfaction"] = -1

                df_cleaning_ema.at[index, "STOPD-1"] = -1
                df_cleaning_ema.at[index, "STOPD-2"] = -1
                df_cleaning_ema.at[index, "STOPD-3"] = -1
                df_cleaning_ema.at[index, "STOPD-4"] = -1
                df_cleaning_ema.at[index, "STOPD-5"] = -1
```

Now calculate the RSI rate for each participant and print those who have a RSI rate less than 70%

In [107…
```
for i in range(len(subject_list)):

    all_promts = df_cleaning_ema[df_cleaning_ema['VID'] == subject_list[i]]['Duration (in seconds)'].count() -1
    try:
        missing_promps = df_cleaning_ema[df_cleaning_ema['VID'] == subject_list[i]]['Duration (in seconds)'].value_counts()[-1]
    except:
        missing_promps = 0

    rate= ((all_promts - missing_promps) / all_promts) *100

    if rate < 70:
        print('VID: '+str(subject_list[i]))
        print (rate)
```

```
VID: 27.0
68.26923076923077
VID: 28.0
68.26923076923077
VID: 152.0
69.23076923076923
```

Delete the participants from the dataframe and store the data.

In [108…
```
df_cleaning_ema = df_cleaning_ema.drop(df_cleaning_ema[df_cleaning_ema['VID']== 27].index)
df_cleaning_ema = df_cleaning_ema.drop(df_cleaning_ema[df_cleaning_ema['VID']== 28].index)
df_cleaning_ema = df_cleaning_ema.drop(df_cleaning_ema[df_cleaning_ema['VID']== 152].index)
```

In [109…
```
df_cleaning_ema.to_csv('pocemas_final_dataset_after_cleaning.csv', index=False)
```
